# Chromosome-scale genome assembly provides insights into rye biology, evolution and agronomic potential

**DOI:** 10.1038/s41588-021-00807-0

**Published:** 2021-03-18

**Authors:** M. Timothy Rabanus-Wallace, Bernd Hackauf, Martin Mascher, Thomas Lux, Thomas Wicker, Heidrun Gundlach, Mariana Baez, Andreas Houben, Klaus F. X. Mayer, Liangliang Guo, Jesse Poland, Curtis J. Pozniak, Sean Walkowiak, Joanna Melonek, Coraline R. Praz, Mona Schreiber, Hikmet Budak, Matthias Heuberger, Burkhard Steuernagel, Brande Wulff, Andreas Börner, Brook Byrns, Jana Čížková, D. Brian Fowler, Allan Fritz, Axel Himmelbach, Gemy Kaithakottil, Jens Keilwagen, Beat Keller, David Konkin, Jamie Larsen, Qiang Li, Beata Myśków, Sudharsan Padmarasu, Nidhi Rawat, Uğur Sesiz, Sezgi Biyiklioglu-Kaya, Andy Sharpe, Hana Šimková, Ian Small, David Swarbreck, Helena Toegelová, Natalia Tsvetkova, Anatoly V. Voylokov, Jan Vrána, Eva Bauer, Hanna Bolibok-Bragoszewska, Jaroslav Doležel, Anthony Hall, Jizeng Jia, Viktor Korzun, André Laroche, Xue-Feng Ma, Frank Ordon, Hakan Özkan, Monika Rakoczy-Trojanowska, Uwe Scholz, Alan H. Schulman, Dörthe Siekmann, Stefan Stojałowski, Vijay K. Tiwari, Manuel Spannagl, Nils Stein

**Affiliations:** 1 Leibniz Institute of Plant Genetics and Crop Plant Research (IPK), Seeland, Germany; 2 Institute for Breeding Research on Agricultural Crops, Julius Kühn-Institut, Sanitz, Germany; 3 Plant Genome and Systems Biology (PGSB), Helmholtz-Center Munich, Neuherberg, Germany; 4 University of Zürich, Zurich, Switzerland; 5 Federal University of Pernambuco, Pernambuco, Brazil; 6 Technical University Munich, Munich, Germany; 7 Kansas State University, Manhattan, KS USA; 8 University of Saskatchewan, Saskatoon, Saskatchewan Canada; 9 Canadian Grain Commission, Winnipeg, Manitoba Canada; 10 The University of Western Australia, Crawley, Western Australia Australia; 11 Montana BioAg Inc, Durham, NC USA; 12 ETH Zürich, Zürich, Switzerland; 13 John Innes Centre, Norwich, UK; 14 Institute of Experimental Botany of the Czech Academy of Sciences, Olomouc, Czech Republic; 15 Earlham Institute, Norwich, UK; 16 Institute for Biosafety in Plant Biotechnology, Julius Kühn-Institut, Quedlinburg, Germany; 17 Aquatic and Crop Resource Development, National Research Council, Saskatoon, Saskatchewan Canada; 18 Harrow Research and Development Centre, Agriculture and Agri-Food Canada, Saskatoon, Saskatchewan Canada; 19 Huazhong Agricultural University, Wuhan, China; 20 West Pomeranian University of Technology Szczecin, Szczecin, Poland; 21 University of Maryland, College Park, MD USA; 22 University of Cukurova, Cukurova, Turkey; 23 Sabanci University, Tuzla, Turkey; 24 Saint Petersburg State University, Saint Petersburg, Russia; 25 Vavilov Institute of General Genetics, Russian Academy of Sciences, Saint Petersburg, Russia; 26 TUM School of Life Sciences Weihenstephan, Technical University of Munich, Freising, Germany; 27 Warsaw University of Life Sciences-SGGW, Warsaw, Poland; 28 Chinese Academy of Agricultural Sciences (CAAS), Beijing, China; 29 KWS SAAT SE & Co, Einbeck, Germany; 30 Lethbridge Research and Development Centre, Agriculture and Agri-Food Canada, Lethbridge, Alberta Canada; 31 Noble Research Institute, Ardmore, OK USA; 32 Institute for Resistance Research and Stress Tolerance, Julius-Kühn Institute, Quedlinburg, Germany; 33 Production Systems, Natural Resources Institute Finland (LUKE), Helsinki, Finland; 34 Institute of Biotechnology and Viikki Plant Science Centre, University of Helsinki, Helsinki, Finland; 35 HYBRO Saatzucht GmbH & Co. KG, Isernhagen, Germany; 36 Georg-August-Universität Göttingen, Göttingen, Germany

**Keywords:** Plant breeding, Plant genetics, Genomics, Plant genetics

## Abstract

Rye (*Secale cereale* L.) is an exceptionally climate-resilient cereal crop, used extensively to produce improved wheat varieties via introgressive hybridization and possessing the entire repertoire of genes necessary to enable hybrid breeding. Rye is allogamous and only recently domesticated, thus giving cultivated ryes access to a diverse and exploitable wild gene pool. To further enhance the agronomic potential of rye, we produced a chromosome-scale annotated assembly of the 7.9-gigabase rye genome and extensively validated its quality by using a suite of molecular genetic resources. We demonstrate applications of this resource with a broad range of investigations. We present findings on cultivated rye’s incomplete genetic isolation from wild relatives, mechanisms of genome structural evolution, pathogen resistance, low-temperature tolerance, fertility control systems for hybrid breeding and the yield benefits of rye–wheat introgressions.

## Abstract (web summary)

A chromosome-scale genome assembly of rye inbred line ‘Lo7’ provides insights into its incomplete genetic isolation from wild relatives, mechanisms of genome structural evolution and the yield benefits of rye–wheat introgressions.

## Main

Rye (*Secale cereale* L.), a member of the grass tribe Triticeae and close relative of wheat (*Triticum aestivum* L.) and barley (*Hordeum vulgare* L.), is grown primarily for human consumption and animal feed. Rye is uniquely stress tolerant (biotic and abiotic) and thus shows high yield potential under marginal conditions. This makes rye an important crop along the northern boreal-hemiboreal belt, a climatic zone predicted to expand considerably in Eurasia and North America with anthropogenic global warming^1^. Currently, rye is produced on 4.1 million ha (http://www.fao.org/faostat/en/, accessed June 2020), 81% of which is in northeastern Europe. More importantly, however, rye chromatin is commonly introgressed into bread wheat varieties to improve yield and thus rye genetic material is present in a far greater proportion of cultivated land area^2–5^. Rye is a diploid with a large genome (~7–8 gigabases, Gb)^6^, 50% larger than the syntenic diploid barley and bread wheat subgenomes^7^. Like barley and wheat, rye entered the genomics era very recently. A virtual gene-order was released in 2013^8^ and a shotgun de novo genome survey of the same line became available in 2017^9^. Both resources have been rapidly adopted by researchers and breeders^10–12^ but cannot offer equivalent opportunities to the high-quality genome assemblies available for other Triticeae species^7,13–17^.

We report a short-read based chromosome-scale genome assembly for rye inbred line ‘Lo7’ and demonstrate the potential of this new genomic resource by dissecting the incomplete genetic isolation of rye from wild relatives. We showcase detailed analyses of the genomic organization and complexity of gene families implicated in stress tolerance and pollen fertility. This resource will guide future rye breeding and provide immediate benefit in managing the trade-offs of using rye as a genetic resource in wheat crop improvement.

## Results

### Genome assembly, validation and annotation

We de novo assembled scaffolds representing 6.74 Gb of the estimated 7.9 Gb ‘Lo7’ genome from >1.8 Tb of short-read sequence (Methods; Supplementary Table 1 and Supplementary Note). These scaffolds were ordered, oriented and curated using: (1) chromosome-specific shotgun (CSS) reads^8^, (2) 10x Chromium linked reads, (3) genetic map markers^9^, (4) three-dimensional chromosome conformation capture sequencing (Hi-C)^18^ and (5) a Bionano optical genome map (Supplementary Tables 2–7). After intensive manual curation (Supplementary Note), 92% of this assembled sequence (~78% of the estimated genome size) was arranged first into super-scaffolds (N50 > 29 megabases, Mb) and then into pseudomolecules. Shotgun reads (~947 Gb of data, ~120× mean depth-of coverage) were mapped back to the assembly to confirm a near-unimodal coverage distribution consistent with a high-quality assembly (Supplementary Table 8 and Supplementary Note). De novo annotation (Methods; Supplementary Table 9) yielded 34,441 high-confidence (HC) genes, including 96.4% of the BUSCO (v.3) near-universal single-copy ortholog set (Supplementary Table 1), 19,456 full-length DNA long terminal repeat (LTR) retrotransposons (LTR-RTs) from six transposon families (Supplementary Table 10)^19^, 13,238 putative microRNAs (miRNAs) in 90 miRNA families (Supplementary Tables 11–17) and 1,382,323 tandem repeat arrays (Supplementary Tables 18 and 19). Full-length LTR-RTs represent a similar proportion of the total assembly in relation to genome size as shown by other recent Triticeae chromosome-scale assemblies (Supplementary Note and Supplementary Table 20) providing further evidence for high assembly quality and completeness^20^. Fluorescence in situ hybridization (FISH) to mitotic rye chromosomes confirmed agreement between in silico predicted and true physical distribution of distinct low- and high-copy probe sequences (Methods; Supplementary Note and Supplementary Table 21).

**Fig. 1:**
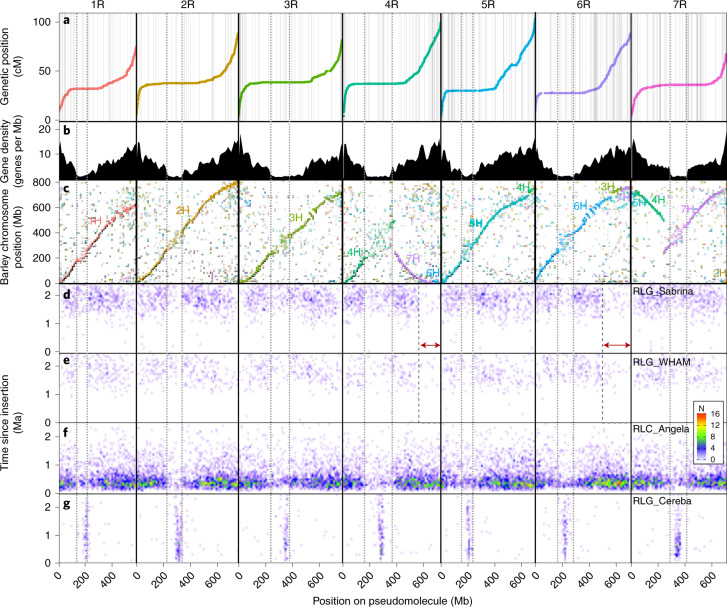
Rye (‘Lo7’) genome composition and structure over chromosomes 1R to 7R. Twin vertical gray lines in each chromosome denote the boundaries of the pericentromeric low-collinearity regions for each chromosome. **a**, Genetic map positions of markers used in assembly. Scaffold boundaries marked by gray vertical lines. **b**, Density of annotated gene models. **c**, Gene collinearity with barley (cv. ‘Morex’), with the position on the ‘Morex’ pseudomolecules on the vertical axis. Text and point colors represent barley chromosomes as labeled. **d**–**g**, Positions and ages of four LTR retrotransposon families RLG-Sabrina (**d**), RLG-WHAM (**e**), RLC-Angela (**f**) and RLG_Cereba (**g**) in the genome, represented as a heatmap. Binned ages are on the vertical axis (from 0 million years ago, Ma, at the bottom) and bin positions are across the horizontal. Heat represents the number of TEs in each age/position bin (see legend inset). Red arrows mark notable changes in LTR-RT profiles.

The rye genome follows similar organization as previously reported for other Triticeae genomes^7,13^ (Fig. 1 and Supplementary Note): chromosomes are lacking recombination over ~50% of their physical length (Fig. 1a) and gene density increases by a factor of >10 towards the telomeres (Fig. 1b).

Gene collinearity plots (Fig. 1c and Supplementary Notes) between rye (‘Lo7’), barley (cv. ‘Morex’) and wheat (cv. ‘Chinese Spring’), confirm, with the exception of the gene-scarce zones surrounding centromeres, extensive genome collinearity. Genome expansion occurred rather uniformly over most of the chromosome arms with some acceleration toward distal regions, reflected by collinearity plots curving towards the telomeres (Supplementary Note). This expansion might be attributed predominantly to activity of LTR-RT families affecting the intergenic space^21,22^. We therefore estimated the time of highest insertion activity for the most frequent rye LTR-RT families RLG_SABRINA, RLG_WHAM and RLC_ANGELA (Methods; Fig. 1d–g and Supplementary Note). RLC_ANGELA elements did recently target this genomic niche and older RLG_SABRINA and RLG_WHAM expansions affected more proximal parts of the chromosome arms. Two distal regions on the long arms of rye chromosomes 4R and 6R, however, differed by a lack of the more ancient activity of the RLG_SABRINA and RLG_WHAM families, possibly highlighting regions affected by ancient translocation events from a rye with a different retrotransposon landscape to ‘Lo7’ (Supplementary Note). RLG_CEREBA elements (Fig. 1g) were active in centromeres, acting more constantly over longer time scales than the other frequent LTR-RT families.

### Rye genome evolution

#### Large structural variations—mechanisms of genetic isolation

**Fig. 2:**
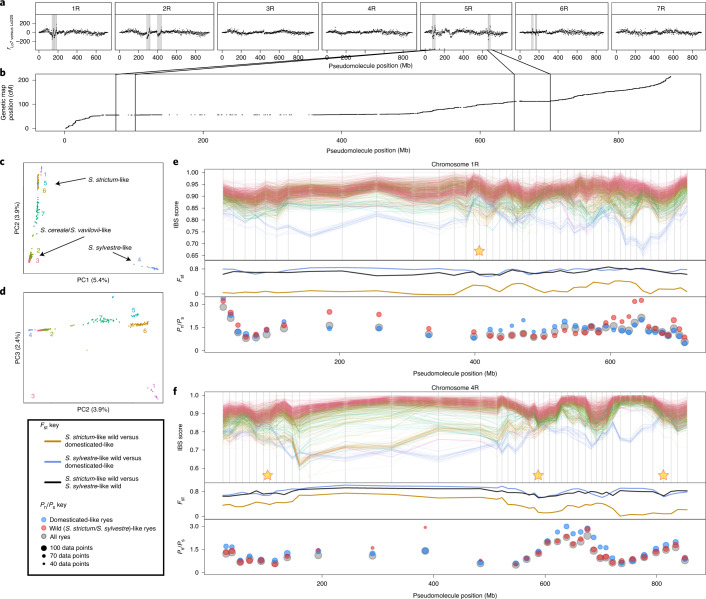
Dissecting the relationships among rye genotypes. **a**, Hi-C asymmetry detects SVs between the reference genotype ‘Lo7’ and *S. cereale* ‘Lo225’. SVs result in discontinuities in *r*, the ratio of Hi-C links mapping left:right relative to ‘Lo7’. Large inversions (marked) typically produce clean, diagonal lines. Visually identified candidate SVs are shaded. **b**, Detail of 5R genetic map marker positions showing how recombination rate relates to candidate SVs. The rightmost inversion marked on 5R corresponds to a region of suppressed recombination on chromosome 5R. The effect of other ‘Lo7’ versus ’Lo225’ SVs on recombination was harder to confirm since they fall in already-low-recombining regions. **c**,**d**, PCA plots showing the relationships among genetically determined rye clusters for PCs 1 and 2 (**c**) and for PCs 2 and 3 (**d**). **e**,**f**, Binwise IBS, *F*_st_ and *P*_n_/*P*_s_ statistics calculated across the chromosomes using the expanded Schreiber et al.^25^ rye diversity panel data mapped to the ‘Lo7’ assembly. Exemplary instances are shown: chromosomes 1R (**e**) and 4R (**f**). The position of each bin on the genome is the mean pseudochromosome position of identified variable sites within that bin. Upper in each pane: binwise IBS scores of the panel genotypes compared with ‘Lo7’, with features discussed in the text marked with asterisks. Colors correspond to **d**. Middle in each pane: binwise *F*_st_ showing changes in genetic variance partitioning among and between subgroups across the chromosome. Line colors, *F*_st_ key. Lower in each pane: binwise *P*_n_/*P*_s_ ratios (shown when *P*_n_ + *P*_s_ > = 10 for a given bin) for recently acquired rye polymorphisms (wheat outgroup). The values were calculated separately for different groups of ryes (‘domesticated’ (cluster 3) versus ‘wild’ (clusters 1,4–7)—see **d**) to allow detection of possible recent selective events affecting different rye groups. Point colors/sizes, *P*_n_/*P*_s_ key.

Megabase-scale inversions are a common feature of structural variation (SV) in the related barley genome^23^. In the absence of multiple rye genome assemblies, we sought to make a first survey of large SV prevalence among rye cultivars and wild relatives using three-dimensional conformation capture sequencing (Hi-C; Methods; Supplementary Note)^23^. In the comparison between two cereal rye cultivars ‘Lo7’ and ‘Lo225’, representing the two distinct heterotic gene pools in hybrid-rye breeding, megabase-scale inversions are apparent on four of the seven rye chromosomes (Fig. 2a and Supplementary Note). Among them, a 50-Mb inversion (comprising 382 HC genes) on chromosome 5R (positions ~650–700 Mb), coincides with a region lacking genetic recombination (Fig. 2b), providing genetic corroboration for its presence. This observation points to a previously undocumented source of unwanted linkage drag potentially affecting rye breeding efforts. Large inversions between ‘Lo7’ and other *Secale* representatives in the sample increase in number dependent on the phylogenetic distance to *S. cereale* and occur preferentially in the pericentromeric low-collinearity regions (*P* < 0.001, one-tailed empirical distribution derived from 10,000 simulations; Supplementary Note). Large SVs therefore provide a potential mechanism for localized collinearity loss between the pericentromeric regions of Triticeae species. This collinearity loss provides, in turn, at least one mechanism for effective genetic isolation during speciation^24^.

#### Reticulate evolution of rye

Rye’s divergence from its close relatives wheat and barley has not been comprehensively resolved. Using a draft assembly, Martis et al.^8^ interpreted variation in sequence identity between rye and barley along chromosomes as a possible indicator of ancient species hybridization creating a ‘mosaic’ genome. Genome-wide estimations of fixation indices (*F*_st_) and ABBA-BABA-based *D*-statistics have reflected varying levels of recent genetic exchange among *Secale* groups^25^. We confirmed, using *D*-statistics, that directional gene flow has occurred between rye groups (Methods; Supplementary Table 22). We then extended Martis et al.^8^ sequence identity approaches with higher resolution proxies measurable across the chromosomes. Reticulation reduces the evolutionary distance between individuals of each species at any site where chromatin was secondarily exchanged, causing phylogenetic discordance among loci. Using the sequence identity of reciprocal best BLAST matches between rye CDS sequences and CDS sequences from barley cv. ‘Morex’^13^ and wheat cv. ‘Chinese Spring’^7^(Methods; Supplementary Note), we found no strong evidence of discordance among these genera: rye is more closely related to the bread wheat genomes than to barley across the whole genome (Table 1).

**Table 1:** Genome assembly and annotation statistics

Assembly	Raw scaffolds (after chimera breaking)	In chromosome-scale pseudomolecules	
Scaffolds	109,776	476	
Total length (Mb)	6,670.03	6,206.74	
N50 length (Mb)	15.16	29.44	
Length with chromosome assignment (%)	95.3%	100%	
**Optical genome map**			
Maps	5,601		
Total length (Mb)	6,660.18		
N50 length (Mb)	1.671		
**Assembly/optical map alignment**			
Total aligned length (Mb)	6,248.60		
Uniquely aligned length (Mb)	6,029.11		
**Gene feature annotation**	**High-confidence (HC) set**	**Low-confidence (LC) set**	
Number of genes	34,441	22,781	
Mean gene length	2,892	946	
Mean exons per gene	4.42	1.79	
Proportion of complete BUSCO set	96.4%	5.8%	
**LTR-RT annotation**	**Superfamily**	**Full-length copies**	**Mean age (Ma)**
*RLC_Angela*	Copia	11,128	0.53
*RLG_Cereba*	Gypsy	934	1.24
*RLG_Sabrina*	Gypsy	3,996	2.10
*RLG_WHAM*	Gypsy	1,457	2.06
*DTC_Clifford*	CACTA	1,480	NA
*DTC_Conan*	CACTA	516	NA
*RLC total*	NA	13,124	NA
*RLG total*	NA	1,973	NA
*LTR-RT total*	NA	15,097	NA

BUSCO, benchmarking universal single-copy orthologs (v.3; https://busco.ezlab.org/); NA, not applicable.

We then produced an analogous analysis for *Secale* species by calculating identity-by-state (IBS) statistics between ‘Lo7’ and sequence data from a population of 955 cultivated and wild ryes (dataset of Schreiber et al.^25^, expanded here by a further 352 genotypes; Methods). We used *k*-means clustering to define seven rye genetic clusters (Fig. 2c,d). In contrast to the intergenus comparisons, recent reticulation among rye clusters was strongly supported. In general, ‘Lo7’ is most closely related to *S. cereale* and *S. vavilovii*-dominated clusters and successively less related to *S. strictum*-like clusters and most distant from the *S. sylvestre-*dominated cluster (Fig. 2d; Supplementary Note). However, clear departure from this pattern occurs frequently (Fig. 2e,f and Supplementary Note). For example, at regions on chromosomes 1R and 4R (marked on Fig. 2e,f), *S. sylvestre*-like individuals are closely related to ‘Lo7’, often more closely even than some *S. strictum*-like individuals, suggesting recent genetic exchange between *S. cereale*-like and *S. sylvestre*-like genotypes. Pairwise *F*_st_ was calculated to assess the proportions of genetic variability within and between cluster groups and shows considerable variability across the chromosomes, especially comparing within- and between-group variability among *S. strictum*-like and ‘domesticated’-like clusters (Fig. 2d–f). On chromosome 4R, for instance, *F*_st_ is almost 0.8 along the pericentromeric region (~200–400 Mb) but approaches zero at two interstitial positions (~600 and 720 Mb), corroborating incomplete genetic separation between these subgroups.

To investigate the effects of incomplete genetic isolation on recent selection pressures exerted on domesticated rye, we examined the ratio of nonsynonymous to synonymous mutations in exonic single nucleotide polymorphisms (SNPs) segregating among ryes (*P*_n_/*P*_s_) but which shared an allele with the consensus state of the three bread wheat genomes (a proxy for the ancestral state), thus surveying primarily recent mutations within rye lineages (Methods). Under equivalent selective regimes, *P*_n_/*P*_s_ values for wild and domesticated ryes are expected to be approximately equal but we observed *P*_n_/*P*_s_ divergence in the low-collinearity pericentromeric regions of chromosomes 1R, 3R and 4R. This divergence probably reflects the reduced efficacy of selection in such regions where recombination is limited (for example, by SVs).

On the basis of these collected observations, we concur with the Martis et al.^8^ hypothesis that the genome of cereal rye is a mosaic in the sense that different rye species are not completely reproductively isolated; however, we did not produce any evidence to suggest that the mosaic involves intergeneric hybridization.

#### Tracking the fate of rye chromatin in wheat improvement

**Fig. 3:**
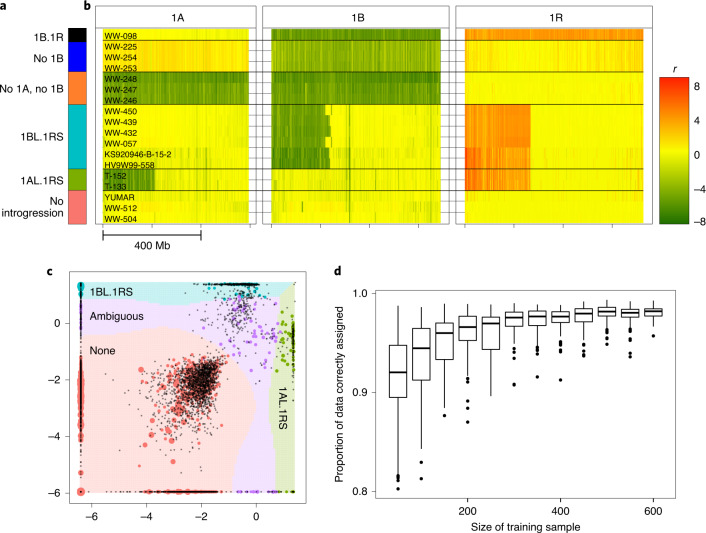
Combined reference mapping as a means to classify wheat and wheat–rye introgression karyotypes. **a**, Color key for **b** and **c**. **b**, Normalized read mapping depths for 1-Mb bins of chromosomes 1A, 1B and 1R, for a selection of wheat lines (including also some *Aegilops tauschii* accessions which contain no A or B subgenome) with various chromosome complements and introgressions (rows). The value *r* denotes the difference between the log_2_ reads per million mapping to a bin, compared to *T. aestivum* cv. ‘Chinese Spring’. **c**, Visual representation of the SVM classifier, with the two selection features based on relative read mapping densities to ‘translocation-prone’ and ‘other’ chromosomal regions (Methods) shown on the *x* and *y* axes. Points represent training samples, with color corresponding to human-designated classification and size proportional to the total number of mapped reads for the sample. Black points are samples not classified by a human. Background colors represent the hypothetical classification that would be given to a sample at that position. **d**, Results of cross-validation testing the accuracy of the classifier and its relationship to the size of the training set. Box edges and whiskers represent quartiles and the center lines show the arithmetic means.

The transfer of rye genetic material into bread wheat can provide substantive yield and stress tolerance benefits^26^, though at the expense of bread-making quality^27^. These transfers involved a single 1BL.1RS Robertsonian translocation originating from cv. ‘Kavkaz’ and a single 1AL.1RS translocation from cv. ‘Amigo’ (Fig. 3)^3,4^. Due to the trade-off between yield and quality, wheat breeders must screen their programs for rye introgressions. Taking advantage of the new rye assembly, we implemented a high-throughput sequencing-based approach on four expansive wheat germplasm panels (Kansas State University (KSU), United States Department of Agriculture Regional Performance Nursery (USDA-RPN), International Maize and Wheat Improvement Center (CIMMYT), Wheat and Barley Legacy for Breeding Improvement (WHEALBI); Methods) segregating for both 1AL.1RS and 1BL.1RS. Translocations can be observed as obvious changes in normalized read depth across both the translocated and replaced chromosomal regions (Fig. 3b and Supplementary Note).

Human classification of a whole panel of karyotypes is still costly in terms of time. To alleviate this bottleneck, we developed an automated support vector machine (SVM) classifier that replicates human assignment with over 97% accuracy (Methods; Fig. 3c,d). We then demonstrated that the automated classifications predict yield. A mixed-effects linear model applied to yield data available for the autoclassified individuals in the KSU (*n* = 19,677) and USDA (*n* = 29,035) breeding panels showed that 1R introgressions could increase average yields up to ~4.55% (Table 2; Methods; Supplementary Tables 23–25). The 1AL.1RS karyotype significantly outyielded 1BL.1RS in the KSU panel but the reverse was true of the USDA panel (Table 2). This is probably due to the effects of foreign chromatin being highly nonuniform and influenced by diverse factors (Supplementary Note), in particular the wheat genetic background^27,28^. Taking advantage of the ‘Lo7’ chromosome-scale assembly, tracking of rye chromatin in wheat breeding programs will now become more reliable and predictable.

**Table 2:** Summary of fixed effects estimates from linear mixed model estimating the influence of rye–wheat translocations upon yield, in two wheat diversity panels

Panel	Introgression type	Estimated yield effect	s.e.	*t*	Degrees of freedom	*P*
KSU	1AL.1RS	4.06%	0.54	7.54	1.89 × 10^4^	4.95 × 10^−14^
KSU	1BL.1RS	1.50%	0.41	3.68	1.88 × 10^4^	0.00023
USDA	1AL.1RS	0.86%	0.31	2.72	2.82 × 10^4^	0.0064
USDA	1BL.1RS	4.55%	0.39	11.78	2.82 × 10^4^	< 2.0 × 10^−16^

*P* values are calculated using a one-sided Student’s *t*-test on the null hypothesis that the true yield effect is zero (Methods; Supplementary Table 25).

### Rye vigor is in the genes

Rye distinguishes itself from other Triticeae through strong allogamy, which facilitates commercial hybrid-rye breeding, as well as conferring resilience to biotic stress and extreme winter-hardiness, qualifying rye as an important plant genetic resource in wheat improvement. Here, we showcase how the high-quality genome assembly sheds light on the genetic control of these specific aspects of rye biology.

#### Fertility restoration in rye and wheat

**Fig. 4:**
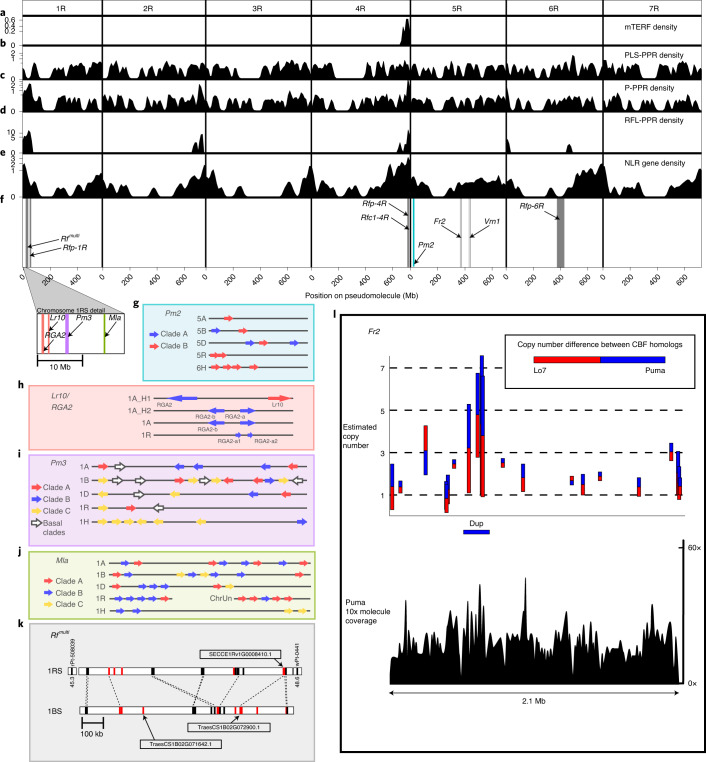
Comparative genomics of rye genes with agricultural importance. **a**–**e**, Density (instances per Mb) of mTERFs (**a**), PPRs (**d**) as well as NLRs (**e**) across the pseudomolecules. For visualization, the *y* axis is transformed using *x* → *x*^1/3^. **f**, Genomic locations of genes and loci discussed in the text. Colored bars correspond and refer to the colors of the box outlines in **g**–**k**; **g**–**j**, physical organization of selected NLR gene clusters compared across cultivated Triticeae genomes: *Pm2* (**g**), *Lr10/RGA2* (**h**), *Pm3* (**i**) and *Mla* (**j**). **k**, Organization of RFL genes at the ‘Lo7’ *Rf*^*multi*^ locus compared to its wheat (‘Chinese Spring’) counterpart. Flanking markers are shown on either end of the rye sequence. Two full-length wheat RFLs and a putative rye ortholog are labeled. PPR genes are colored red. **l**, CNV between ‘PUMA-SK’ and ‘Lo7’ within the *Fr2* interval revealed by 10x Genomics linked read sequencing. A (Dup)lication flagged by the Loupe analysis software is marked. The estimated copy number differences between ‘Lo7’ and ‘Puma’ are shown for *CBF* genes.

Rye hybrid breeding relies on efficient cytoplasmic male-sterility (CMS)/restorer-of-fertility (Rf) systems; however, the underlying molecular mechanisms have yet to be elucidated. Known *Rf* genes belong to a distinct clade of the pentatricopeptide repeat (PPR) RNA-binding factor family, referred to as *Rf*-like (RFL)^29,30^. Members of the mitochondrial transcription TERmination factor (mTERF) family are probably also involved in male fertility restoration in cereals^31,32^. The ‘Lo7’ assembly reveals a PPR-RFL/mTERF hotspot on 4RL coinciding with known *Rf* loci for two rye CMS systems known as CMS-P (the commercially predominant ‘Pampa’-type) and CMS-C^12,33–35^(Methods; Fig. 4a–f, Supplementary Tables 26 and 27 and Supplementary Note). We determined, as previously suggested, that these two loci, *Rfp* and *Rfc*, are closely linked but physically distinct^36^ (Supplementary Table 28). Two members of the PPR-RFL clade reside within 0.186 Mb of the *Rfc1* locus (Supplementary Tables 26–28). The *Rfp* locus, in contrast, is neighbored by four *mTERF* genes (Supplementary Tables 27–28), in agreement with previous reports that an mTERF protein represents the *Rfp1* candidate gene in rye^32,37^.

The new assembly also helped to dissect a strong candidate gene for the wheat locus *Rf*^*multi*^ (*Restoration-of-fertility in multiple CMS systems*) on wheat chromosome 1BS. Replacement of the wheat *Rf*^*multi*^ locus by its rye ortholog using 1RS.1BL chromosome translocations produces the male-sterile phenotype^38,39^. At the syntenic position of *Rf*^*multi*^, wheat and rye share a PPR-RFL gene cluster^7^ (Fig. 4k, Supplementary Table 26 and Supplementary Note). Only two wheat RFL-PPR genes in the cluster, *TraesCS1B02G071642.1* and *TraesCS1B02G072900.1*, encode full-length proteins; only the latter corresponds to a putative rye ortholog (*SECCE1Rv1G0008410.1*). Thus, the absence of a *TraesCS1B02G071642.1* ortholog in the nonrestorer rye suggest it as an attractive *Rf*^*multi*^ candidate. Current implementations of a wheat–rye *Rf*^*multi*^ CMS system involve 1RS.1BL translocations^5,40,41^, which are typically linked to reduced baking quality^27^. Efforts to break this linkage may now benefit from marker development and/or genome editing approaches targeting *TraesCS1B02G071642.1* (ref. ^42^).

#### Divergence of disease resistance loci in Triticeae

Rye plays an important role as genetic resource of biotic stress tolerance in wheat varieties carrying rye chromatin insertions. Race-specific pathogen resistance is typically associated with members of the class of nucleotide-binding-site and leucine-rich repeat (NLR)-motif genes^43^. We annotated 792 full-length rye NLR genes, finding them enriched in distal chromosomal regions, similar to what has been seen recently in the bread wheat genome^7,44^ (Fig. 4e and Supplementary Tables 29 and 30). We compared the genomic regions in rye that are orthologous to mildew (*Pm2*, *Pm3* and *Mla*) and rust (*Lr10*) resistance gene loci from wheat and barley (Fig. 4g–j, Supplementary Table 31 and Supplementary Note). All loci, except for *Lr10*, contained complex gene families with several subfamilies either present or absent in individual genomes, indicating either functional redundancy or the evolution of distinct resistance specificities or targets. For example, the wheat *Pm3* and rye *Pm8*/*Pm17* genes are orthologs and belong to a subfamily (clade A, Fig. 4i) which is absent in barley, whereas a different distinct subfamily (clade B, Fig. 4i) of the *Pm3* genes is present in wheat and barley but absent in rye (Supplementary Note). In essence, the ‘Lo7’ assembly reveals the genomic organization of conserved or nonorthologous NLR gene clusters, which can be exploited in future rye and wheat improvement efforts.

#### Frost tolerance

Rye possesses superior low-temperature tolerance (LTT) to other Triticeae crops^45^. A syntenic locus *Fr2* comprising a cluster of CBF (C-repeat/DRE-binding factor) genes is present on Triticeae group 5 chromosomes controlling LTT^46^ in rye^47^, *T. monococcum*^48^, bread wheat^49,50^ and barley^51^. In cold-tolerant varieties, LTT-implicated CBF genes of the *Fr2* locus are transcriptionally upregulated^52,53^. In ‘Lo7’, the *Fr2* locus comprises a cluster of 21 CBF-related genes at 614.3–616.5 Mb on 5R (Fig. 4f and Supplementary Table 32).

Since CBF gene family expansion correlates with increased LTT in other Triticeae^54^ (Supplementary Note), we analysed phased-linked-read (10x Genomics Chromium) data of an *Fr2*-homozygous line with exceptional LTT (‘Puma-SK’ derived from rye variety ‘Puma’) in comparison to ‘Lo7’ (low LTT). Four of the *Fr2* CBF genes, all members of the same CBF subfamily (Supplementary Note) for which CNV has been previously implicated in LTT in wheat^54^, showed patterns of copy number variation (CNV) (*SECCE5Rv1G030450*, *SECCE5Rv1G030460*, *SECCE5Rv1G030480* and *SECCE5Rv1G030490*; Supplementary Table 33 and Supplementary Note).

**Fig. 5:**
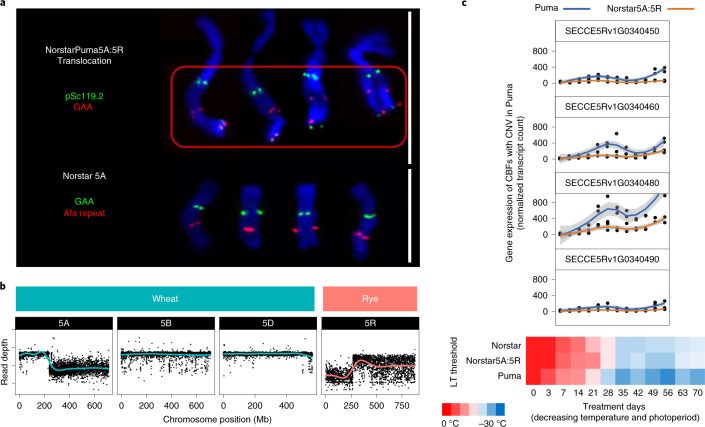
The cold tolerance associated region *Fr2* in ‘Puma’ and ‘NorstarPuma5A:5R’ translocation line. **a**, Chromosome labeling (top) using probes specific for ‘Norstar’ chromosome 5A (Afa) and ‘Puma’ 5R (pSc119.2) confirm the presence of a rye translocation in NorstarPuma5A:5R (red box), which also alters the binding of GAA. White bars, 10 µm. **b**, Combined reference read mapping of group 5 chromosomes confirms the balanced translocation event, gain of a large region of chromosome 5R from ‘Puma’ (rye, light read line) and loss of a large region on chromosome 5A of ‘Norstar’ (wheat, light blue line) in ‘NorstarPuma5A:5R’. Read depth is given in log_2_ reads per million versus ‘Chinese Spring’. **c**, Gene expression analysis of rye CBF genes with CNV in ‘Puma’ (blue line) and ‘NorstarPuma5A:5R’ (orange line). Plants were grown in a time series with decreasing day length and temperature over a 70-d period and the temperatures at which 50% lethality was observed (LT50) were recorded (heatmap).

Transferring superior LTT from rye to wheat by translocation is an attractive breeding goal. We derived a 5A.5RL translocation line in winter wheat ‘Norstar’ using ‘Puma’ rye as the 5R donor, thus replacing the wheat 5A CBF cluster (Methods; Fig. 5a,b). LTT, however, was not notably altered by the translocation compared to ‘Norstar’ (Fig. 5c), suggesting that the rye CBF gene cluster is activated but, as previously suggested by Campoli et al.^53^, differently regulated in the wheat background. Gene expression of ‘Puma’ CBFs with CNV were indeed attenuated during treatments of cold stress in ‘Norstar5A:5R’ (Methods; Fig. 5c). Therefore, transferring LTT from rye into wheat will require indepth understanding of differences in the LTT regulatory network between rye and wheat.

## Discussion

The high-quality chromosome-scale assembly of rye inbred line ‘Lo7’ constitutes an important step forward in genome analysis of the Triticeae crop species and complements the resources recently made available for different wheat species^14,55–58^ and barley^13,59^. This resource will help reveal the genomic basis of differences in major life-history traits between the self-incompatible, cross-pollinating rye and its selfing and inbreeding relatives. Our evolutionary analyses demonstrate that rye subspecies are better conceptualized as a reticulated group of incipient species and that mechanisms such as transposable-element expansion and SV between genotypes are probably acting to bring about evolutionary divergence. The joint use of the rye and wheat genomes to characterize the effects of rye chromatin introgressions may provide a short-term opportunity to breeders as they continue to better separate confounding variables from the genetic combinations that best improve yield in various environments; but these benefits will ultimately be affected by negative linkage so long as whole chromosome arm translocations are involved. Discoveries at the single-gene level—such as the contributions offered here to pathogen resistance, LTT and male fertility restoration control—will be best tested and exploited by finer-scale manipulation in dedicated experiments^12^. This is an indispensable prerequisite for the development of gene-based strategies that exploit untapped genetic diversity in breeding materials and ex situ gene banks to improve small grain cereals and meet the changing demands of global environments, farmers and society.

## Methods

### Genome size estimation by flow cytometry

We characterized the landscape of *S. cereale* genome sizes to contextualize the size of the ‘Lo7’ genome and gain an impression of genome size variation within the species. Grains from 15 diverse rye accessions were from nine providers listed in Supplementary Table 1. Plants of pea (seeds provided by Semo breeding station, Smržice, Czech Republic) served as an internal reference standard in flow cytometric estimation of nuclear DNA content in all accessions, except of the tetraploid accession ACE-1, for which *S. cereale* line ‘Lo7’ was used as a reference. Plants were raised in garden compost in pots and maintained in a greenhouse until they reached a height of 10–20 cm. Nuclear genome size was estimated essentially as described by Doležel et al.^60^ using a CyFlow Space flow cytometer (Sysmex Partec) equipped with a 532-nm green laser. The gain of the instrument was adjusted so that the peak representing interphase first growth (G1) nuclei of the standard was positioned approximately on channel 100 on a histogram of relative fluorescence intensity when using a 512-channel scale. Five individual plants per test species were sampled and each sample was analysed three times, each time on a different day. A minimum of 5,000 nuclei per sample was analysed and 2C DNA contents in pg were calculated from the means of the G1 peak positions by applying the following formula:

$$2{\mathrm{C}}\,{\mathrm{nuclear}}\,{\mathrm{DNA}}\,{\mathrm{content}}\frac{{{\mathrm{ = sample}}\,{\mathrm{G}}1\,{\mathrm{peak}}\,{\mathrm{mean}} \times {\mathrm{standard}}\,2{\mathrm{C}}\,{\mathrm{DNA}}\,{\mathrm{content}}}}{{{\mathrm{standard}}\,{\mathrm{G}}1\,{\mathrm{peak}}\,{\mathrm{mean}}}}$$

Mean nuclear DNA content (2C) was then calculated for each accession. DNA contents in pg were converted to genome size in bp using the conversion factor 1 pg DNA = 0.978 Gb (ref. ^61^). Statistical analysis was performed using NCSS 97 statistical software package (Statistical Solutions). One-way analysis of variance and a Bonferroni’s (all pairwise) multiple comparison test were used for analysis of variation in monoploid (1C×) genome size. A significance level *α* = 0.01 was used.

### ‘Lo7’ genome assembly and gene annotation

Descriptions of the assembly methods, descriptions of the data generation and the annotation procedure for gene features, are given in the Supplementary Note.

### Fluorescence in situ hybridization (FISH)

Three-day-old roots of the rye accession were pretreated in 0.002 M 8-hydroxyquinoline at 7 °C for 24 h and fixed in ethanol:acetic acid (3:1 v/v). Chromosome preparation and FISH were performed according to the methods described by Aliyeva-Schnorr et al.^62^. The hybridization mixture contained 50% deionized formamide, 2× SSC, 20% dextran sulfate and 5 ng µl^–1^ of each probe. Slides were denatured at 75 °C for 3 min and the final stringency of hybridization was 76%. We used 34–65 nucleotide-long 5′-labeled oligo probes designed for the in silico identified repeats and published probe sequence (Supplementary Table 21). Images were captured using an epifluorescence microscope BX61 Olympus equipped with a cooled CCD camera (Orca-ER, Hamamatsu). Chromosomes were identified visually on the basis primarily of morphology, heterochromatic DAPI + bands and the localization of probe pSc119.2.1 (ref. ^63^) (Supplementary Note).

### Gene-level synteny and percentage identity scores between rye and other Triticeae species

HC gene sequences from the ‘Lo7’ gene annotation were aligned to the annotated transcriptomes of bread wheat^7^ (*T. aestivum* cv. ‘Chinese Spring’) and barley^13^ (*H. vulgare* cv. ‘Morex’) using BLASTn (v.2.9.0+)^64^ with default parameters. The lowest E-value alignment for each gene against the transcriptome associated with each subject genome (or subgenome) was selected, with the highest bitscore and then longest alignment chosen in the case of a tie. Only reciprocal best matches per (sub)genome were accepted. Relative evolutionary distances between rye, barley and the wheat subgenomes were estimated using the mean percentage identity scores of these filtered matches, calculated in bins of 100 reciprocal matches (in increments of 20 bins). The positions of the bins on the pseudomolecules were taken to be the mean match position of the matches within each bin.

### Phylogenetic analyses, IBS statistics, *F*_st_, *D*-statistics and *P*_n_/*P*_s_

The genotyping-by-sequencing (GBS) dataset of 603 samples from Schreiber et al.^25^ was extended by a 347 further GBS samples from the IPK gene bank (mainly wild *Secale* taxa) and the five samples used in the Hi-C SV-detection study (‘Lo7’, ‘Lo225’, ‘R1003’, ‘R925’ and ‘R2446’). The resulting sample set (*n* = 955) and passport data are listed in Supplementary Table 34. DNA isolated from the five Hi-C samples was sent to Novogene (en.novogene.com/) for Illumina library construction and sequencing in multiplex on the NovaSeq platform (paired-end 150-bp reads, ~140 Gb per sample, S2 flow cell). Demultiplexing, adapter trimming, read mapping and variant calling correspond to the approach described in Schreiber et al.^25^, using the new reference for read mapping. The dataset was filtered for a maximum of 30% missing data and a minor allele frequency of 1% resulting in 72,465 SNPs was used (Supplementary Note). A neighbor joining tree was constructed with the R package ‘ape’^65^, on the basis of genetic distances computed with the R package SNPRelate^66^. Principal component analysis (PCA) was performed with smartPCA from the EIGENSOFT v.6.0.1 package (github.com/DReichLab/EIG) using least square projection without outlier removal. Seven rye genetic clusters were designated using the ‘kmeans’ R function, with default parameters, using the first three principal components from the PCA as input.

For IBS and *F*_st_ analyses, a more stringent filtering regime requiring read depth ≥ 6, maximum 5% missing data and call quality ≥ 250 (resulting in 9,538 SNPs) was selected (Supplementary Note). IBS scores between ‘Lo7’ and the other lines in the set were calculated in windows of 100 consecutive SNP loci (at intervals of 25 SNP loci) using the snpgdsIBS function in the R package SNPRelate. *F*_st_ was calculated in the same windows using the snpgdsFst function in the R package SNPRelate (using method=’W&H02’). The calculation was performed for every pairwise combination of the following groups: ‘Domesticated-like’ (cluster 3), ‘Wild–*S. strictum*-like’ (clusters 1, 5 and 6) and ‘Wild–*S. sylvestre*-like’ (cluster 4).

To assign ancestral states to variant SNPs segregating in rye, exonic variants identified in the GBS dataset were coupled to their orthologs alleles in the three bread wheat (‘Chinese Spring’)^7^ alleles using the rye-versus-wheat CDS BLAST alignments (see above) and parsing the BLAST alignment strings using the custom script blast_get_alleles_at_position.c (https://github.com/mtrw/tim_bioinfo_tools). Reciprocal best matches were calculated separately for the alignments between the rye CDS set and the set of CDSs from each wheat genome. The ancestral allele was assigned by consensus among the wheat genomes and, if no allele claimed a majority, the variant was omitted from the dataset. Genome-wide *D*-statistics were calculated according to the four-taxon ABBA-BABA method as described in ref. ^67^, with the wheat consensus allele as the outgroup and selections of the *k*-means-assigned clusters selected as the three test populations. Estimator variance was approximated via the block jackknife procedure, with 5 Mb exclusion bins. The effects of the rye-versus-wheat nucleotide differences falling within coding sequences were annotated using SnpEff (v.4, ‘ann’ function), with default parameters. *P*_n_/*P*_s_ scores (the ratio of counts of nonsynonymous to synonymous differences to wheat in variants segregating in rye) were calculated using the same binning scheme as was used for *F*_st_ and IBS (see above). *P*_n_/*P*_s_ scores were calculated separately for subgroups of rye clusters representing ‘wild-like’ and ‘domesticated-like’ ryes separately (see main text). *P*_n_/*P*_s_ scores were only estimated for bins in which the combined number of rye-segregating variants exceeded nine.

### Wheat–rye introgression haplotype identification and classification

We assayed for the presence of 1R germplasm in wheat genotypes in silico by mapping various wheat sequence data to a combined reference genome made up of the pseudomolecules of rye line ‘Lo7’ (this study) and wheat cv. ‘Chinese Spring’^7^. Publicly available data were obtained from WHEALBI project resources^68^ (*n* = 506), CIMMYT (*n* = 903) and KSU (*n* = 4,277). GBS libraries were constructed and sequenced for samples from USDA-RPN (*n* = 875; Supplementary Table 23) as described in Rife et al.^69^. On the basis of the approach described by Keilwagen et al.^70^, reads were demultiplexed with a custom C script (github.com/umngao/splitgbs) and aligned to the combined reference using bwa mem (v.0.7, arguments -M)^71^ after trimming adapters with cutadapt^72^. The aligned reads from all panels were filtered for quality using samtools^73^ (v.1.9, arguments -F3332 -q20). The numbers of reads aligned to 1 Mb nonoverlapping bins on each pseudomolecule were tabulated. The counts were expressed as rpmm ≡ log_2_(reads mapped to bin per million reads mapped*)*. To control for mappability biases over the genome, the rpmm for each bin was normalized by subtracting the rpmm attained by the ‘Chinese Spring’ sample for the same bin to give the normalized rpmm, *r*.

To investigate the possibility of classifying the samples automatically, visual representations of *r* across the combined reference genome were inspected and obvious cases of 1R.1A and 1R.1B introgression were distinguished from several other karyotypes, including nonintrogressed samples and ambiguous samples showing a slight overabundance of 1RS reads but less discernible signals of depletion in 1A or 1B (Supplementary Note). We defined the following features for each sample: *featureA* = –log[(mean(*r*^1A^_*I*_) – mean(*r*^1A^_*N*_)) × (mean(*r*^1R^_*I*_) – mean(*r*^1^^R^_*N*_))] and *featureB* = –log[(mean(*r*^1B^_*I*_) – mean(*r*^1B^_*N*_)) × (mean(*r*^1R^_*I*_) – mean(*r*^1R^_*N*_))]. Whenever the term inside the log was negative (and would thus give an undefined result), the value of the feature was set to the minimum of the defined values for that feature. The quantity mean(*r*^1R^_*I*_*)* refers to the average value of *r* for all bins within the terminal 200 Mb of the normally introgressed (*I*) end of 1R (an *N* in the subscript denotes the terminal 300 Mb of the normally nonintrogressed (*N*) arm) and so forth for other chromosomes. This choice of feature definition meant that, wherever little difference in *r* occurred between 1RS and 1RL, suggesting no presence of rye, the factor mean(*r*^1R^_*I*_) *–* mean(*r*^1^^R^_*N*_) would pull the feature values close to the origin and differences between *r* on the long and short arms of 1A or 1B would pull the values of A or B respectively away from the origin, depending upon which introgressions are present. A classifier was developed by training an SVM to distinguish nonintrogressed, 1A.1R-introgressed, 1B.1R-introgressed and ambiguously introgressed samples, using the function ksvm (arguments Type=’C-svc’, kernel=‘rbfdot’, C=1) from the R package kernlab. Classification results are given in Supplementary Table 25. Testing was performed by generating sets of between 50 and 600 random samples from the dataset and using these to train a model, then using the kernlab::predict() to test the model’s accuracy of prediction on the remaining data not used in training. This was repeated 100 times for each training dataset size.

To confirm the common origin of the 1AL.RS and 1BL.1RS introgressions, predicted 1RS carriers were selected to form a combined 1RS panel (over 1,200 lines) to call SNPs. A total of over 3 million SNPs were called with samtools/bcftools v.1.9 (mpileup -q20, -r chr1R:1-300000000; call -mv). SNPs were filtered on the basis of combined minimum read depth of 25, minor allele frequency of 0.01. A total of >900,000 SNPs were obtained. All IBS percentages were calculated and the square root values of per cent different calls were used to derive a heatmap for all pairwise comparisons (Supplementary Note).

### SV detection in ‘Puma-SK’ and ‘NorstarPuma5A:5R’

To characterize the *Fr2* region in ‘Puma-SK’ and the introgression in ‘NorstarPuma5A:5R’, whole-genome sequencing was performed using the Chromium 10x Genomics platform. Nuclei were isolated from 30 seedlings and high molecular-weight genomic DNA was extracted from nuclei using phenol chloroform according to the protocol of Zheng et al.^74^. Genomic DNA was quantified by fluorometry using Qubit 2.0 Broad Range (Thermofisher) and size selection was performed to remove fragments smaller than 40 kb using pulsed field electrophoresis on a Blue Pippin (Sage Science) according to the manufacturer’s specifications. Integrity and size of the size-selected DNA were determined using a Tapestation 2200 (Agilent) and Qubit 2.0 Broad Range (Thermofisher), respectively. Library preparation was performed as per the 10x Genomics Genome Library protocol (https://support.10xgenomics.com/genome-exome/library-prep/doc/user-guide-chromium-genome-reagent-kit-v2-chemistry) and uniquely barcoded libraries were prepared and multiplexed for sequencing by Illumina HiSeq. Demultiplexing and the generation of fastq files were performed using LongRanger v.2.2.0 mkfastq (https://support.10xgenomics.com/genome-exome/software/pipelines/latest/using/mkfastq; default parameters).

Sequencing reads from ‘Puma-SK’ and ‘NorstarPuma5A:5R’ were aligned to the rye line ‘Lo7’ and bread wheat cv. ‘Chinese Spring’^7^ genome assemblies, respectively, using LongRanger WGS (https://support.10xgenomics.com/genome-exome/software/pipelines/latest/using/wgs; arguments -vcmode ‘freebayes’). Large-scale structural variants detected by LongRanger were visualized with a combination of Loupe (v.2.1.1; https://support.10xgenomics.com/genome-exome/software/visualization/latest/what-is-loupe; downloaded February 2019; Supplementary Table 33). Short variants were called using the Freebayes software (github.com/ekg/freebayes) implemented within the Longranger v.2.2.0 WGS pipeline. For determining the introgression, ‘NorstarPuma5A5R’ reads which did not map to the ‘Chinese Spring’ reference were aligned to the ‘Lo7’ assembly using the LongRanger align pipeline (https://support.10xgenomics.com/genome-exome/software/pipelines/latest/advanced/other-pipelines). Samtools (v.1.9)^73^ bedcov was used to calculate the genome-wide read coverage across both references. CNV between ‘Puma-SK’ and ‘Lo7’ was detected using a combination of barcode coverage analysis output by the Longranger WGS pipeline and read depth-of-coverage based analysis using CNVnator^75^ v.0.4 and cn.mops^76^ v.1.12.0.

### Expression profiling of ‘NorstarPuma5A:5R’ and ‘Puma’

RNA from ‘NorstarPuma5A:5R’ and ‘Puma’ was isolated and sequenced as described above. Sequencing adapters were removed and low-quality reads were trimmed using Trimmomatic^77^. RNA reads from ‘NorstarPuma5A:5R’ and ‘Puma’ were aligned to the ‘Lo7’ reference using Hisat2 (ref. ^78^; v.2.1.0; default arguments) and transcripts were quantified with htseq (ref. ^79^; v.11.1; default parameters). Differential expression analysis was carried out using DESeq2 (ref. ^80^; v.3.11; default parameters).

### Reporting Summary

Further information on research design is available in the Nature Research Reporting Summary linked to this article.

## Online content

Any methods, additional references, Nature Research reporting summaries, source data, extended data, supplementary information, acknowledgements, peer review information; details of author contributions and competing interests; and statements of data and code availability are available at 10.1038/s41588-021-00807-0.

## Supplementary information

Supplementary Information Supplementary Note

Reporting Summary

Supplementary Tables Supplementary Tables 1–34

## Data Availability

The ‘Lo7’ assembly and gene feature annotation data are available via e!DAL at 10.5447/ipk/2020/33 and 10.5447/ipk/2020/29. The visual suite of resources for assembly assessment are stored at 10.5447/ipk/2020/32. Raw sequence data generated in the course of the study are available at European Nucleotide Archive (ENA) with accession numbers PRJEB32636 (PE and MP data for assembly), PRJEB32574 and PRJEB34626 (Hi-C), PRJEB34439 (10x), PRJEB32587 (CSS), PRJEB35392 (GBS data) and PRJEB35461 (RNAseq and IsoSeq for annotation of ‘Lo7’). Chromium 10x and RNAseq data for ‘Puma’ and ‘Norstar’ are available at PRJNA564622. The SNP matrix used for rye population genetic analyses is available via e!DAL at 10.5447/ipk/2020/31. GBS and sequence data generated for the USDA and CIMMYT wheat diversity panels are available at ENA with accession numbers PRJNA566410, PRJNA566408 and PRJNA566409. Optical map data and alignments are available via e!DAL at 10.5447/ipk/2020/30. High-stringency transposable element annotations (used for evolutionary analyses) are given in Supplementary Table 10, while the larger, low-stringency annotations (used for assembly quality comparisons) are available via e!DAL at 10.5447/ipk/2020/34.

## Code availability

The custom miRNA manipulation scripts used in miRNA annotation (SumirFind.pl, SumirFold.pl, SumirLocate_v2.py and Sumircreen_v2.py) are available at https://github.com/hikmetbudak/miRNA-annotation. Two custom scripts used for parsing BLAST output (get_alleles_at_position.c and blast_to_snps.c) are available at https://github.com/mtrw/tim_bioinfo_tools and custom R functions extending or modifying functions of the TRITEX assembly pipeline (version corresponding to commit ID 2898e74) are available at https://github.com/mtrw/Sc_genome_assembly. The custom tool used to demultiplex wheat panel GBS data (splitgbs.c) is available at github.com/umngao/splitgbs.
