# Supplementary material for: Chromosome-scale genome assembly provides insights into rye biology, evolution and agronomic potential
Source: Nat Genet. 2021 Mar 18;53(4):564–73. doi: 10.1038/s41588-021-00807-0 (PMC8035072; doi:10.1038/s41588-021-00807-0)
Supplement: Supplementary file 1 — Supplementary Note [file 41588_2021_807_MOESM1_ESM.pdf]

nature portfolio

https://doi.org/10.1038/s41588-021-00807-0

Supplementary information

Chromosome-scale genome assembly provides insights into rye biology, evolution and agronomic potential

In the format provided by the authors and unedited

Supplementary Materials

IRGSC Rye Genome. Rabanus-Wallace et al.

Supplementary Note: Data generation, primary processing, and quality control 4
1.1. Paired-end and mate paired read sequencing for assembly; alignment to assembly scaffolds 4
Supplementary Note Figure 1.1. 5
1.2. Hi-C sequencing for assembly and Hi-C–based analyses; alignment to assembly scaffolds 6
Supplementary Note Figure 1.2. 7
1.3. 10X Chromium linked read sequencing for assembly; alignment to assembly scaffolds 8
Supplementary Note Figure 1.3. 8
Supplementary Note Figure 1.4. 9
Supplementary Note Figure 1.5. 9
1.4. Chromosome Sorted Shotgun (CSS) sequence for assembly; alignment to assembly scaffolds 10
1.5. Bionano optical map generation for assembly; alignment to assembly scaffolds 10
1.6. RNASeq and IsoSeq data for gene feature annotation 11
Supplementary Note Figure 1.6. 13
Supplementary Note: Pseudomolecule assembly procedure 14
2.1 Overview 14
Supplementary Note Figure 2.1. 15
2.2. Contiging and Scaffolding (step 1) 16
2.3. Scaffold chromosome assignments using genetic map, CSS, and Hi-C .. 16
2.5. Guide Hi-C map construction (steps 3, 5, and 7) 18
2.6. Comments on manual AGP editing using the visualisation suite (steps 4, 6, and 8) 18
2.7. Manual chimera detection and breaking (step 4) 19
2.8. Superscaffolding (step 6) 20
2.9. Final order adjustment (step 8) 20
Supplementary Note Figure 2.2. 20
Supplementary Note Figure 2.3. 22
Supplementary Note Figure 2.4. 22
Supplementary Note Figure 2.5. 23
Supplementary Note Figure 2.6. 23
Supplementary Note Figure 2.7. 24
Supplementary Note: Genome Annotation 25
3.1 Gene annotation 25
3.2 Annotation of repetitive elements 26
3.3 Annotation of miRNAs 28
3.4 Identification and analysis of gene families 28
Supplementary Note: Investigations into the repetitive genome 30

Supplementary Note Figure 4.1. 31
Supplementary Note Figure 4.2. 32
Supplementary Note Figure 4.3. 33
Supplementary Note Figure 4.4. 35
Supplementary Note Figure 4.5. 36
Supplementary Note Figure 4.6. 37
Supplementary Note Figure 4.7. 38
Supplementary Note: Detailed gene-level collinearity analysis of rye, barley, and wheat 39
Supplementary Note Figure 5.1. 40
Supplementary Note Figure 5.2 41
Supplementary Note Figure 5.3. 42
Supplementary Note Figure 5.4. 43
Supplementary Note Figure 5.5. 44
Supplementary Note Figure 5.6. 45
Supplementary Note Figure 5.7. 46
Supplementary Note: *Secale* diversity and segregating structural variations............47
Supplementary Note Figure 6.1. 47
Supplementary Note Figure 6.2. 48
Supplementary Note: Investigations on the evolution of the Triticeae focussing on rye49
Supplementary Note Figure 7.1. 49
Supplementary Note Figure 7.2. 50
Supplementary Note Figure 7.3. 51
Supplementary Note Figure 7.4. 52
Supplementary Note: Rye-wheat introgression lines classified using high-throughput shotgun sequencing 56
Supplementary Note Figure 8.1. 57
Supplementary Note Figure 8.2. 58
Supplementary Note: Evidence from large-scale studies relating to the yield benefits of wheat-rye translocations..59
Supplementary Note Figure 9.1. 60
Supplementary Note 10: Comparative cluster arrangement and phylogenetics of genes with agricultural importance..61
10.1 Phylogenetic and homology-based investigations of resistance gene orthologs 61
Supplementary Note Figure 10.1. 63
Supplementary Note Figure 10.2. 64
Supplementary Note Figure 10.3. 65
Supplementary Note Figure 10.4. 66
Supplementary Note Figure 10.5. 67
Supplementary Note Figure 10.6. 68
Supplementary Note Figure 10.7. 69
Supplementary Note Figure 10.8. 70
Supplementary Note Figure 10.9. 71
10.2 Detailed dissection of the Rf^{multi} locus in rye.. 72
Supplementary Note Figure 10.10. 72

10.3 Phylogenetic and homology-based investigations of low temperature tolerance gene homologs... 73
Supplementary Note Figure 10.11. 73
Supplementary Note References 74

Supplementary Note: Data generation, primary processing, and quality control

We describe here the generation and primary processing of sequence datasets, and other datasets integral to the genome assembly and annotation. Tabulated descriptive statistics and quality control information for data used in the 'Lo7' assembly are given in Supplementary Tables 3—7, and Supplementary Note Figures 1.1—1.5. Access information for all data is given in the Data Accessibility Statement.

Much of the data processing required for genome assembly required the alignment of sequencing reads to the raw scaffold set, whose generation using the proprietary DeNovoMAGIC3.0 pipeline by NRGene Israel is described in the subsequent note on genome assembly. These pre-assembly procedures made heavy use of scripts from the TRITEX[1] assembly pipeline (https://bitbucket.org/tritexassembly; version corresponding to commit ID 2898e74).

Steps performed in the R statistical environment were performed with R version 3.4.2, with all packages and their dependencies updated to their most current versions on the 1st of February 2018.

1.1. Paired-end and mate paired read sequencing for assembly; alignment to assembly scaffolds

DNA was isolated at IPK Gatersleben from fresh leaf tissue of *Secale cereale* inbred line 'Lo7' provided by KWS Lochow GMBH using the high molecular weight (HMW) phenol-chloroform protocol described by Dvorak et al. (1988)[2]. The DNA was provided to NRGene Israel for sequencing library construction using standard Illumina protocols. The TruSeq protocol for PCR-free shotgun paired end reads (https://www.illumina.com/documents/products/datasheets/datasheet_truseq_dna_pcr_free_sample_prep.pdf) was used to produce four paired end libraries with two distinct insert size ranges (Supplementary Table 3). The Nextera Gel-Plus protocol (https://www.illumina.com/content/dam/illumina-marketing/documents/products/datasheets/datasheet_nextera_mate_pair.pdf) was used to produce six mate paired libraries with three insert size ranges (Supplementary Table 3). All libraries were sequenced on Illumina platforms with the aim to achieving between 36-fold and 88-fold approximate coverage of an estimated 7.9 Gbp genome (Supplementary Table 3). These data, alongside the 10X Chromium data (see entry 1.2 of this Supplementary Note), was supplied to NRGene's DeNovoMagic3.0
pipeline in order to assemble the raw scaffold set (see subsequent Supplementary Note). To assess the proportion of sequence in the reads present in the raw scaffold set, the paired end reads were mapped to the scaffolds using minimap2 (v2.1; -x 'sr' presets), filtered/counted with samtools view (v1.9; flags -F 772 and -F 772 -f 2; Supplementary Table 8), and the distribution

of depths over the scaffolds (excluding no-call ‘N’ nucleotides) was calculated using samtools depth (v1.9, all default settings; Supplementary Note Figure 1.1).

Supplementary Note Figure 1.1.

Short read coverage of the raw scaffolds, ascertained by mapping ~947 Gb of short read data. Regions of no call (‘N’) nucleotides are excluded. Blue=Pseudomolecule assembly; Red=Pseudomolecules + unplaced scaffolds (*“chrUn”*). Upper panel = full distribution, with vertical bars showing the mean and increments of 1 standard deviation. Lower panel = distribution zoomed view to show peak. Approximately 96.4% of the pseudomolecule assembly falls within the depth range delineated by dashed lines.

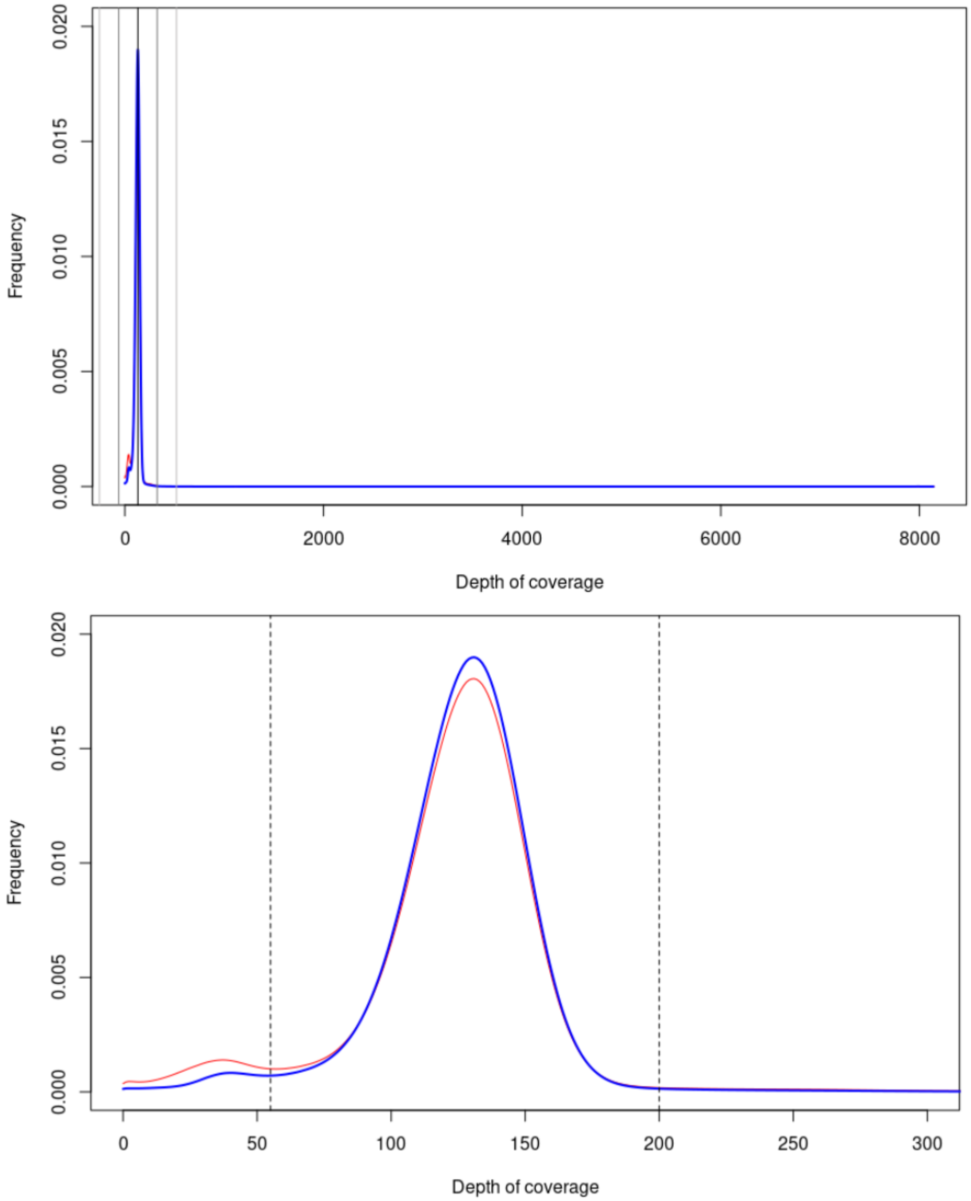

1.2. Hi-C sequencing for assembly and Hi-C–based analyses; alignment to assembly scaffolds

Leaf material (~1.5 g) was collected from one-week-old seedlings of lines ‘Lo7’ and ‘Lo225’ (accessions provided by KWS Lochow GMBH), and three wild rye gene bank accessions from the IPK Gatersleben, including *Secale vavilovii* (GBIS ID: R 1003), *Secale strictum* (R 2446), and *Secale sylvestre* (R 925). The ‘Lo7’ libraries used in pseudomolecule construction were prepared using the Tethered Conformation Capture (TCC) protocol detailed in Himmelbach et al. (2018)[3], digesting with the restriction enzyme *Hind*III (recognition site AAGCTT). All samples were subsequently used to construct Hi-C libraries using the *DpnII*-restricted in situ Hi-C method described in Padmarasu et al. (2019)[4]. Hi-C libraries were size selected on SYBR Gold-stained gels to isolate fragments in the size range of 250—500 bp. The presence of the desired construct was assayed by digesting the library with *Cla*I and visually observing the shift in size profile to low molecular weight fragments compared to undigested control library. The libraries were sequenced on one lane of the HiSeq 2500 platform in high output mode (2 x 100 bp). The libraries were trimmed at the appropriate Hi-C linker sequence (*Hind*III: AAGCTAGCTT; *Dpn*II: GATCGATC) using bbduk (v37.28, https://github.com/BioInfoTools/BBMap; options ktrim=r, k=8, mink=2, qtrim=rl, trimq=20).

The *Hind*III-digested TCC library used for pseudomolecule construction was mapped to the NRGene scaffolds (see methods in subsequent Supplementary Note), allowing an *in silico* digestion of the scaffolds with the *Hind*III recognition sequence and subsequent identification of read pairs representing valid Hi-C links between restriction fragments, as described in Beier et al. (2017)[5], implemented using the TRITEX[1] pipeline script run_hic_mapping.zsh. The script marks validly-mapped read pairs that do not show an arrangement suggesting they originated from the linking of restriction fragments as *“paired end”* reads during this step (Supplementary Table 5; Supplementary Note Figure 1.2). These are treated as contaminants and removed.

Mapping and valid pair identification were performed identically for the *Dpn*II-digested in situ Hi-C libraries used to detect SVs, except these were mapped directly to the pseudomolecule sequences (see methods in subsequent Supplementary Note), then *in silico* digested with the *Dpn*II recognition sequence (Supplementary Table 5; Supplementary Note Figure 1.2).

Supplementary Note Figure 1.2.

Fragment length histograms for Hi-C libraries, shown for both valid links and paired-end read contamination. Hi-C libraries for all taxa were constructed using the *Dpn*II enzyme, except the 'Lo7' library used in assembly, which was constructed with *Hind*III (as indicated in the facet label). Red=Valid Hi-C links. Blue=Invalid mapped pairs.

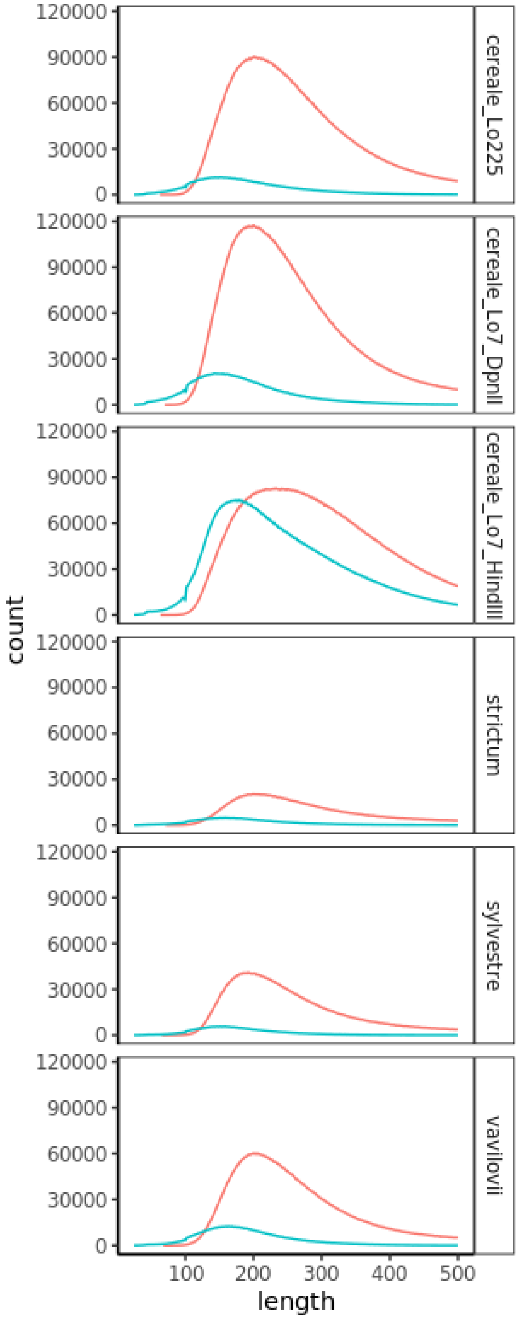

1.3. 10X Chromium linked read sequencing for assembly; alignment to assembly scaffolds

The HMW 'Lo7' DNA used for PE/MP sequencing was quantified by fluorometry using Qubit 2.0 Broad Range (Thermofisher) and size selection was performed to remove fragments smaller than 40 kb using pulsed field electrophoresis on a Blue Pippin (Sage Science) according to the manufacturer's specifications. Final DNA integrity and size were determined using a Tapestation 2200 (Agilent), and Qubit 2.0 Broad Range (Thermofisher), respectively. Library preparation was performed as per the 10X Genomics Genome Library protocol (10X Genomics) and uniquely barcoded libraries were prepared and multiplexed for Illumina sequencing. Three lanes of data were produced on the HiSeq2500 (paired end 150 bp), and an additional lane on the HiSeqX (paired end 150 bp; Supplementary Table 4). Read quality processing, mapping of reads to the NRGene 'Lo7' scaffold set (see subsequent Supplementary Note), and molecule position/length/coverage calculations were all performed using the 10X processing functions of the TRITEX pipeline[6] (run_10x_mapping.zsh; Supplementary Note Figures 1.3—1.5).

Supplementary Note Figure 1.3.

Frequency histogram over the numbers of read pairs representing an inferred molecule in 10X Genomics Chromium libraries mapped to the NRGene 'Lo7' assembly scaffolds. Read pair counts are binned into groups of 5.

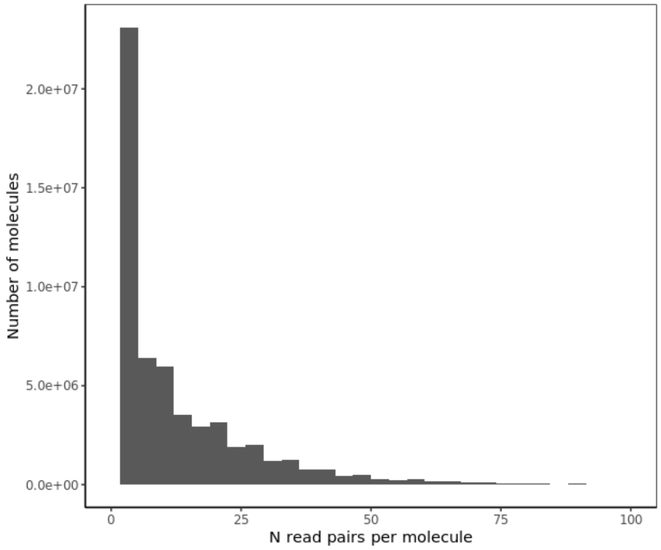

Supplementary Note Figure 1.4.

Inferred frequencies of molecule lengths in 10X Genomics Chromium libraries mapped to the NRGene ‘Lo7’ assembly scaffolds. Molecule lengths binned into groups of 20,000.

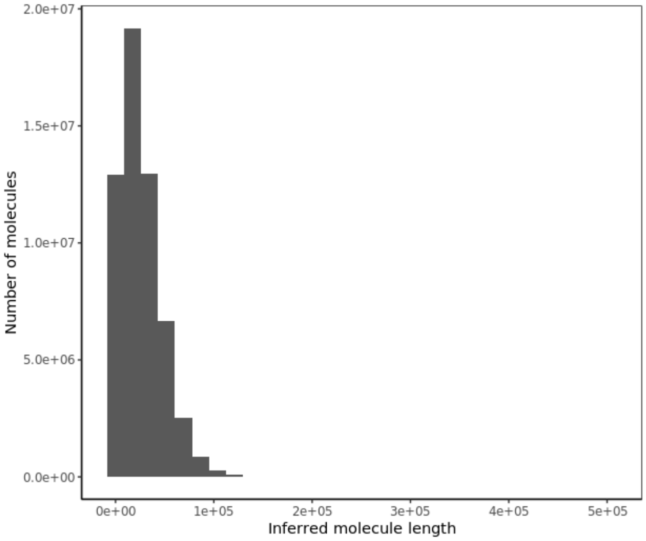

Supplementary Note Figure 1.5.

Inferred 10X Genomics Chromium molecule coverage (molecules spanning 1000 bp bins) across all NRGene ‘Lo7’ assembly scaffolds, following scaffold chimera detection and breaking (see subsequent Supplementary Note entry 2.4).

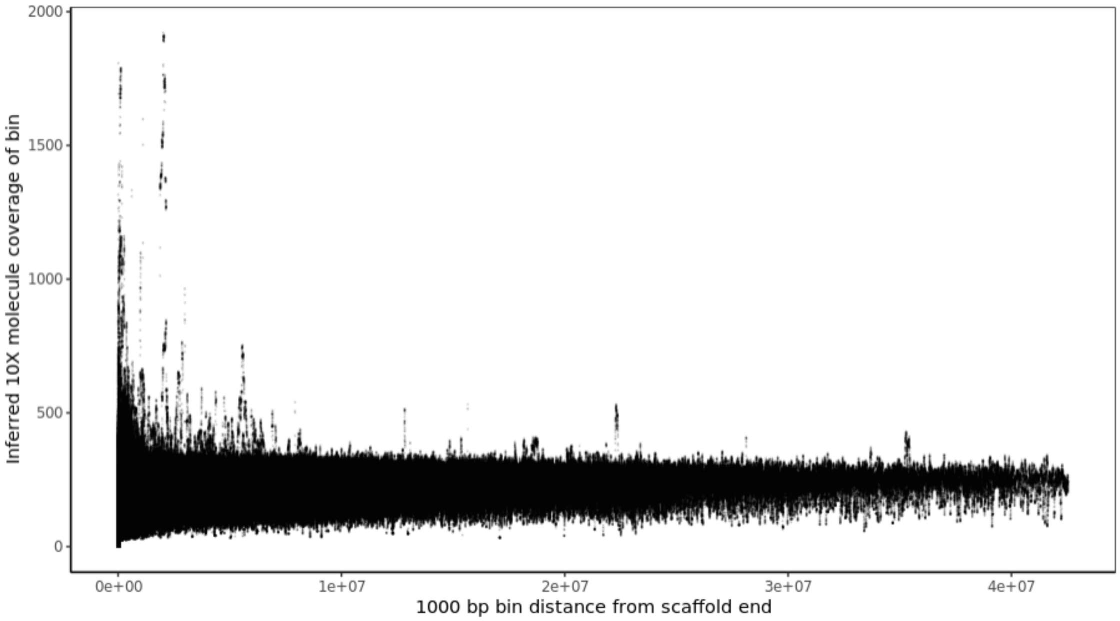

1.4. Chromosome Sorted Shotgun (CSS) sequence for assembly; alignment to assembly scaffolds

DNA from ‘Lo7’ was isolated from preparations of individual rye chromosomes by chromosome flow-sorting as described in Online Methods[7], and 500 bp insert Illumina sequencing libraries from each were prepared for sequencing on the GAIIx platform (paired end 2 x 150 bp; Supplementary Table 6) according to the manufacturer’s protocol. Adapter contamination was removed using cutadapt[8] (v1.9.1; flags -a AGATCGGAAGAGC -A AGATCGGAAGAGC -m 30), and mapped to the raw ‘Lo7’ assembly scaffolds (see subsequent Supplementary Note) using minimap2[9] (v2.1; default parameters, short read algorithm -x ‘sr’; Supplementary Table 6).

1.5. Bionano optical map generation for assembly; alignment to assembly scaffolds

Long-range scaffolding of the genome sequence was supported by an optical map constructed from the ‘Lo7’ inbred line. HMW DNA was prepared from 10.5 million mitotic chromosomes (~22 µg DNA) purified by flow cytometry according to the methods of Kubaláková et al. (2003)[7] and Šimková et al. (2003)[10]. The HMW DNA was labelled using NLRS DNA Labeling Kit (Bionano Genomics) at *Nt.Bsp*QI sites (GCTCTTC motif) and analyzed on the Irys platform (Bionano Genomics). A total of 2570.5 Gbp single molecule data > 150 kbp, corresponding to 347 ‘Lo7’ genome equivalents, was collected from nine Irys chips. The single molecule data were used to de novo assemble a map of the rye genome ‘Lo7’. A total of 2.57 Tbp of single molecule data with a length N50 of 217 kbp was used in a de novo assembly using Bionano Solve 3.1. Standard parameters for Irys data were used without “extend and split” and without haplotype refinement in order to create a single map for each allele with the parameter file “optArguments_nonhaplotype_noES_irys.xml.” In brief, the *de novo* assembly was accomplished by generating an assembly graph from alignments, satisfying a p-value threshold of 1e-10, finding during a pairwise comparison of all of the molecules, followed by refinement of label positions and extension of the ends of the maps based on molecules aligned to the maps with a p-value threshold of 1e-11. After five rounds of extension and refinement, a final refinement was conducted with a p-value threshold of 1e-15, a *de novo* map was produced with a total length of 6,660 Gbp and a map N50 of 1.671 Mbp (Supplementary Table 7). The NRGene ‘Lo7’ scaffold set (see subsequent Supplementary Note) was *in silico* digested based on the recognition sequence for Nt.BspQI. The pattern of motif sites on the *in silico* map was anchored to Bionano maps using Bionano Solve 3.1, imposing a p-value mapping threshold of 1e-10. Chimera detection (see entry 2.6 of the subsequent Supplementary Note) was accomplished by aligning contig maps to Bionano maps with a p-value threshold of 1e-13 and using the Solve find divergence feature.

1.6. RNASeq and IsoSeq data for gene feature annotation

Seeds of ‘Lo7’ were sown in a Petri dish on moistened filter paper and treated with cold stratification (4 °C) for two days during imbibition. After an additional day at room temperature (~20 °C) seedlings were transferred to a 40-well tray containing a peat and sand compost and propagated in a Conviron BDW80 cold environment room (CER; Conviron) with set points of 16 h day/8 h night and temperatures of 20/16 °C for a further three days. Tissues were sampled at six stages, as follows:

Timepoint	Organ	Stage	Zadock's GS	Days to Sampling
1	Whole root	3-leaf stage	GS13	22
2	Whole aerial organs	3-leaf stage	GS13	22
3	Whole aerial organs	3-leaf stage *2 hours after dusk*	GS13	22
4	Complete spike	complete heading	GS59	114
5	Flag leaf	7 days post anthesis	GS71	125
6	Whole grains	15 days post anthesis	GS77	133

Plants for sampling timepoints 1—3 were transferred to a CER set at 16-hour photoperiod (300 μmol m^{-2} s^{-1}), temperatures of 20 and 16 °C, respectively, and 60% relative humidity. Plants for sampling timepoints 4—6 were transferred to a vernalisation CER running at 6 °C with 8 hours photoperiod for 61 days. After this period the plants were transferred to 1 L pots containing Petersfield Cereal Mix (Petersfield, Leicester, UK) and moved to the CER with settings as described above. Total RNA was extracted from each of the six organ/stages using RNeasy plant mini-kits (Qiagen). For the RNAseq data sets used for the annotation. RNA from 3 biological replicates for each organ/stage was pooled and for the 6 pooled samples, library construction and sequencing on the Illumina NovaSeq platform was performed by Novogene using a standard strand-specific protocol (en.novogene.com/next-generation-sequencing-services/gene-regulation/mrna-sequencing-service) and generating >60 M 150 PE reads per sample.

For the IsoSeq data used in the annotation RNA from root and shoot samples were used (timepoints 1 and 2 in the table above). The IsoSeq libraries were created starting from 1µg of total RNA per sample and full-length cDNA was then generated using the SMARTer PCR cDNA synthesis kit (Clontech) following PacBio recommendations set out in the IsoSeq method (pacb.com/wp-content/uploads/Procedure-Checklist-Iso-Seq-Template-Preparation-for-Sequel-Systems.pdf). PCR optimisation was carried out on the full-length cDNA using the KAPA HiFi PCR kit (Kapa Biosystems) and 10—12 cycles was sufficient to generate the material required for SMRTbell library preparation. The libraries were then completed following PacBio

recommendations, without gel-based size-selection (pacb.com/wp-content/uploads/Procedure-Checklist-Iso-Seq-Template-Preparation-for-Sequel-Systems.pdf).
The library was quality checked using a Qubit Fluorometer 3.0 (Invitrogen) and sized using the Bioanalyzer HS DNA chip (Agilent Technologies). The loading calculations for sequencing were completed using the PacBio SMRTlink Binding Calculator v5.1.0.26367. The sequencing primer from the SMRTbell Template Prep Kit 1.0-SPv3 was annealed to the adapter sequence of the libraries. Each library was bound to the sequencing polymerase with the Sequel Binding Kit v2.0. Calculations for primer and polymerase binding ratios were kept at default values. Sequencing Control v2.0 was spiked into each library at ~1% prior to sequencing. The libraries were prepared for sequencing using Magbead loading onto the Sequel Sequencing Plate v2.1. The libraries were sequenced on the PacBio Sequel Instrument v1, using 1 SMRTcell v2 per library. All libraries had 600-minute movies, 120 minutes of immobilisation time, and 120 minutes pre-extension time.

1.7 RNASeq for expression profiling of 'NorstarPuma5A:5R' and 'Puma'

Genotypes 'NorstarPuma5A:5R' and 'Puma' were grown for 14 days at 20 °C with a 10 hour (h) day length. Plants were then treated to decreasing temperatures and daylengths over a 70 day period, designed to mimic field conditions for winter growth habit (see Supplementary Note Figure 1.6). For each change in temperature, crowns were sampled from two independent replicate plants for each genotype. Crown tissue was sampled one hour after the lights came on in the morning to minimise circadian rhythm effects. At each change in temperature, five plants from each genotype were used to assess the rate of plant phenological development (by dissection of the plant crown to reveal shoot apex development) and to test cold hardiness during cold acclimation. Cold hardiness was determined using LT50 measurements using the procedure outlined by Fowler et al. (2016)[11]. Total RNA was extracted from 48 samples, representing both 'NorstarPuma5A:5R' and 'Puma' lines, at 12 time points during cold acclimation (see Supplementary Note Figure 1.6), using the Plant RNA Isolation Mini Kit (Agilent Technologies). The yield and RNA purity were determined spectrophotometrically with Nanodrop 1100 (Thermfisher), and the quality of the RNA was verified by Agilent 2100 Bioanalyzer (Agilent Technologies). Purified total RNA was precipitated and re-suspended in RNase-free water to a final concentration of 100 ng/µl. Libraries were constructed using the TruSeq RNA Sample Preparation Kit v2 (Illumina) with two replicates at each time point. Paired-end sequencing was conducted on the Illumina HiSeq2500, generating 101 bp reads.

Supplementary Note Figure 1.6.

Experimental design and LT50 temperatures for 'Norstar' (dark blue), **'NorstarPuma5A:5R'** (orange), **'Puma'** (light blue), **and the temperature sensitive control Winter Manitou** (dark green). Crown temperatures (black – left vertical axis) and day length (light green – right vertical axis) are indicated over the 70 day treatment (horizontal axis). Tissues isolated at each time point were used for RNA sequencing analyses (methods).

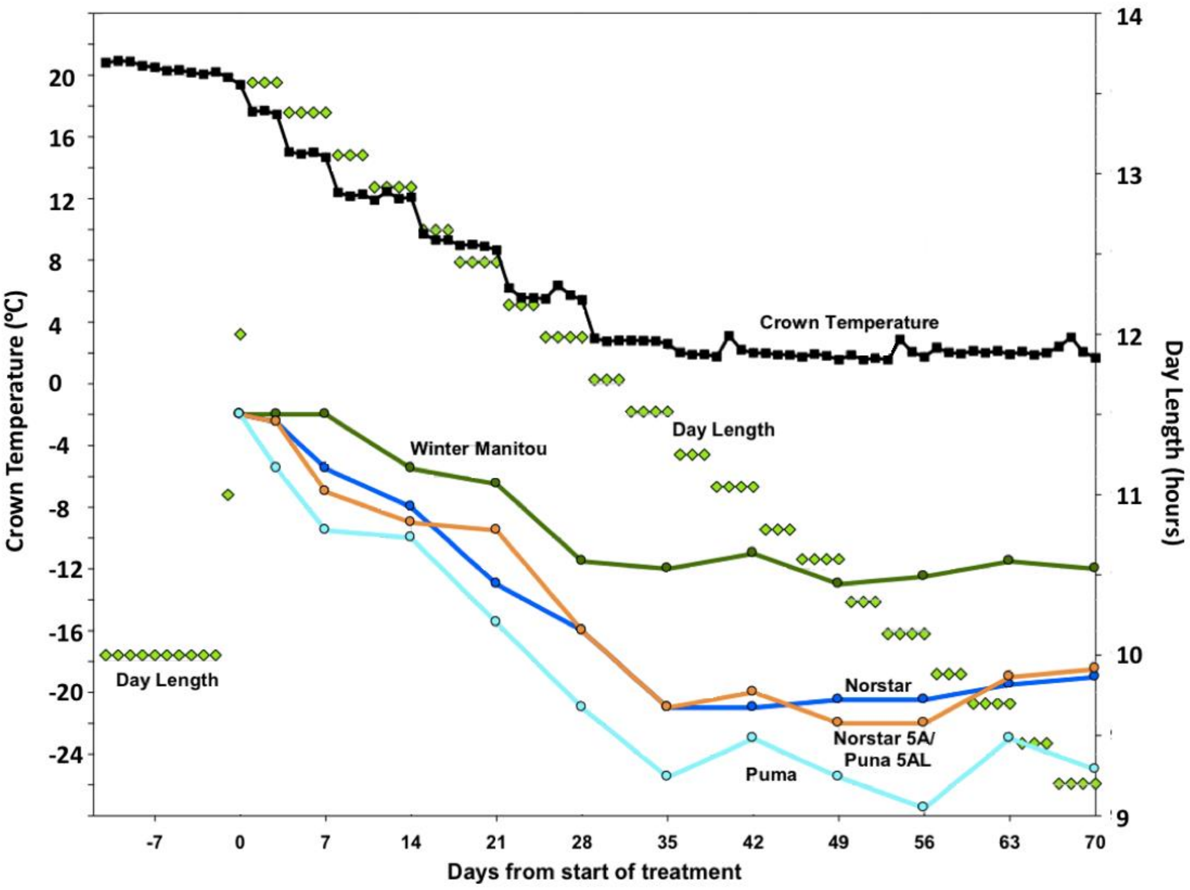

Supplementary Note: Pseudomolecule assembly procedure

2.1 Overview

The key steps of the general assembly workflow are depicted in Supplementary Note Figure 2.1. In broad terms: in step 1, raw scaffolds were assembled using NRGene's DeNovoMAGIC3.0 pipeline[12-15], using sequence data from paired-end (PE) shotgun, mate-paired (MP), and 10X chromium molecule-linked (ML) reads. Scaffolds were enriched with genetic positions and chromosome assignments by mapping the contigs of a draft rye genome assembly of Bauer et al. (2017)[16] to the new assembly scaffolds, and lifting this information from one assembly to the other. In step 2, misjoined ('chimeric') scaffolds were detected and broken using two different automatic approaches. Scaffold arrangement into pseudomolecules (steps 3 to 8) was primarily performed in the R statistical environment following the TRITEX[1] pipeline. The procedure focussed on manual curation, beginning by manually breaking any potential chimeras that were not detected automatically (step 4), then on concatenating adjacent scaffolds into superscaffolds (step 6), and finally on arranging the superscaffolds into pseudomolecules (step 8). Preceding each of these three manual steps, a candidate scaffold order was generated with the aid of Hi-C link frequency information (steps 3, 5, and 7). The candidate scaffold orders functioned each time as the basis of a suite of data (primarily Hi-C, optical map, genetic map, and CSS) visualisations designed to give the user an intuitive impression of a candidate's accuracy, at the different resolutions offered by the various datasets. Summary statistics describing the assembly at progressive points through the assembly procedure are detailed in Supplementary Table 2.

Supplementary Note Figure 2.1.

Assembly procedure overview. Coloured circles represent data sources used in each of the steps. Black=Mate paired and paired end read data; Pale blue=10X Chromium linked reads; Orange=Hi-C data ('Lo7', TCC method with HindIII digest); Dark blue=BioNano optical map alignment; Green=Genetic map positions lifted from Bauer et al. (2017)[16] assembly contigs. Yellow=CSS reads.

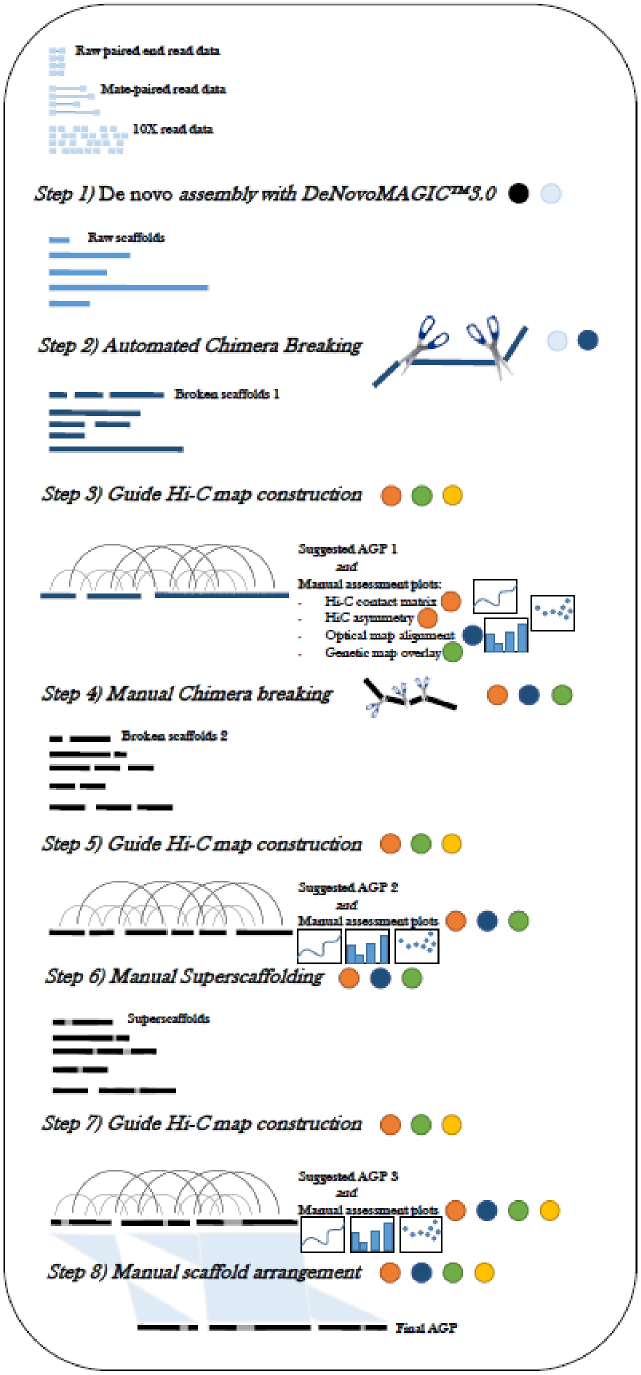

2.2. Contiging and Scaffolding (step 1)

De novo assembly was performed using NRGene's proprietary DeNovoMAGIC3.0 pipeline, as previously described[12-15]. Briefly, the procedure can be divided into the following steps: 1) Data pre-processing and quality control. Adapter sequences are trimmed and overlapping PE reads are merged. Reads containing probable sequencing errors are purged by removing any reads that contain unique or near-unique sequences; 2) Contig assembly. A 127mer De Bruijn graph assembly is constructed from the contigs present in all PE and MP reads. The PE and ML reads are then used to identify and validate likely paths through the graph; 3) Scaffolding. PE and ML reads are used to identify/validate scaffold-to-scaffold joins, and to estimate the distances between them; 4) Gap filling. The PE and MP data are employed to identify unique De Bruijn graph paths that fill intra-scaffold gaps; 5) Scaffold validation with ML reads. A window-based approach is used to determine whether the mapped positions of ML reads supports the contiguity of the scaffolds. Scaffolds are broken wherever the number of inferred molecules overlapping edges of a 20 kb window is judged inconsistent with the number spanning the whole window; 6) Scaffold concatenation with ML reads. A graph is constructed linking terminal bins containing multiple shared ML read barcodes. The scaffolds in any group forming a linear path through the graph are joined together to form a single scaffold. The assembly produced 107,580 scaffolds with N50 length >22 Mbp, and a combined length of 6,724,307,309 bp (Supplementary Table 2).

2.3. Scaffold chromosome assignments using genetic map, CSS, and Hi-C

Following the methods described in Beier et al. (2017)[5], each scaffold was also assigned to a chromosome based on the genetic map, and also given a CSS-based chromosome assignment, wherever the map markers/CSS bins associated with the scaffold showed a clear majority preference for one chromosome.

The draft 'Lo7' genome assembly of Bauer et al. (2017)[16] was co-opted as a source of mapped genetic markers. Contigs of the Bauer et al. assembly (n=1,581,707) had themselves been anchored to 87,820-markers of a high-density map constructed using the Rye600k SNP genotyping array, by genotyping a near-homozygous recombinant inbred line (RIL) population with parents 'Lo7' (seed) and 'Lo225' (pollen)[16]. These contigs were mapped to the raw scaffolds of the new assembly using minimap2[9] (v2.1, preset -x 'asm5') with preset parameters for genome-to-genome mapping. Unique, primary, non-supplementary alignments with mapping qualities exceeding 30 were retained (n=1,535,590). The genetic positions associated with 44,371 such contigs were lifted to the new assembly scaffolds, with each genetic position being linked to the middle mapping position of the mapped contig.

CSS-based chromosomal assignments of scaffold bins were made as follows: CSS read pairs (filtered using samtools10 v1.9 view, flags -q30 -F260) mapped to the 'Lo7' assembly scaffolds were counted for 100 kbp non-overlapping bins across each scaffold, and normalised to reads-per-million (rpm) for each chromosome. Each bin was assigned to the chromosome with the highest rpm. A great deal of stochasticity occurs across scaffolds, probably owing in part to contamination of chromosome preparations with small amounts of unintended chromosomes, and also to similar repetitive sequences occurring in multiple chromosomes. To help smooth out some of this stochasticity, the bin calls were smoothed using a hidden markov model (HMM), treating the bins' chromosome assignments as observed states, and the true chromosome of origin as a hidden state. The model parameters were inferred using the Baum-Welch algorithm, as implemented by the R package `mhsmm`11, training on contigs longer than 10 Mbp, with the initial emission probabilities set up with a 100 times greater probability of bin assigned to chromosome X emitting chromosome X, in order force convergence towards the correct observation/emission correspondence. Similarly, transitions were initialised with 100 times greater probabilities for states remaining the same than changing. Initial state probabilities were made equal. The model converged over 33 iterations. The Viterbi algorithm, also implemented in the R package `mhsmm`, was then used to infer the most likely bin states from the trained model.

Scaffolds for which the CSS and map assignments agreed were then used as the basis for a Hi-C-based chromosome assignment: Hi-C links between chromosome-assigned scaffolds and the focal scaffold were counted, and a Hi-C-based assignment awarded if the links overwhelmingly associated the scaffold with other scaffolds from one particular chromosome (details in Beier et al., 2017)[5].

2.4. Automated chimera detection and breaking (step 2)

The initial set of 'Lo7' assembly scaffolds ("scaffolds_v1" in Supplementary Note Figure 2.1) were broken wherever chimeric breakpoints were suspected. Two automated methods of chimera detection were used. Firstly, a group of scaffold breakpoints were suggested by the Bionano assembly software Bionano Solve v3.1, which flags conflicts between the optical map alignments (henceforth *optigs*—*op*tical con*tigs*) and the scaffolds, and a report is returned suggesting where either the scaffolds or the optigs aligned to them should be broken to resolve the conflict (see previous Supplementary Note entry 1.5). For each conflict, choice between breaking the optig or the scaffold is based on the level of support for the optig's contiguity provided by the raw optical molecule data (https://bionanogenomics.com/support-page/bionano-solve/).

At suspected breakpoints, the scaffold was split into three: two main fragments, and a small fragment of length 20 kbp surrounding the nominated breakpoint. This precaution aims to ensure the real breakpoint—which is expected to differ slightly from the nominated breakpoints owing to estimation inaccuracy—is contained in a short scaffold, and subsequently relegated to the 'unassigned' chromosome chrUn.

The second automated breakpoint detection method made use of 10X Chromium molecule-linked read data based on the expectation that the absence of molecules spanning breakpoints

should result in a sharp decrease of molecule coverage near breakpoints. The TRITEX R function break_10x() was run with the parameters ratio=-3, interval=5e4, minNbin=100, and dist=1e4. Breaks nominated by the optical map were broken with a modified version of this function, break_optigs() (https://github.com/mtrw/Sc_genome_assembly). Breaking the scaffolds at the points detected by these methods yielded the second scaffold set ("scaffolds_v2" in Supplementary Note Figure 1.1). To confirm scaffold contiguity, the molecule coverage counts (which infers the number of 10X molecules spanning 1000 bp bins across each scaffold) generated over the broken scaffolds by the TRITEX function 'add_molecule_cov()' were used to plot the ranges of coverages in bins at distances progressively further from the ends of the scaffolds (see Supplementary Note Figure 1.5).

2.5. Guide Hi-C map construction (steps 3, 5, and 7)

The first of several suggested optimal scaffold arrangements ("A golden path"; AGP) was constructed for the v2 scaffolds ("scaffolds_v2" in Supplementary Note Figure 2.1) using the Hi-C-based method described by Burton et al. (2013)[17], using the implementation described in Beier et al. (2017)[5] and available in the TRITEX pipeline[1]. Briefly, a graph structure is constructed for each chromosome, with vertices representing scaffolds, and weighted edges representing the frequency of Hi-C links between them. After removing any edges that conflict with available genetic map information, Prim's algorithm is used to yield a minimum spanning tree, the longest path through which functions as a "backbone" arrangement of putatively-ordered scaffolds. Scaffolds not included in the backbone are then inserted wherever their inclusion incurs the least additional weight. The total weight of this candidate order is then further minimised by inducing local perturbations using the K-opt and node relocation heuristics (with k=2) and accepting any improvements, as described by Wu et al. (2008)[18].

2.6. Comments on manual AGP editing using the visualisation suite (steps 4, 6, and 8)

The uses of the different data visualisations in manual scaffold arrangement editing are described in this entry. Visual examples showing components of the process are given in Supplementary Note Figures 2.3—2.6. The visualisations as produced for the final assembly are also available (see Data Accessibility Statement). The evolution of the assembly through two rounds of manual editing are visualised in Supplementary Note Figure 2.2.

The first tentative AGP produced ("AGP_v1" in Supplementary Note Figure 2.1) was used as the basis for a first suite of data visualisations for manual curation. It was noted at this stage that short scaffolds (< 500 kb in length) usually clustered into groups, which, while accounting for only a small proportion of the total assembly, had a strongly adverse effect on the contiguity of the assembly, manifesting most starkly as heavy vertical striations through the Hi-C contact

matrices. These inaccuracies could be improved by excluding short scaffolds (at the expense of assembly completeness), but this detracted from the ability to rescue some short scaffolds by manually incorporating them into superscaffolds (in step 6). It was decided therefore to include all chromosome-anchored scaffolds in the pseudomolecules until step 7.

CSS chromosome assignment plots (Supplementary Note Figure 2.3) are simply stacked histograms showing the numbers of reads from CSS libraries deriving from each chromosome that map within bins across each chromosome (see the subsequent Supplementary Note). These were used to manually identify obvious inter-chromosomal chimeras not identified in the automated chimera breaking steps (step 4).
Hi-C contact plots represent the frequency of Hi-C links between parts of the genome as a heatmap[19]. Briefly, the links between restriction fragments falling within 1 Mbp bins are counted, and a normalisation is performed to account for known biases introduced by several factors including the number of restriction fragments in the bin and the GC content surrounding these sites[20]. The counts in the resulting matrix are represented as a heatmap (see Data Accessibility Statement). These plots show discontinuities that can reflect inversions and badly-ordered scaffolds[17] though scaffold boundaries and repetitive sequence that affects the mapping rate may also affect the continuity.
Hi-C asymmetry plots[3] (Supplementary Note Figure 2.4) are derived from the same data as Hi-C contact plots. These show the ratio between the numbers of Hi-C links connecting bins to the left and right respectively of each bin along each chromosome, and are particularly useful for revealing inverted scaffolds.
The genetic positions of markers superimposed upon the AGP allows rapid detection of larger-scale inversions and misplaced scaffolds (Supplementary Note Figure 2.5), which we often found to provide information at a finer scale than was visible in the Hi-C asymmetry plots. Conversely, the Hi-C asymmetry plots easily reveal ordering errors in the pericentromeric regions where the genetic map shows little change owing to low recombination.
Alignment of the optigs to the assembly allows validation of the internal sequence accuracy of the scaffolds, and—wherever an optig contains matches to several scaffolds—assessment or revision of the suggested order and orientation (Supplementary Note Figures 2.6—2.7). The optical map can be used to join neighbouring scaffolds to create superscaffolds (see entry 2.8 of this Supplementary Note), given all other sources of data support (or at least fail to contradict) the join.

2.7. Manual chimera detection and breaking (step 4)

Sixteen chimeric scaffold breakpoints were manually detected by close inspection of the visual suite over the first suggested AGP (“AGP_v1” in Supplementary Note Figure 2.1). Where suspected breakpoints coincided with the comparatively small gaps (usually 5-20 kb) between sequential alignments to different optigs, the break was placed at the midpoint of these gaps. The scaffolds were broken with the same procedure as described for step 2, to yield the scaffolds_v3 scaffold set.

A second Hi-C guide map (AGP_2) was then constructed from the broken scaffolds (step 5).

2.8. Superscaffolding (step 6)

The visual suite was manually assessed to identify candidate scaffold joins for the creation of superscaffolds. This process primarily utilised the optical map visualisation (Supplementary Note Figures 2.6—2.7), which is generated from TRITEX assembly pipeline objects using the function `plot_optigs_agp()`; https://github.com/mtrw/Sc_genome_assembly), with each candidate being then checked against the other visualisations to assure consistency. Superscaffolds were created only where optical map contigs spanned two scaffolds and where the scaffold join A) was not contested or made ambiguous by any optig alignments, and B) did not incur any 'trade-offs' between data sets (for instance, if the optical map strongly suggested a join between two nearby scaffolds, but to do so would incur a small incongruity in the linear order of marker positions along these scaffolds, then that join was not made). Superscaffold compositions were listed manually in a table and applied to the assembly object generated by the TRITEX pipeline using the function `superscaffold()` (https://github.com/mtrw/Sc_genome_assembly). The scaffold set including superscaffolds ("scaffolds_v4" Supplementary Note Figure 2.1) was then used to produce a third Hi-C guide map, (step 7; "AGP_3" in Supplementary Note Figure 2.1). In the construction of AGP_3 the more stringent scaffold length limit was imposed as discussed entry 2.6 of this Supplementary Note, resulting in a number of short scaffolds with highly uncertain placements (and which caused notable incongruity in the Hi-C contact matrix) being relegated to the unknown chromosome.

2.9. Final order adjustment (step 8)

A series of small adjustments to AGP_3 were tried by comparing the visual suite (in particular, the Hi-C asymmetry plots and contact matrices, and the genetic map) before and after making changes, and accepting those judged to improve the assembly, and which incurred no contradictions between datasets.

Supplementary Note Figure 2.2.

Evolution of the scaffold arrangements in pseudomolecules imposed during manual editing steps. Chromosomes are arranged vertically in rows, with Mbp positions given on the horizontal-axis. Common sequences are linked between steps by green and blue blocks. The evolution of the order of sequences in each chromosome's pseudomolecule progresses from bottom to top beginning with the Hi-C-based suggested order from step 5 (lowest), to the suggested order following superscaffold construction (middle), to the final order (highest). Red triangles represent sequences lost from the pseudomolecules from one step to another (i.e.

which were either automatically assigned to chrUn owing to unresolved automated chromosome assignment, or excluded by minimum scaffold length cutoffs), and blue triangles represent sequence manually added (or re-added) to the pseudomolecules in the final step.

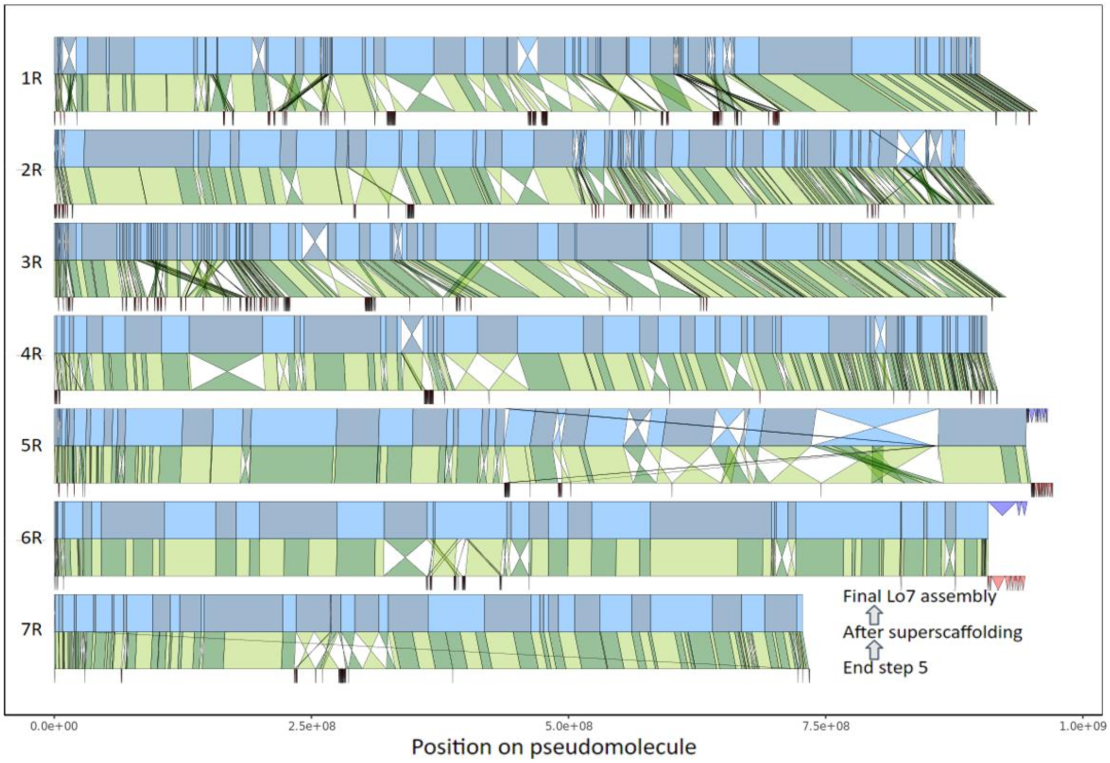

Supplementary Note Figure 2.3.

Example of an inter-chromosomal chimera, readily identifiable using CSS read depths displayed as a stacked histogram. Binned CSS read counts show that scaffold 2478 (v1 scaffolds) is a likely chimera between regions from chromosomes 3R and 5R, with the breakpoint near bin 50.

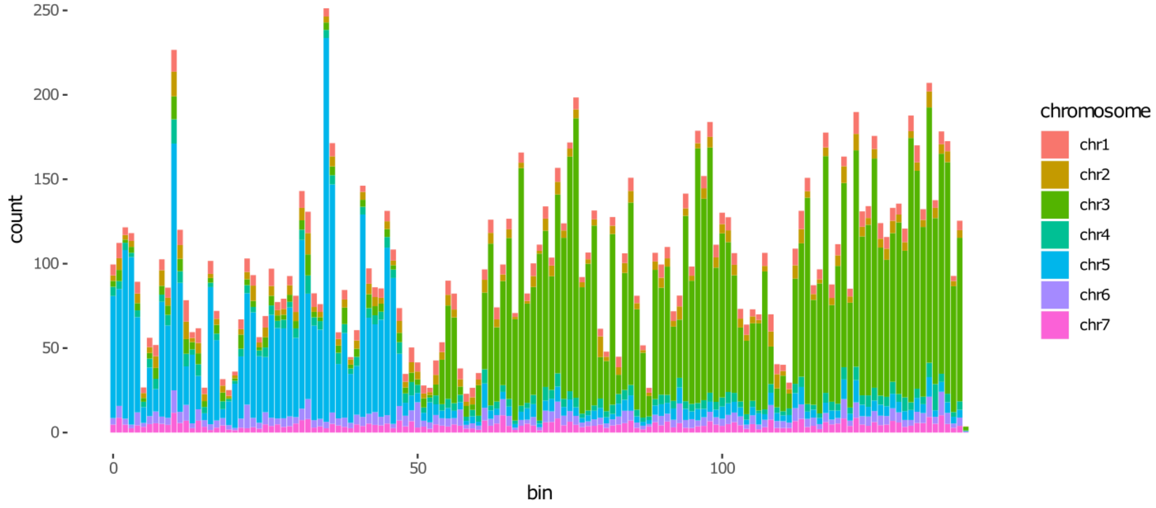

Supplementary Note Figure 2.4.

An example of Hi-C data, represented with an asymmetry plot, used as an aid for manual assembly curation. This plot is over 1 Mbp bins on chromosome 6R, showing evolution between steps 5 (top panel) and the final assembly (bottom panel). Stark diagonals and discontinuities in the step 5 asymmetry plot reveal errors in scaffold order and orientation later corrected. The discernible break in continuity in the final asymmetry plot marks the centromere. Vertical bars represent scaffold boundaries.

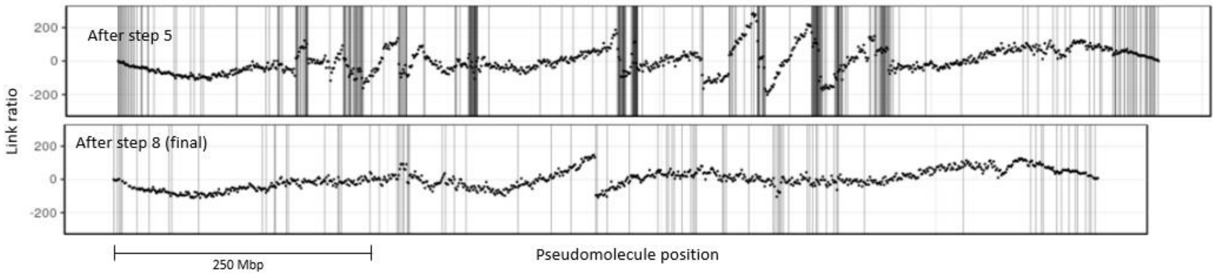

Supplementary Note Figure 2.5.

An example of genetic map marker positions used as an aid for manual assembly curation. Genetic map positions of markers (dots) superimposed onto chromosome 5R, showing evolution between steps 5 (top panel) and the final assembly (bottom panel). An inverted scaffold can be seen in the step 5 plot around 600 Mbp from the 5' end. Vertical bars represent scaffold boundaries.

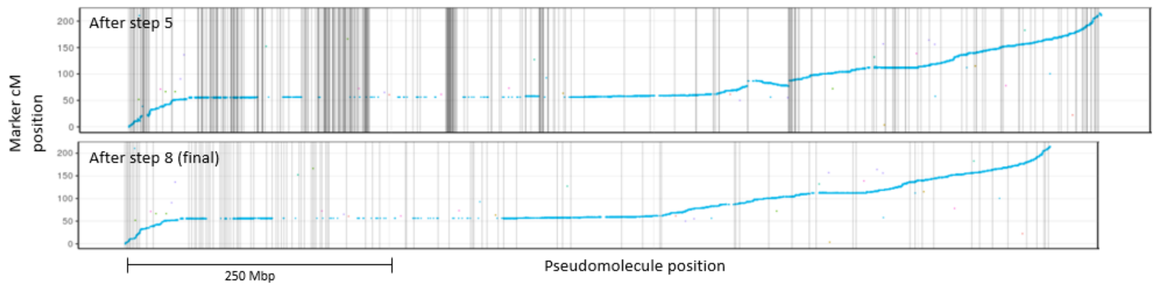

Supplementary Note Figure 2.6.

Visualisation of optical map alignments aids manual AGP editing. An excerpted portion of the optical map alignment is shown from the short arm of chromosome 1R at step 5 in the assembly process. The horizontal red bar represents scaffolds (boundaries marked with black vertical bars), arranged into putative pseudomolecules. Black horizontal bars show aligned optigs, with coloured lines linking labels to their aligned positions on the scaffolds. The directions of the optigs are arbitrary. Colours are used to distinguish distinct alignment sections. Green boxes represent contiguous scaffold groups which, as the optical map alignments suggest, could be joined to form superscaffolds. Blue arrows mark changes in scaffold orientation that the optical map alignments suggest will improve the scaffold orientation.

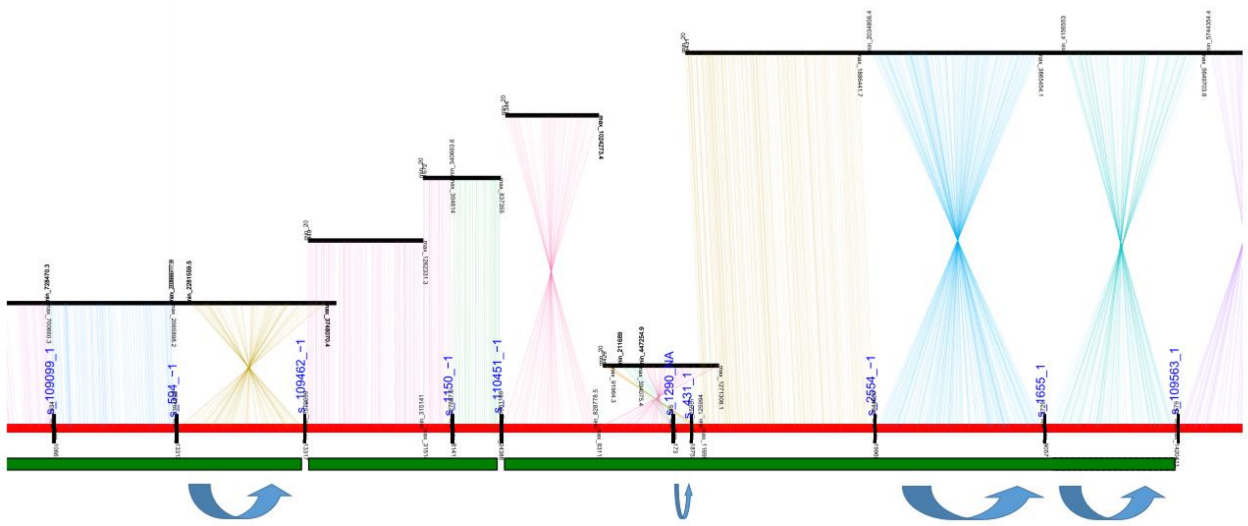

Supplementary Note Figure 2.7.

Resolution of the centromeric region of chromosome 7R (~250—300 Mbp) showing how adjustments to scaffold order improves agreement with the optical map. The optical map is displayed as described in Supplementary Note Figure 2.6 (with colours greyscale), and the changes in order displayed as in Supplementary Note Figure 2.2, with the subtending arrangement representing the result after step 5 (see Supplementary Note Figure 2.1), and the overlying arrangement representing the final assembly.

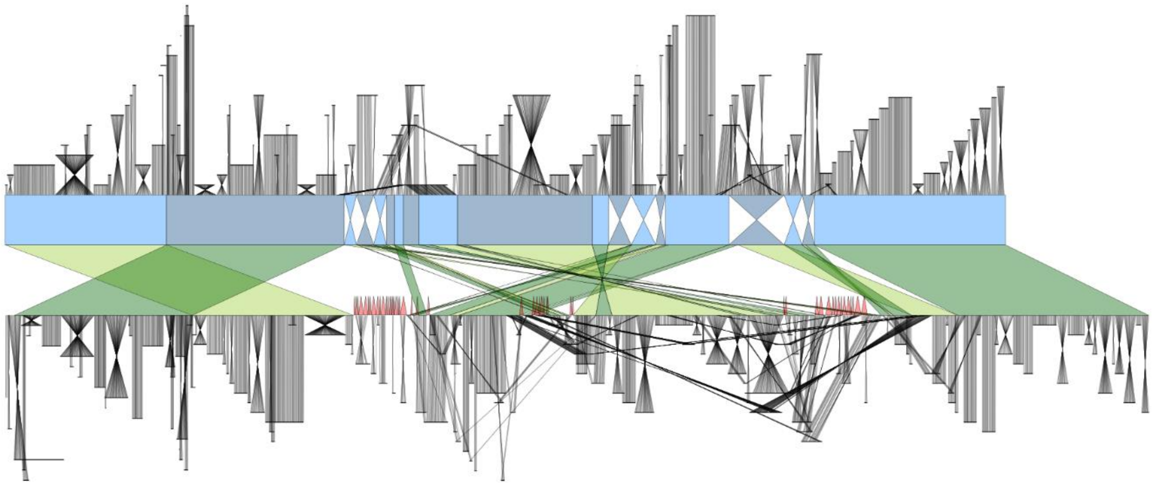

Supplementary Note: Genome Annotation

We describe here the procedures used for annotating various features in the ‘Lo7’ genome, including genes (3.1), repetitive elements (3.2), miRNAs (3.3), and specific gene families associated with pathogen resistance (3.4).

3.1 Gene annotation

We performed de novo gene annotation of the rye genome relying on a previously established automated gene prediction pipeline[21,22]. The annotation pipeline involved merging three independent annotation approaches, the first based on expression data, the second an *ab initio* prediction for structural gene annotation in plants and the third on protein homology. To aid the structural annotation, RNASeq data was derived from five different tissues/developmental stages, and IsoSeq data from three (see Supplementary Note on sequence data generation and Data Accessibility Statement).

IsoSeq nucleotide sequences were aligned to the rye pseudomolecules using GMAP[23] (v2017-01-14, default parameters), whereas RNASeq datasets were first mapped using Hisat2[24] (v2.1.0, arguments --dta) and subsequently assembled into transcript sequences by Stringtie[25] (v1.3.6, arguments -m 150 -t -f 0.3). All transcripts from IsoSeq and RNASeq were combined using Cuffcompare[26] (v2.2.1) and subsequently merged with Stringtie (arguments --merge -m 150) to remove fragments and redundant structures. Transdecoder v3.0.0 (github.com/TransDecoder) was then used to find potential open reading frames (ORFs) and to predict protein sequences. BLASTp[27] (ncbi-blast-2.3.0+, arguments -max_target_seqs 1 -evalue 1e-05) was used to compare potential protein sequences with a trusted set of reference proteins (Uniprot Magnoliophyta, reviewed/Swiss-Prot; updated 30th August 2016) and hmmscan[28] (v3.2.1) was employed to identify conserved protein family domains for all potential proteins. BLAST and hmmscan results were fed back into Transdecoder-predict to select the best translations per transcript sequence.

Homology-based annotation is based on available Triticeae protein sequences, obtained from UniProt (uniprot.org; updated 30th August 2016). Protein sequences were mapped to the nucleotide sequence of the pseudomolecules using the splice-aware alignment software GenomeThreader (v1.7.1, arguments -startcodon -finalstopcodon -species rice -gcmincoverage 70 -prseedlength 7 -prhdist 4). Evidence-based and protein homology based predictions were merged and collapsed into a non-redundant consensus gene set. *Ab initio* annotation using Augustus (v3.3.2)[29] was carried out to further improve structural gene annotation. To minimise over-prediction, hint files using IsoSeq, RNASeq, protein evidence, and TE predictions were generated. The wheat model was used for prediction.

Additionally, an independent, homology-based gene annotation was performed using GeMoMa[30] (v1.5.3) using eleven plant species: *Arabidopsis thaliana* (n=167), *Brachypodium distachyon* (314), *Glycine max* (275), *Mimulus guttatus* (256_v2.0), *Oryza sativa* (323), *Prunus persica* (298), *Populus trichocarpa* (444), *Sorghum bicolor* (454), *Setaria italica* (312), *Solanum lycopersicum* (390), and *Theobroma cacao* (233). All versions were downloaded from Phytozome (phytozome.jgi.doe.gov/pz). Initial homology search for coding exons was done with mmseqs2[31] Release 8-fac81 (https://github.com/soedinglab/MMseqs2). These results were then

combined into gene models with GeMoMa using mapped RNASeq data for splice site identification. The resulting eleven gene annotation sets were further combined and filtered using the GeMoMa module GAF. The following filters were applied: a) complete predictions (i.e. predictions starting with Methionine and ending with a stop codon); b) relative GeMoMa score >=0.75; c) evidence>1, (i.e. predictions were perfectly supported by at least two reference organisms), or tpc=1 (i.e., predictions were completely covered by RNASeq reads), or pAA>=0.7 (i.e., predictions with at least 70% positive scoring amino acid in the alignment with the reference protein).

All structural gene annotations were joined with EvidenceModeller[32] (v1.1.1), and weights were assigned as follows: Expression-based Consensus gene set (RNASeq, and IsoSeq and protein homology-based): 5; homology-based (GeMoMa), 5; ab initio (augustus), 2.

In order to differentiate candidates into complete and valid genes, non-coding transcripts, pseudogenes and transposable elements, we applied a confidence classification protocol. Candidate protein sequences were compared against the following three manually curated databases using BLAST[27] (default parameters): firstly PTREP (botserv2.uzh.ch/kelldata/trep-db; Release 19), a database of hypothetical proteins that contains deduced amino acid sequences in which, in many cases, frameshifts have been removed, which is useful for the identification of divergent TEs having no significant similarity at the DNA level; secondly UniPoa, a database comprised of annotated Poaceae proteins; thirdly UniMag, a database of validated magnoliophyta proteins. UniPoa and UniMag protein sequences were downloaded from Uniprot (www.uniprot.org/, 30th August 2016) and further filtered for complete sequences with start and stop codons. Best hits were selected for each predicted protein to each of the three databases. Only hits with an E-value below 10e-10 were considered.

Furthermore, only hits with subject coverage (for protein references) or query coverage (transposon database) above 75% were considered significant and protein sequences were further classified using the following confidence: a high confidence (HC) protein sequence is has at least one full open reading frame and has a subject and query coverage above the threshold in the UniMag database (HC1) or no BLAST hit in UniMag but in UniPoa and not TREP (HC2); a low confidence (LC) protein sequence is not complete and has a hit in the UniMag or UniPoa database but not in TREP (LC1), or no hit in UniMag and UniPoa and TREP but the protein sequence is complete.

The tag REP was assigned for protein sequences not in UniMag and complete but with hits in TREP.

Functional annotation of predicted protein sequences was done using the AHRD v1.6 pipeline (github.com/groupschoof/AHRD). Completeness of the predicted gene space was measured with BUSCO (v3; https://busco.ezlab.org/).

3.2 Annotation of repetitive elements

For use in the evolutionary analyses presented in the main text (e.g. in Main Text Figures 4d—g), we annotated a high-stringency set of full-length transposon copies belonging to single transposable element (TE) families (Supplementary Table 10) using BLASTn[27] searches (v2.3.1+, default parameters) against the ‘Lo7’ pseudomolecules for long terminal repeats (LTRs) documented in the TREP database

(botinst.uzh.ch/en/research/genetics/thomasWicker/trep-db.html). To be accepted as a genuine putative TE, the terminal repeats were required to occur at a user-defined distance range and in the same orientation: For RLC_Angela elements, the two LTRs had to be found within a range of 7,800—9,300 bp (a consensus RLC_Angela sequence has a length of approximately 8,700 bp), while a range from 6,000—12,000 bp was allowed for RLG_Sabrina and RLG_WHAM elements. For the centromere-specific RLG_Cereba elements, a narrower range of 7,600-7,900 bp was used. Multiple different LTR consensus sequences were used for the searches in order to cover the intra-family diversity. A total of 18 LTR consensus sequences each were used for RLC_Angela, seven for RLG_Sabrina elements, 6 were used for RLG_WHAM elements, and 5 for RLC_Cereba elements.
To validate the extracted TE populations, the size range of all isolated copies and the number of copies that flanked by target site duplications (TSDs) were determined. A TSD was accepted if it contained at least 3 matches between 5' and 3' TSD (e.g. ATGCG and ACGAG). This low stringency was applied because TSD generation is error-prone[33], and thus multiple mismatches can be expected. Across all surveys, 80-90% of all isolated full-length elements were flanked by a TSD.
The pipeline also extracts so-called "solo-LTRs"—products of intra-element recombination that results in loss of the internal domain and generation of a chimeric solo-LTR sequence—as a metric of how short repetitive sequences are assembled.
The two LTRs of each TE copies were aligned with the program Water from the EMBOSS package[34] (v6.6) and nucleotide differences between LTRs were used to estimate the insertion age of each copy based on the estimated intergenic mutation rate of 1.3E-8 substitutions per site per million years[35].
Full-length DNA transposons were identified by BLASTn (v2.3.1+, default parameters) searches of consensus sequences of the terminal inverted repeats (TIRs) of a given family. TIRs were required to be found in opposite orientation in a user-defined distance interval of 7,000—15,000 bp.
To produce a library of full length LTR-retrotransposons suitable for quantitative assembly completeness comparison (note 3), we required an annotation performed identically to those carried out on other assemblies (Supplementary Tables 20). We therefore implemented the methods described in Monat et al. (2019)[6] on a selection of genome assemblies shown in Supplementary Note Figure 4.1.
Tandem repeats where annotated with TandemRepeatsFinder[36] (v4.09) under default parameters (Supplementary Table 20). Overlapping annotations where removed with a priority-based approach assigning higher scoring and longer elements first. Elements which overlapped already assigned elements were either discarded (>90% overlap) or shortened (<=90% overlap) if their remaining length exceeded 49 bp.
To obtain a collection of nonredundant tandem repeat units suited for FISH probe development, the consensus sequences of the tandem repeat units (output of TandemRepeatsFinder) where clustered with vmatch dbcluster (v2.3.0) at high stringency with >=98% identity and a mutual overlap >=98% (arguments 98 98 -v -identity 98 -exdrop 3 -seedlength 20 -d -p). The 300 largest clusters with member sizes from 199 to 343 where each subjected to a multiple sequence alignment with EMBOSS (v6.6) MUSCLE[37] under default parameters. A consensus

sequence (>=70% majority) derived per cluster from the MUSCLE score file served as template sequence for the FISH probes (Online Methods; Supplementary Table 21).

3.3 Annotation of miRNAs

MicroRNA (miRNA) identification was performed by following a two-step homology-based pipeline. The sequences of 'Lo7' pseudomolecules were homologically searched for the occurrences of known plant mature miRNA sequences which were retrieved from miRBase[38] (v21; www.mirbase.org). This step was performed using SUmirFind (https://github.com/hikmetbudak/miRNA-annotation/blob/master/SUmirFind.pl), an in-house script, and the matches with no mismatch or only one base mismatch between a mature miRNA sequence and the pseudomolecule sequence were accepted[39,40]. A second in-house script, SUmirFold (https://github.com/hikmetbudak/miRNA-annotation/blob/master/SUmirFold.pl), was used to obtain precursor sequences of the candidate mature miRNAs from the pseudomolecules and assess their secondary structure-forming abilities with UNAFold[41], together with the following criteria: 1) No mismatches are allowed at Dicer cut sites; 2) No multi-branched loops are allowed in the hairpin containing the mature miRNA sequence; 3) Mature miRNA sequence cannot be located at the head portion of the hairpin; 4) No more than 4 and 6 mismatches are allowed in the miRNA and its hairpin complement (miRNA*), respectively[39,42]. The final set of identified miRNAs from the pseudomolecules was obtained by SUmirScreen script (https://github.com/hikmetbudak/miRNA-annotation/blob/master/SUmirScreen_v2.py). The resulting miRNAs were mapped back to the pseudomolecules and the genomic distribution statistics were recorded with SUmirLocate script (https://github.com/hikmetbudak/miRNA-annotation/blob/master/SUmirLocate_v2.py)[43].
Coding targets of the identified miRNAs were predicted by the web-tool psRNAtarget, using *S. cereale* coding sequences retrieved from NCBI[44,45]. Potential target sequences were compared with the viridiplantae proteins by using BLASTx[27] (v2.8.1+, arguments -evalue 1E-6 –outfmt 5). Functional annotations of the potential targets were performed using Blast2GO software[46] (v5.2). Finally, repeat contents of the pre-miRNAs were assessed with RepeatMasker (v4.0, http://www.repeatmasker.org/).

3.4 Identification and analysis of gene families

Resistance gene homologs

To investigate rye homologs of the wheat and barley genes Pm2, Pm3, Mla, Lr10 and RGA2 (GeneBank IDs given in Supplementary Table 30), homology searches were performed against the rye 'Lo7', bread wheat (cv. 'Chinese Spring')[47], and barley (cv. 'Morex')[22] genome sequences, using BLASTn[27] (v1.8.1+, default parameters). Hits with at least 80% sequence identity were visualised using dotter[48] (v4.22) for manual assessment and annotation. The obtained coding sequences were converted to protein sequences, allowing comparison with the EMBOSS (v6.6) programs WATER (emboss.sourceforge.net), ClustalW[49], or MUSCLE[37], with reference sequences and other obtained sequences to aid distinction between potentially functional full-length genes, and pseudogenes with truncations or premature stop codons.

Annotated genes were aligned using MUSCLE[37] (default parameters), and the phylogenetic relationships among them were inferred using MisterBayes[50] (GTR substitution model with gamma distributed rate, variation across sites, and a proportion of invariable sites). Manually-annotated positions of the genes Pm2, Pm3, Mla, Lr10 and RGA2 on the ‘Lo7’ pseudomolecules were compared with the annotated NLR genes identified by the gene feature annotation pipeline (described above) in order to link the genome-wide NLR analysis with the detailed analysis of the four R loci. Pairwise distances between NLRs were calculated based on the resultant tree using the cophenetic.phylo function in the R package ‘ape’[51], and multidimensional scaling on the pairwise distances was conducted with the core R function ‘cmdscale’.

PPR and mTERF genes

The ‘Lo7’ pseudomolecules were scanned for ORFs with the getorf program of the EMBOSS v6.6 package[34]. ORFs longer than 89 codons were searched for the presence of PPR motifs using hmmsearch from the HMMER[52] package (v3.2.1, http://hmmer.org) and the profile hidden Markov models (HMMs) as defined in Cheng et al. (2016)[53] for the PPR family and PF02536 from the Pfam 32.0 database (http://pfam.xfam.org) for the mTERF motif[54]. Downstream processing of the hmmsearch results for the PPR proteins followed the pipeline described in Cheng et al. (2016)[53]. A score was attributed to each PPR sequence (the sum of hmmsearch scores for all PPR motifs in the protein). In parallel, the HC and LC protein models from the gene feature annotation (described above) were screened to identify the annotated proteins containing PPR motifs. Five-hundred and twenty-six PPR models were identified in the HC and seventy-six in the LC protein datasets respectively, and scored using the same approach as with the hmmsearch results. Where putative exons identified from the six-frame translations of the genome sequence overlapped with gene models in the ‘Lo7’ annotation, only the highest scoring of the overlapping models were retained. P- and PLS-class genes with scores below 100 and 240, respectively, were removed from the annotation, as they are unlikely to represent functional PPR genes. Only genes encoding mTERF proteins longer than 100 amino acids were included in the final annotation.
Molecular markers previously mapped in relation to Rf genes were integrated in the ‘Lo7’ assembly (see Supplementary Table 28) based on BLASTn (v2.8.1) sequence similarity searches as described by Hackauf et al. (2009)[55].
NLR genes were annotated using the NLR-Annotator pipeline[56] (https://github.com/steuernb/NLR-Annotator; accessed September 2018; default settings), which associated loci with an NLR gene if at least three conserved motifs of an NB-ARC (nucleotide-binding adaptor shared by APAF-1, resistance proteins, and CED-4) domain are found. A locus is considered complete if the P-loop motif and at least one motif associated with a leucine-rich repeat is present. In total, 792 complete and 366 for partial NLR loci were identified (see Supplementary Tables 29—30).

Supplementary Note: Investigations into the repetitive genome

Extensive investigations were made of the repetitive space of the rye genome (using both element annotation and kmer-based approaches), with the goals of interrogating the completeness of the assembly (Supplementary Note Figure 4.1), the effect of repetitive sequence upon this completeness as well as on the arrangement of scaffolds into pseudomolecules (Supplementary Note Figure 4.2), the efficacy of various tandem repeats as cytogenetic tools when used as probes for FISH, the evolution of the genome size and structure (Supplementary Note Figures 4.3—4.4), and the evolutionary history of transposable elements (Supplementary Note Figure 4.5—4.6). This section presents the results of these investigations to support the arguments and comments made in the main text. The annotation of these elements is described in the previous Supplementary Note.

Supplementary Note Figure 4.1.

***Poaceae* genome assemblies compared using repetitive DNA as a proxy for completeness. a)** Mathematically defined overall repetitivity in the form of 20mer frequencies. 20mers occurring 10 times or higher covered only 16% of the draft rye genome assembly released in 2017[16], whereas in the current assembly they account for 66% which is near the target value of 71% determined from 1X genome coverage of randomly sampled Illumina reads. The gap seen around a frequency of 100,000 (arrow) relates to the highly repetitive tandem repeats, which are far better represented in the present assembly. The arrow denotes the large general increase (mostly TEs), tandem repeats are around 10e5, and are still depleted even in the new assembly. **b)** Owing to their correlation with genome size, the number of retrievable full length LTR-retrotransposons can serve as a metric for the assembly quality of difficult repetitive regions[57]. The graph shows the number of retrieved fl-LTR candidates in different genome assemblies (statistics given in Supplementary Table 20). Triangles represent earlier contig assemblies, which rarely correctly reconstructed the (almost) identical 1—2 kb long terminal repeats of fl-LTRs. The arrow shows the improvement in assembly improvement between the current rye assembly and the draft assembly published in 2017[16]. Circles denote more complete assemblies. Sb: *Sorghum bicolor*[58]; Zm: *Zea mays*[58]; Hv: *Hordeum vulgare*[22,59]; Sc: *Secale cereale*[16,60]; Td: *Triticum durum*[61].

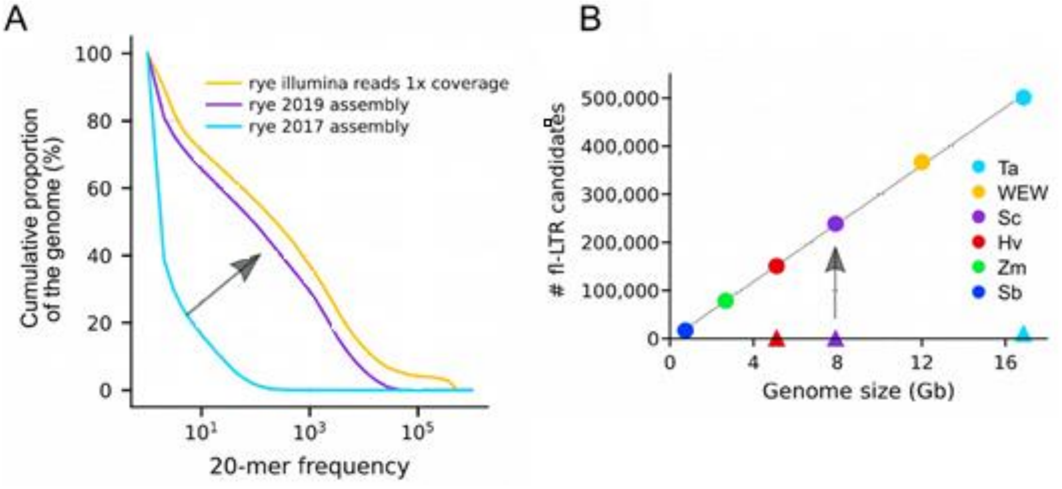

Supplementary Note Figure 4.2.

Localisation of tandem repeats in the rye assembly. a) Overall frequency of tandem repeats broken down into three main categories: (i) satellites with monomer units >= 100 bp (representing 44.6 % of all tandem repeats), (ii) minisatellites with 10—99 bp units (52%) and (iii) microsatellites with 2—9 bp units (3.4%). **b)** Chromosomal distribution of the three different tandem repeat types. Satellites are prominently located at several chromosome ends: short arms of chr 1R to 6R, most on 5RS and long arms of 2R and 3R. Most of the satellite sequences could not be assigned to a position on one of the seven chromosomes, and these where merged into chrUn. Minisatellites have two notable hot-spots on chr5L and are found in higher concentrations in the centromere. These can be used to identify the centromere in the assembly. Microsatellites are distributed more or less across all chromosomal regions.

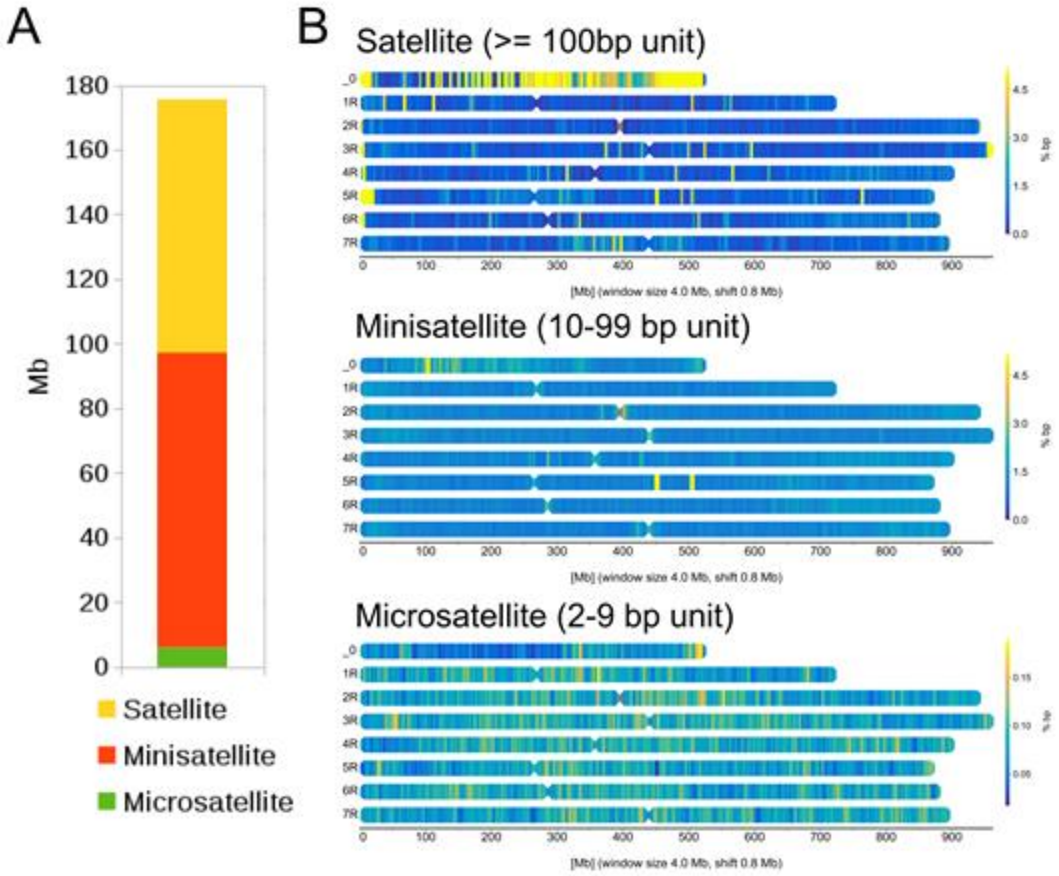

Supplementary Note Figure 4.3.

***In silico* prediction and fluorescent in situ hybridisation (FISH) profiles for eight *de novo* identified tandem repeat families** (Supplementary Table 19). Each subfigure shows the chromosomal distribution of tandem repeat sequence clusters in the assembly (left, bar charts) and their locations on the seven rye chromosomes as revealed by FISH (right, photograph and ideogram). The probes designed to Sat20/517 bound non-specifically under the tested hybridisation conditions (methods). The two satellite tandem repeat families from which the probes ScSat213 and ScSat380 appear to bind telomeric repeats which, notably, are displaced or not detectable from the subtelomeres wherever chromosomal rearrangements in the rye lineage have affected the structure of those regions (chromosomes 4R—7R). However, the predicted position of ScSat213 is in interstitial is in scattered interstitial clusters, while the only two significant clusters of ScSat380 are predicted at subtelomeres. Both these observations as evidence that subtelomeric clusters tend to be collapsed or unassembled, since ScSat380, while present on all fourteen subtelomeres only appears in the assembly at two, and ScSat213 at none. A possible reason that comparatively minor assembled interstitial clusters are overemphasised in the bar chart. The remaining five of eight FISH probes bound as predicted by the annotation (see also Supplementary Table 21). Among them is ScSat44 which represents a new 5R specific probe. The bar in the upper right image represents approximately 10 µm and the boxed chromosomes have been digitally moved to fit into the frame.

(continued over …)

(Supplementary Note Figure 4.3. continued)

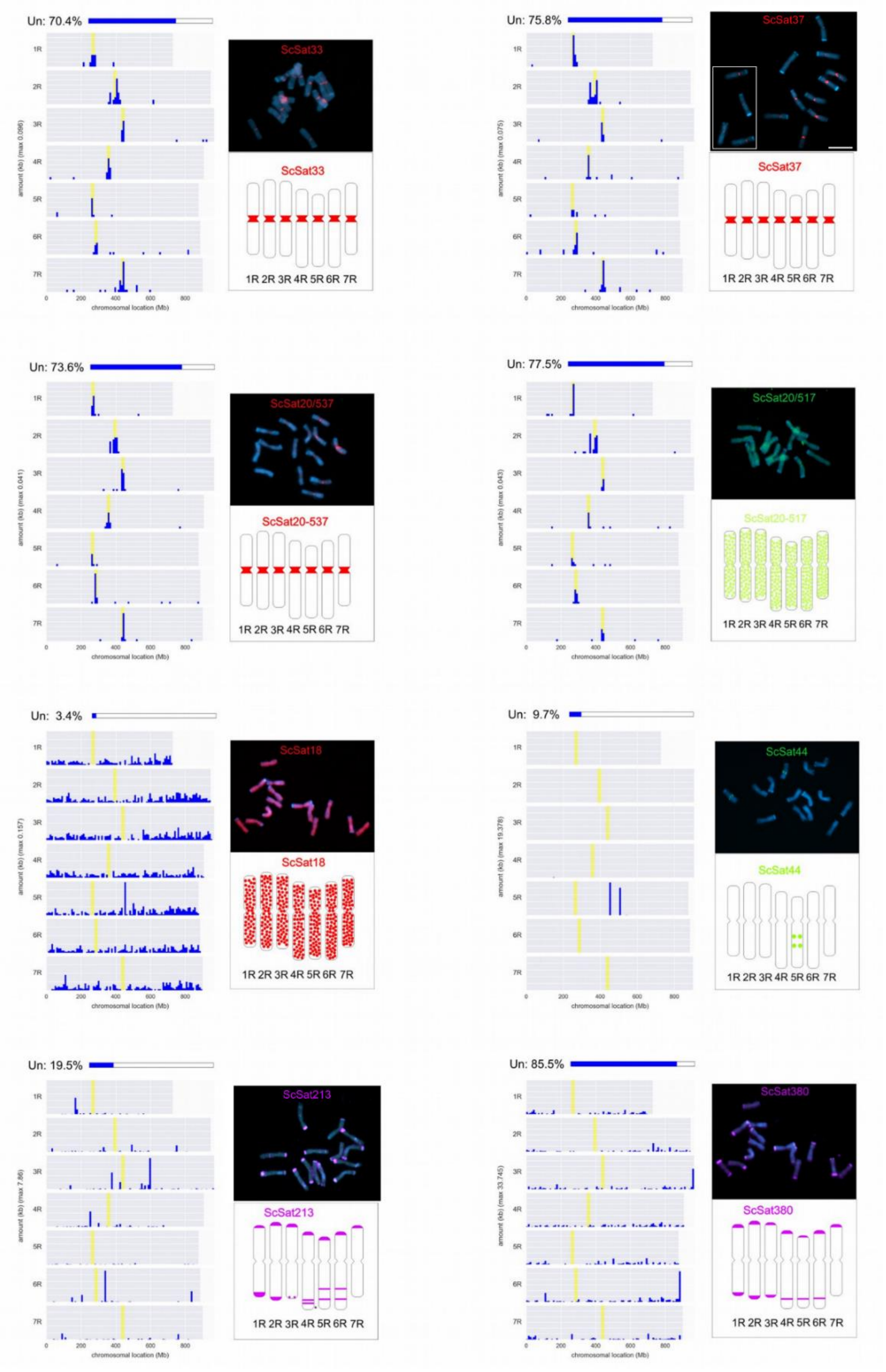

Supplementary Note Figure 4.4.

Kmer repetitiveness compared between rye and barley, chromosomes 1—7 shown left-to-right. The overall increase factor in assembly size from barley[22] to rye is about 1.5 (4,507 vs 6,735 Mbp), which is suspected to be accounted for largely in repetitive sequence, and the bars below the chromosome numbers depict the size increase factor for the short and long chromosome arms respectively, which show (except for chromosome 6) no large deviations from the overall 1.5 increase. The two line charts track changes in repetitivity along the chromosomes (median 20mer frequencies over 4 Mbp sliding windows with a 0.8 Mb shift) for barley (A) and rye (B). Rye displays a similar division into chromosomal compartments of different repetitivity grades as previously described for barley[22]. The suspected introgressions that affected the transposon profiles at the terminal long arms chromosomes of 4R and 6R (Main Text Figure 1d—g) may also have caused local reductions in the 20mer repetitive content.

Tracks C and D compare rye-barley syntenic blocks (colour-coded to correspond to the colours assigned to barley chromosomes in Main Text Figure 1a) with ideograms for FISH probes ScSat213 and ScSat380, to emphasise the correspondence between terminal rearrangements and the displacement of telomeric FISH hybridisation signals (see fig 4.3).

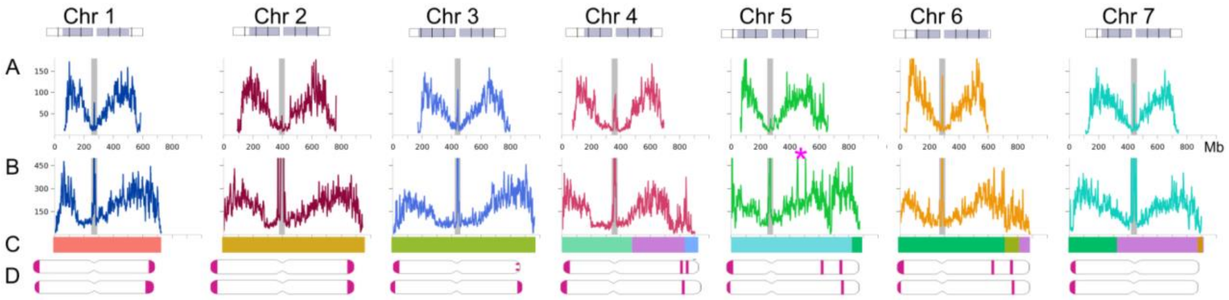

Supplementary Note Figure 4.5.

Distributions of annotated TE families along the ‘Lo7’ pseudomolecules. a) Distribution of main TE families along rye chromosomes. Shown are the number of full length copies in bins of 40 Mbp. Note that the short (left on the image) ends of 4R and 6R have a different TE composition than the rest of the genome. **b)** Contribution (in %) of diagnostic TE families to the terminal 150 Mbp of chromosomes 1R, 2R and 4R. Some TE families are strongly enriched on 4R while others are depleted or virtually absent. **c)** Examples of distributions of TE families along chromosome 4R. Colours correspond to different TE subfamilies.

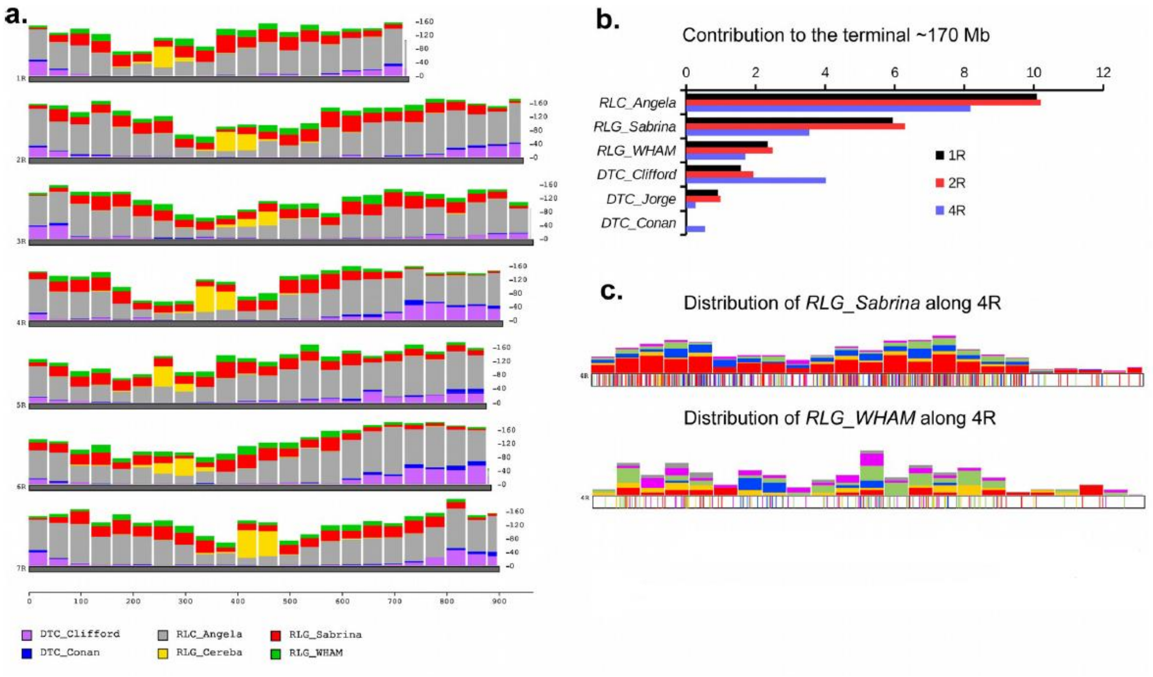

Supplementary Note Figure 4.6.

Relative abundance of four major TE families in the terminal 175 Mbp of all rye chromosomes. Here, we counted all full-length copies of the four TE families in the terminal 170 Mbp of the long chromosome arms. Note that chromosome 4R has a composition distinct from all others with much higher numbers of DTC_Clifford elements while RLG_Sabrina and RLG_WHAM are practically absent, which we interpret as evidence of possible ancient translocation events.

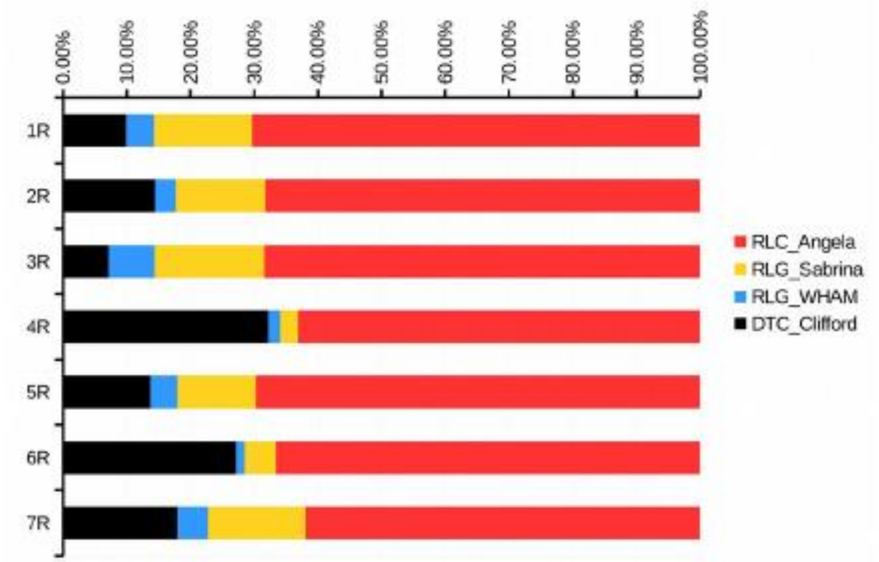

Supplementary Note Figure 4.7.

A model for the evolution of chromosome 4R. Chromosome 4R is a composite chromosome that consists of segments of ancestral Triticeae chromosomes (as inferred by rye-wheat-barley collinearity[60]) 4, 6 and 7, that underwent rearrangement in the rye lineage since its split from wheat and barley. The segments are indicated and the orientation of the ancestral chromosome segments is indicated with arrows. The rye ancestor diverged into different lineages. In the 'Lo7' lineage, RLG_Sabrina and RLG_WHAM retrotransposons were very active, while in a second, hypothetical, lineage, the CACTA family DTC_Clifford was highly active. At least 1.8 million years ago, the two lineages recombined, leading to the introgression of a terminal ~170 Mbp segment into the 'Lo7' lineage. Subsequently, the genome was invaded by RLC_Angela retrotransposons.

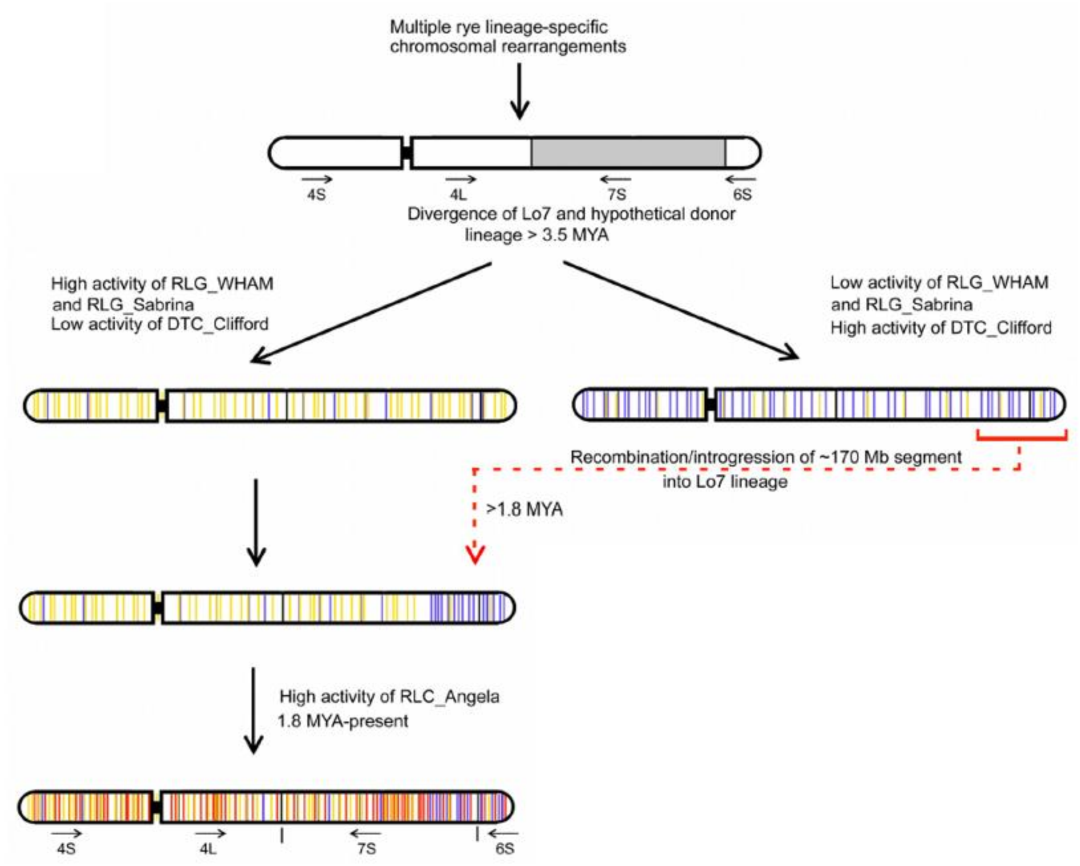

Supplementary Note: Detailed gene-level collinearity analysis of rye, barley, and wheat

We used a best-reciprocal-matches approach with transcriptome data to create high resolution collinearity maps between rye and its Triticeae relatives barley[22] (*Hordeum vulgare* cv. Morex), and the three subgenomes of bread wheat[47] (*Triticum aestivum* cv. Chinese Spring; Methods). Supplementary Note Figures 5.1—5.7 break down the results to show collinearity between every chromosome of every genome. As noted in the main text, the curvature of collinear regions indicates changes in the rate of genome expansion/contraction since genetic isolation between two species. The colour key, which applies across the whole note, relates to the homologous group of the chromosome on the horizontal faceted axis.

Supplementary Note Figure 5.1.

Chromosome-wise best-reciprocal-matches showing Triticeae collinearity for chromosome homologous group 1.

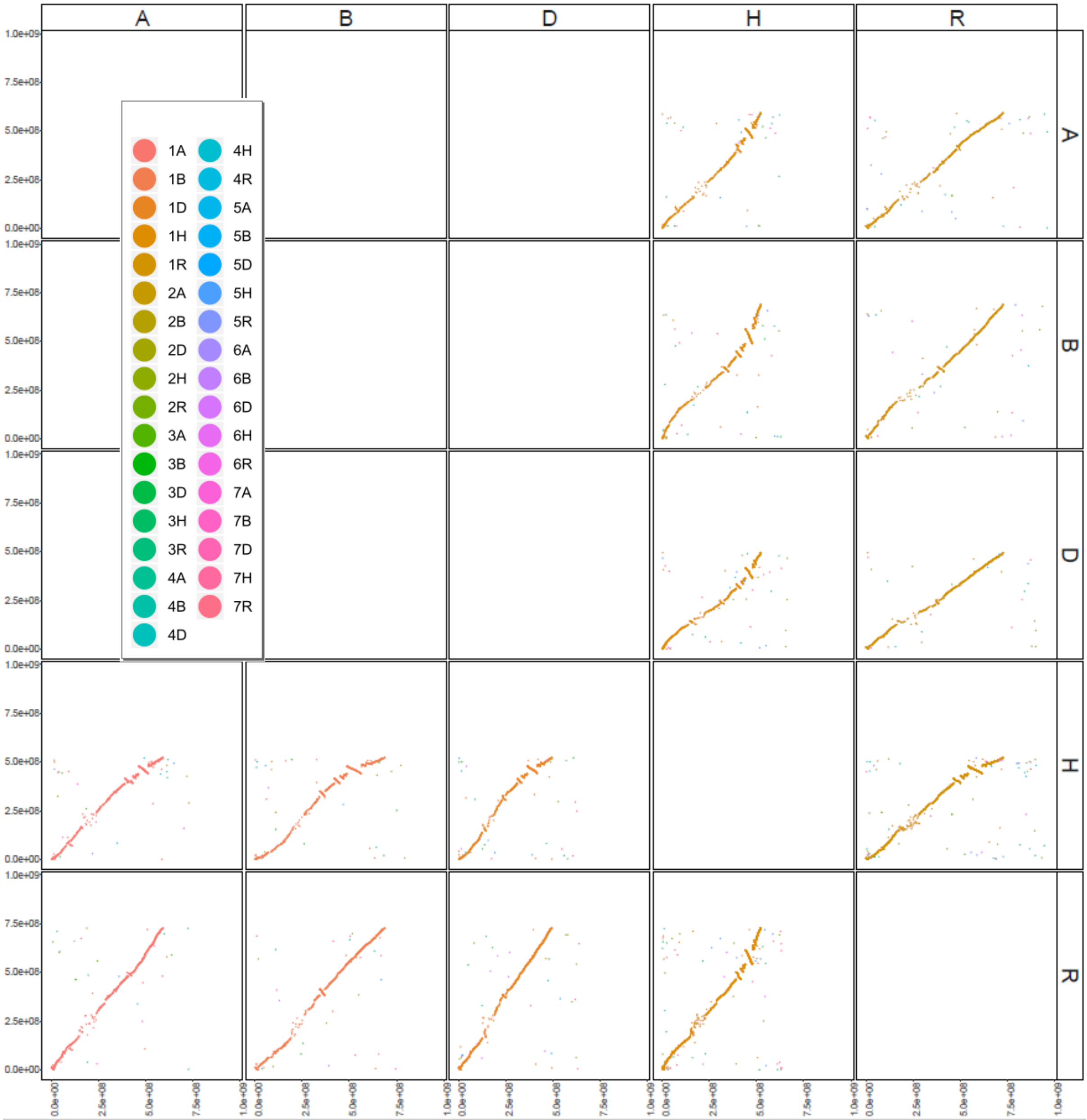

Supplementary Note Figure 5.2

Chromosome-wise best-reciprocal-matches showing Triticeae collinearity for chromosome homologous group 2.

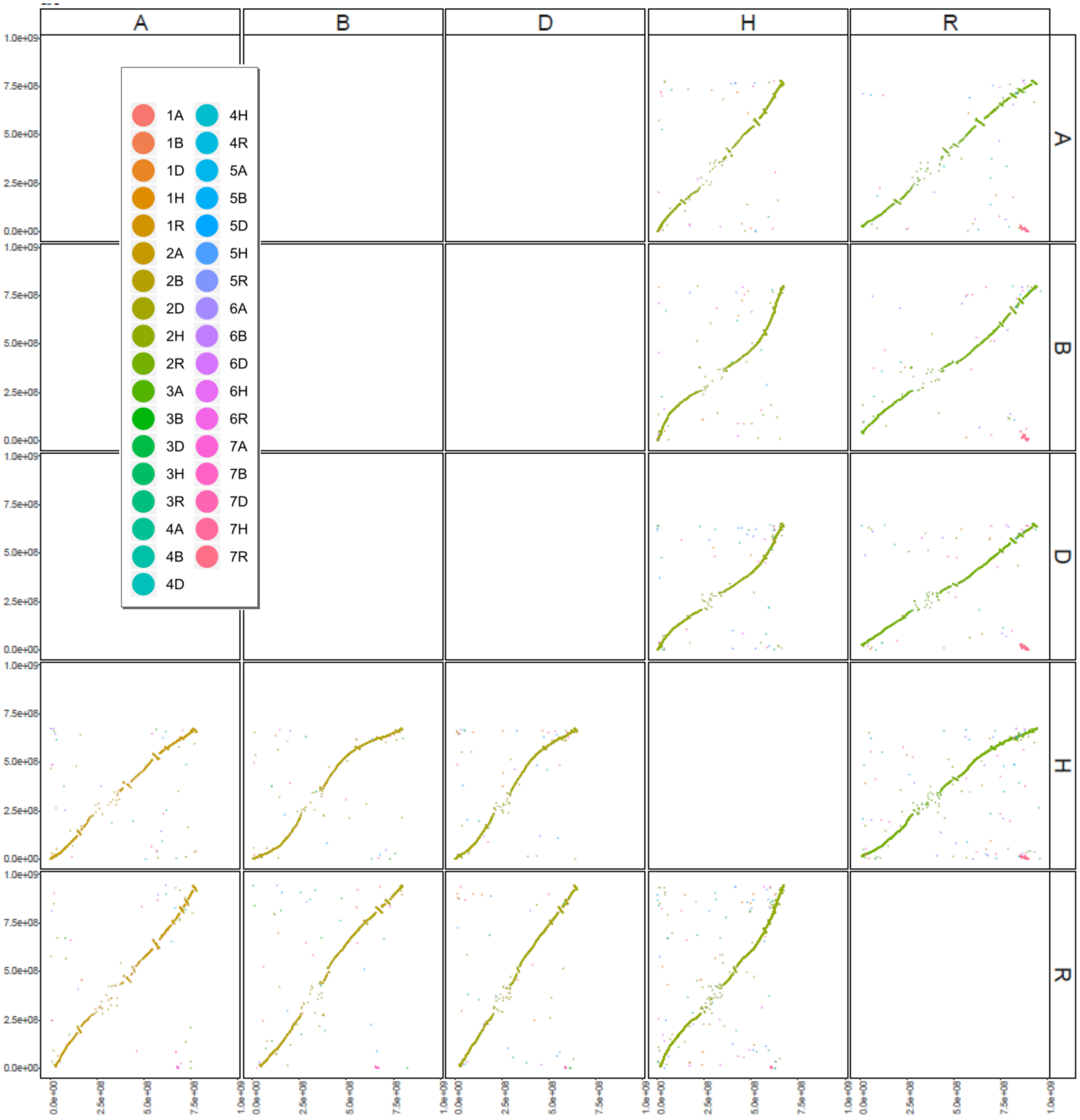

Supplementary Note Figure 5.3.

Chromosome-wise best-reciprocal-matches showing Triticeae collinearity for chromosome homologous group 3.

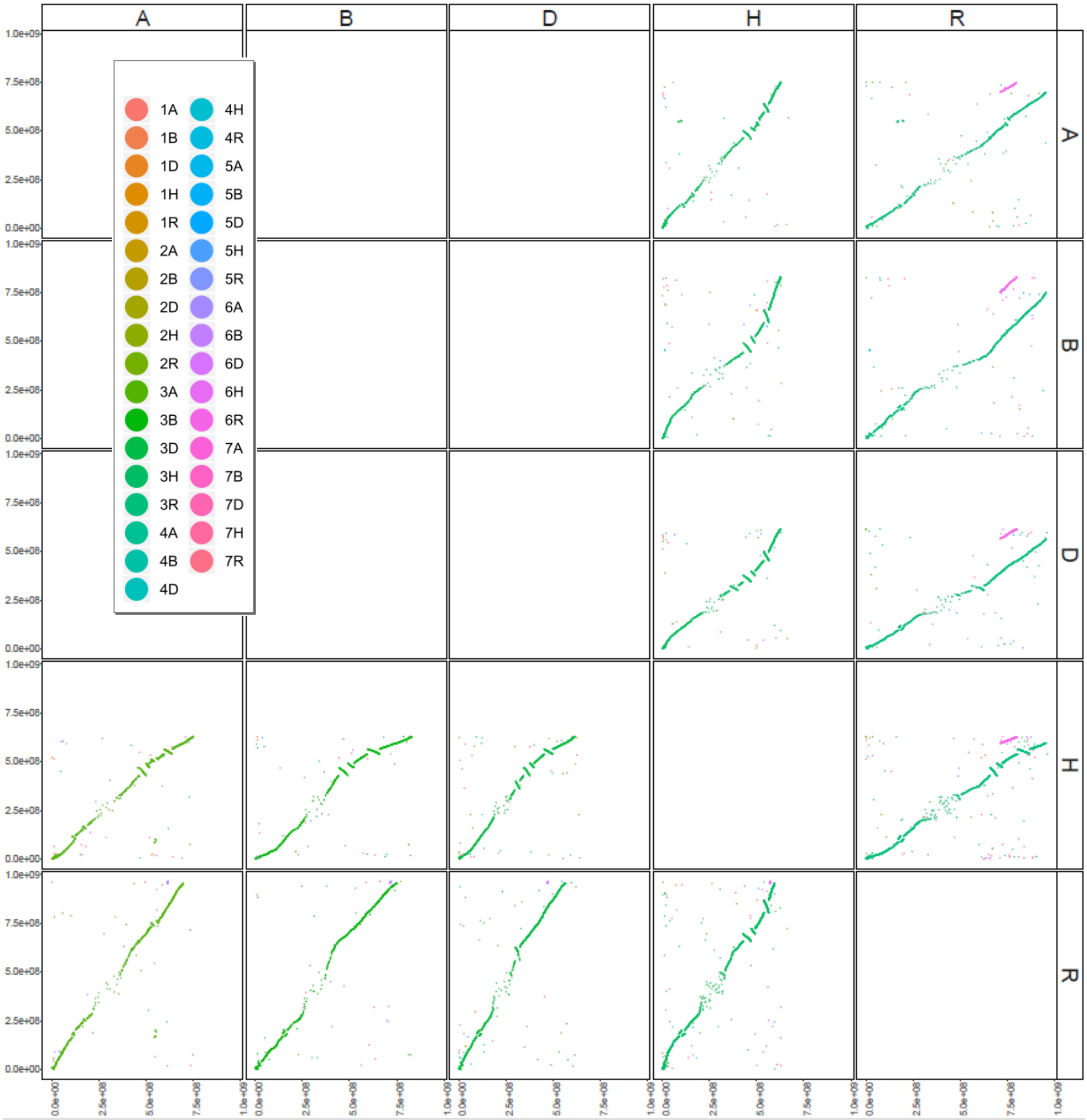

Supplementary Note Figure 5.4.

Chromosome-wise best-reciprocal-matches showing Triticeae collinearity for chromosome homologous group 4.

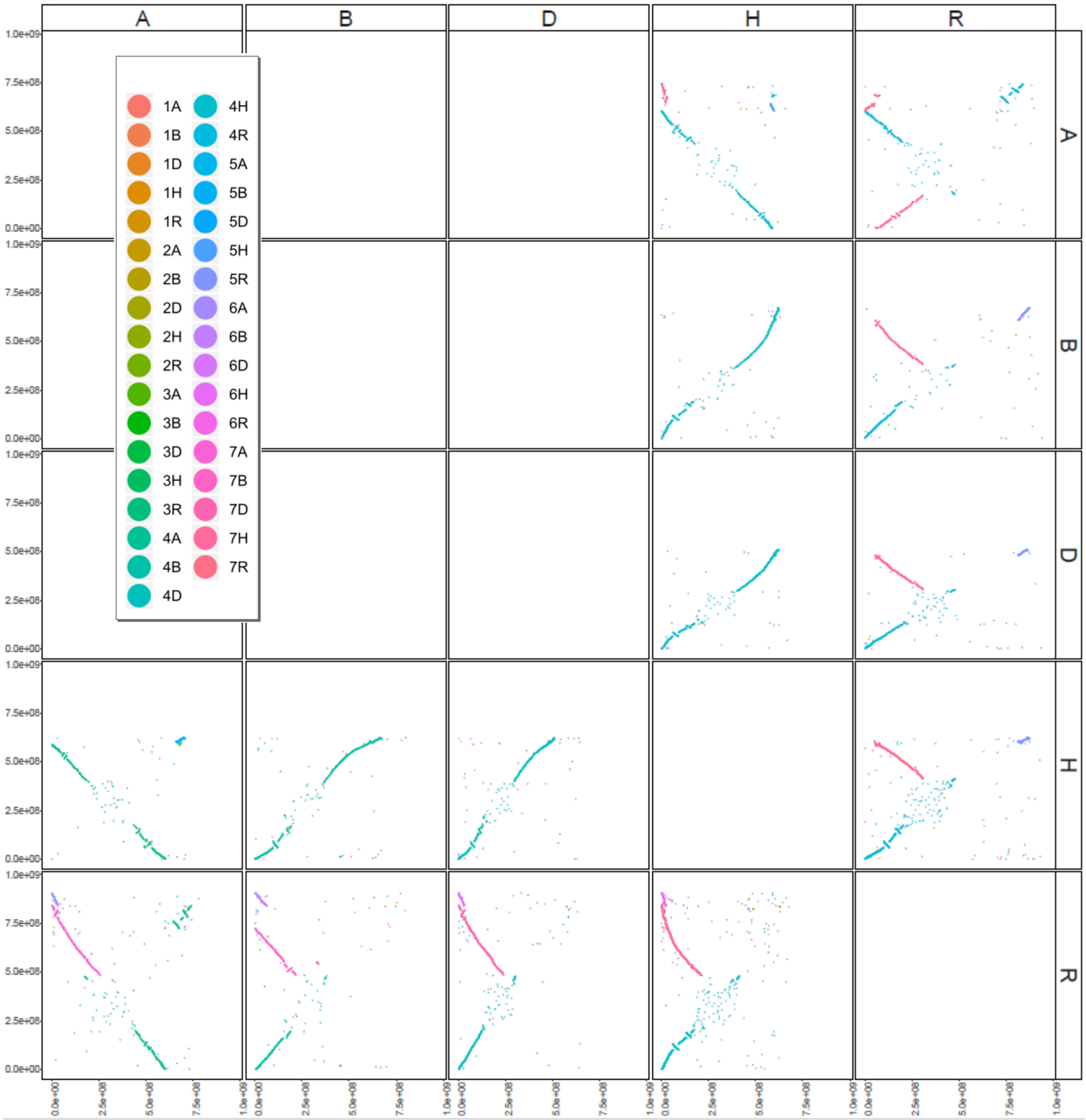

Supplementary Note Figure 5.5.

Chromosome-wise best-reciprocal-matches showing Triticeae collinearity for chromosome homologous group 5.

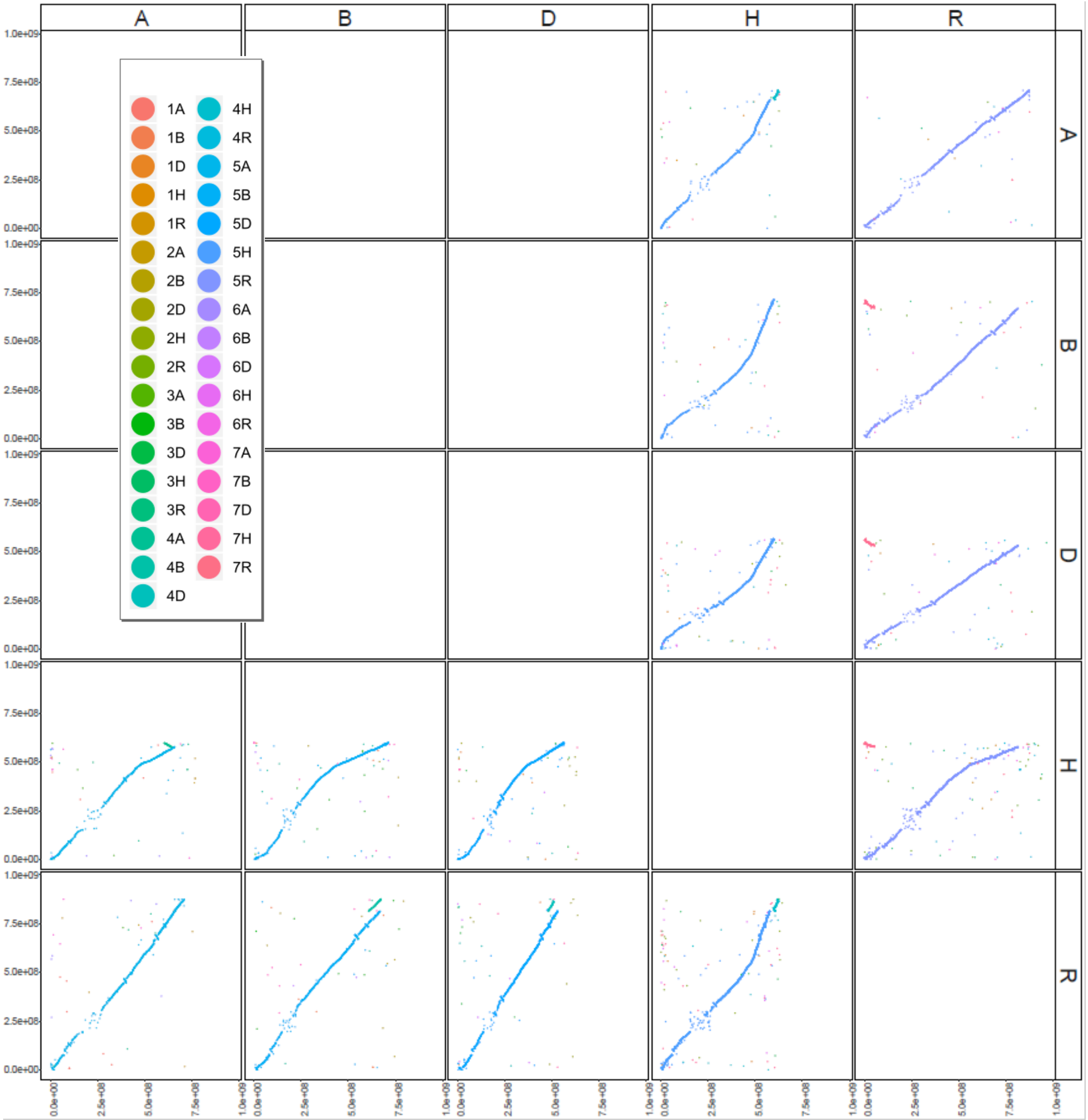

Supplementary Note Figure 5.6.

Chromosome-wise best-reciprocal-matches showing Triticeae collinearity for chromosome homologous group 6.

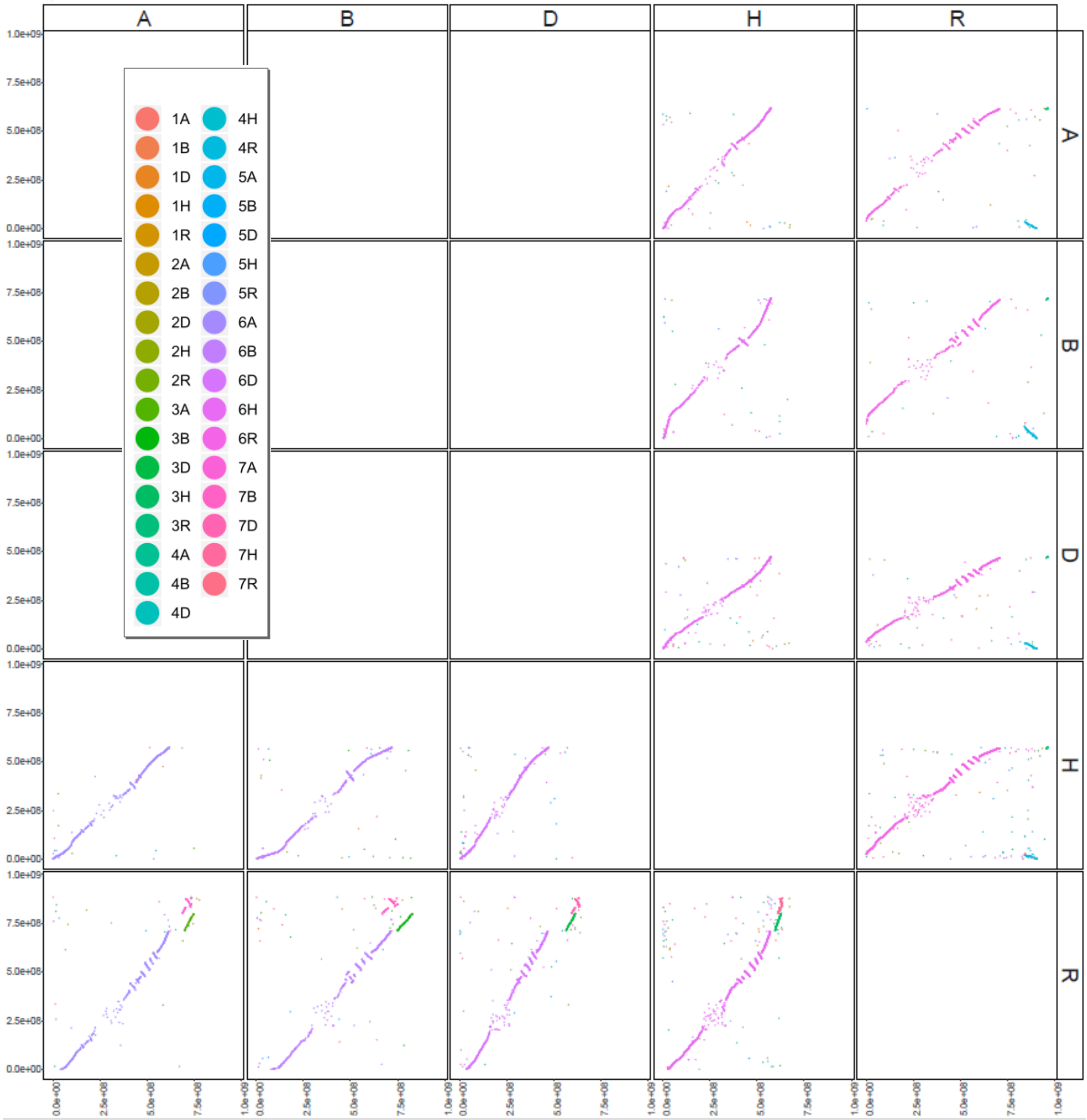

Supplementary Note Figure 5.7.

Chromosome-wise best-reciprocal-matches showing Triticeae collinearity for chromosome homologous group 7.

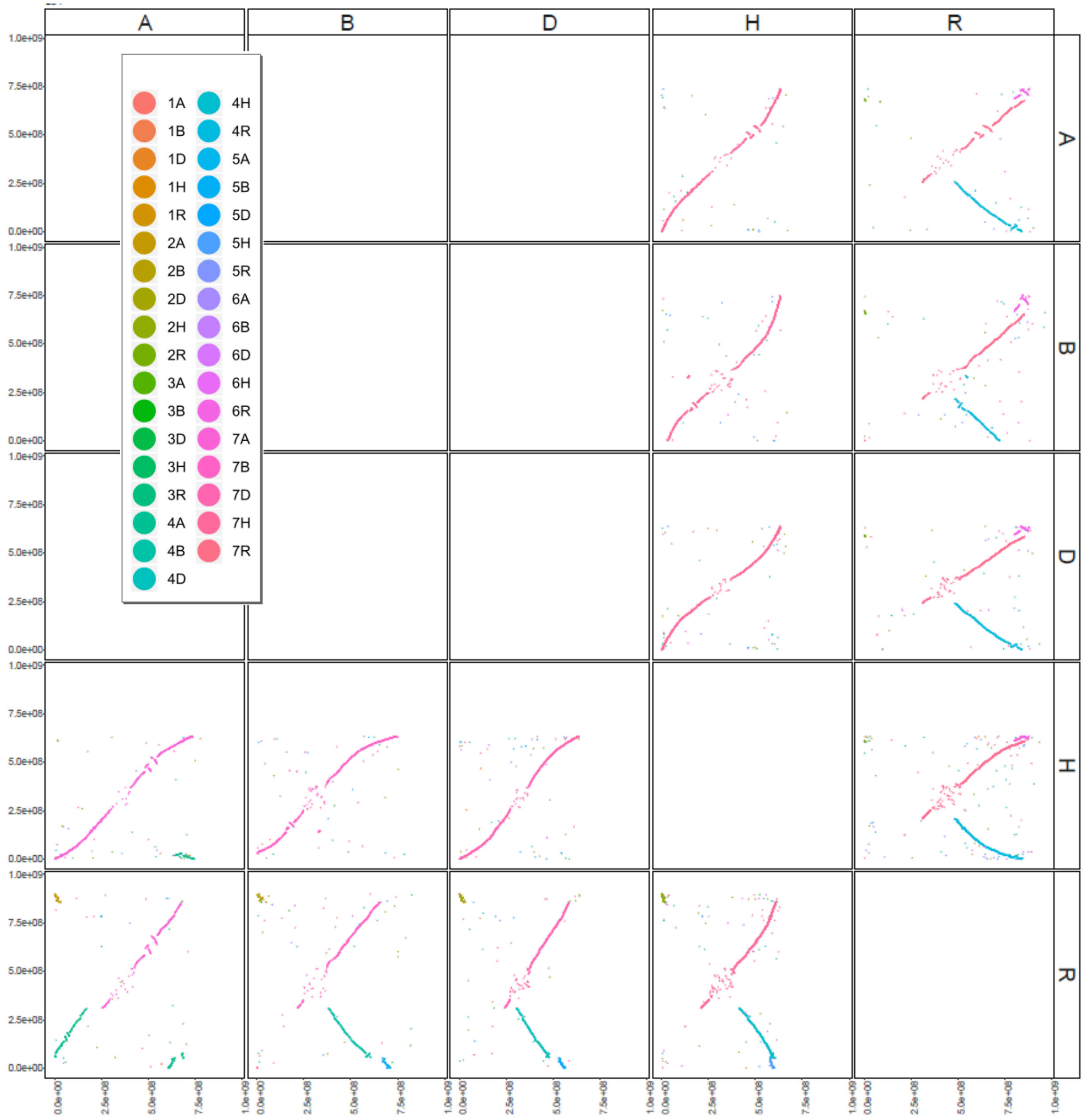

Supplementary Note: *Secale* diversity and segregating structural variations

We expand here upon the discovery of structural variants (SVs) using Hi-C as presented in the main text, the statistical test that was run to affirm association between SVs and the peri-centromeric low-collinearity modules.

Supplementary Note Figure 6.1.

Hi-C asymmetry plots revealing Structural Variations (SVs) between the genomes of ‘Lo7’ and representatives of the genus *Secale*. The four relatives studied are related to the reference line ‘Lo7’ as follows: [[*S. cereale* line ‘Lo7’, *S. cereale* line ‘Lo225’], *S. vavilovii*], [*S. strictum*, *S. sylvestre*]. SVs result in result in discontinuities in the ratio of Hi-C links mapping left:right (r) relative to ‘Lo7’ (r_{sc}). Inversions often produce clean, diagonal lines. Indels, translocations, and deletions all cause interruptions, but identification of all SV types can be confounded by sequence errors, contiguous/overlapping SVs, low mapping rates when aligning reads from divergent genomes, and other factors. Visually-identified candidate SVs are shaded, with common non-grey colours indicating possible synapomorphic (shared, derived) SVs. Shading is omitted from some anomalies at centromeres where missing sequence causes artefacts. Blue lines denote the boundaries of the low collinearity regions for each chromosome. The rightmost inversion marked on 5R corresponds to the region of recombination suppression marked on Main Text Figure 1a. The observed SVs are consistent with the inferred phylogenetic relationships and limited horizontal genetic exchange since all putatively shared SVs appear to be synapomorphic. This is expected because large SVs not only reflect, but also likely reinforce, reproductive barriers between groups.

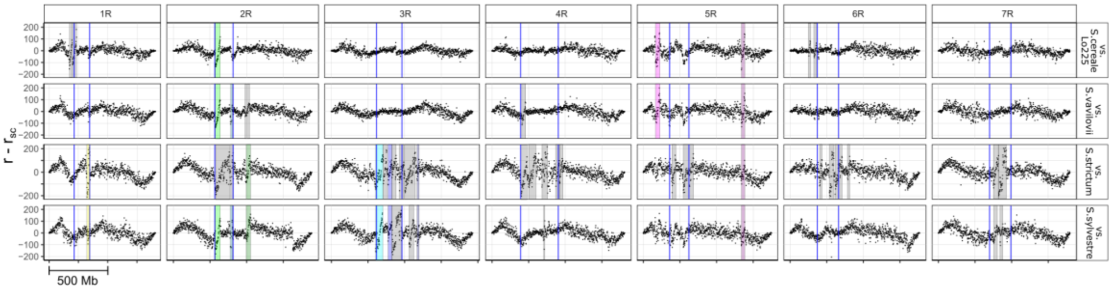

Supplementary Note Figure 6.2.

Testing for association of candidate SVs and low-collinearity pericentromeric regions in rye. A permutation method was used to confirm that large SVs show an affiliation for low-collinearity pericentromeric regions (defined as delineated in Main Text Figure 1a—g). Candidate SVs (as marked on Supplementary Note Figure 6.1) were randomly reshuffled over the chromosomes. Overlaps were not allowed, and synapomorphic SVs were placed at the same positions on multiple genomes. The count axis records the number of times SVs overlapped the low-collinearity region under random placement (10,000 iterations). The black vertical line shows the number of LCMs overlapping in the dataset (1). Testing the hypothesis that the data are produced by randomly-placed SVs results in a p-value of < 1/10,000, demonstrating that SVs almost certainly tend to occur in association with these areas of disrupted collinearity.

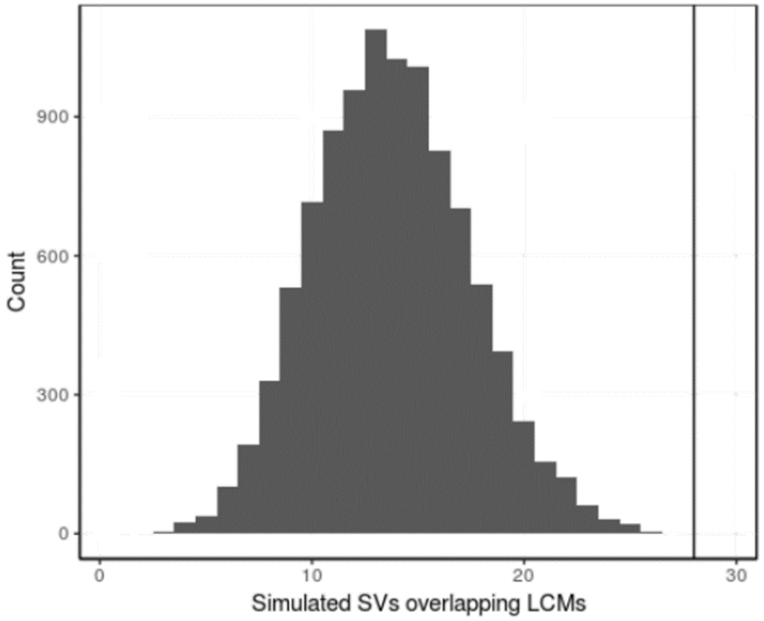

Supplementary Note: Investigations on the evolution of the Triticeae focussing on rye

Supplementary Note Figure 7.1.

Diversity and relationships among Secale taxa represented as a dendrogram, and grouped by taxonomic assignment. The population structure corresponds to the structure of three taxa as presented in Schreiber et al. 2019 but gives a clearer grouping due to the additional wild accessions, especially with regard to *S. vavilovii*, the wild progenitor, which was previously indistinguishable from domesticated rye now forms a subgroup within *S. cereale*. a) Neighbour-joining tree, with taxonomic assignments to subspecies level, according to genebank passport data. b—d) The first three prinicipal components of genetic variance within the dataset, with samples coloured according to species.

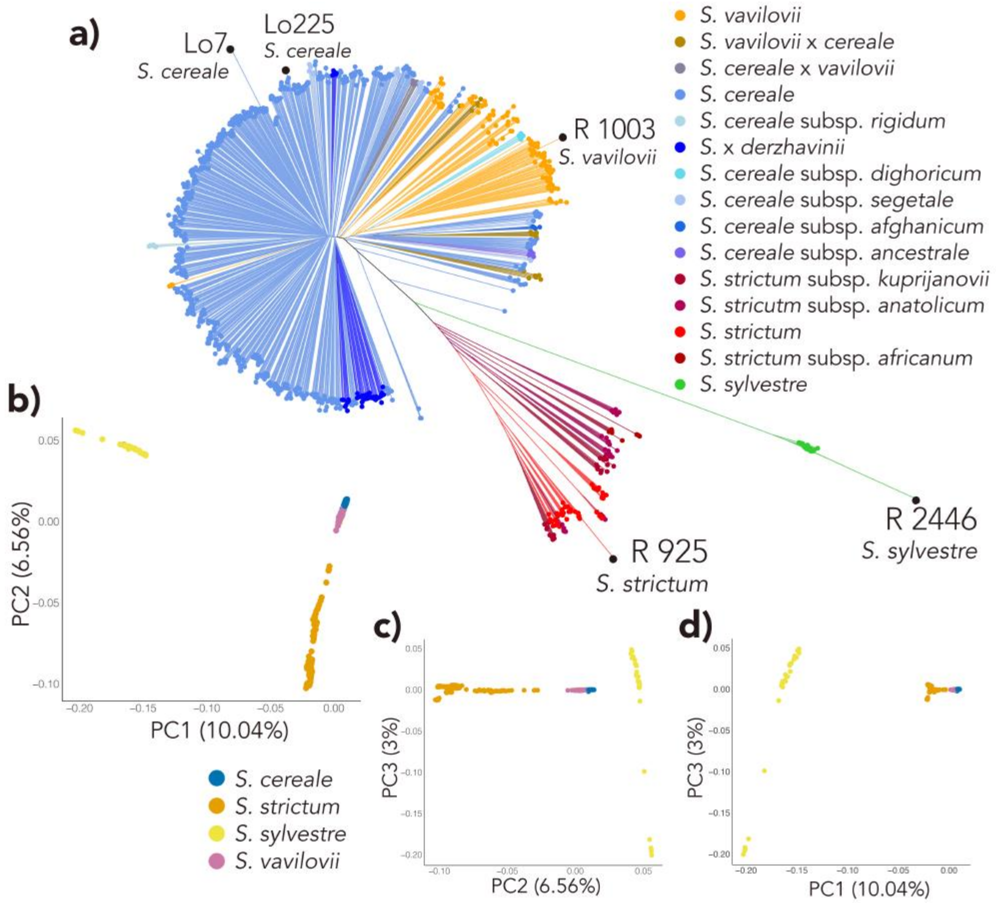

Supplementary Note Figure 7.2.

Evolutionary distances between the A, B, C, and H (sub)genomes and rye (‘Lo7’), inferred using the percent identity of reciprocally best BLAST matches (highest e-value, then highest bitscore, then longest match) between CDS sequences of wheat (cv. ‘Chinese Spring’) and barley (cv. ‘Morex’). Percent identities were averaged over bins of 150 matches, in increments of 20. The position of a bin was taken as the mean position of matches within the bin.

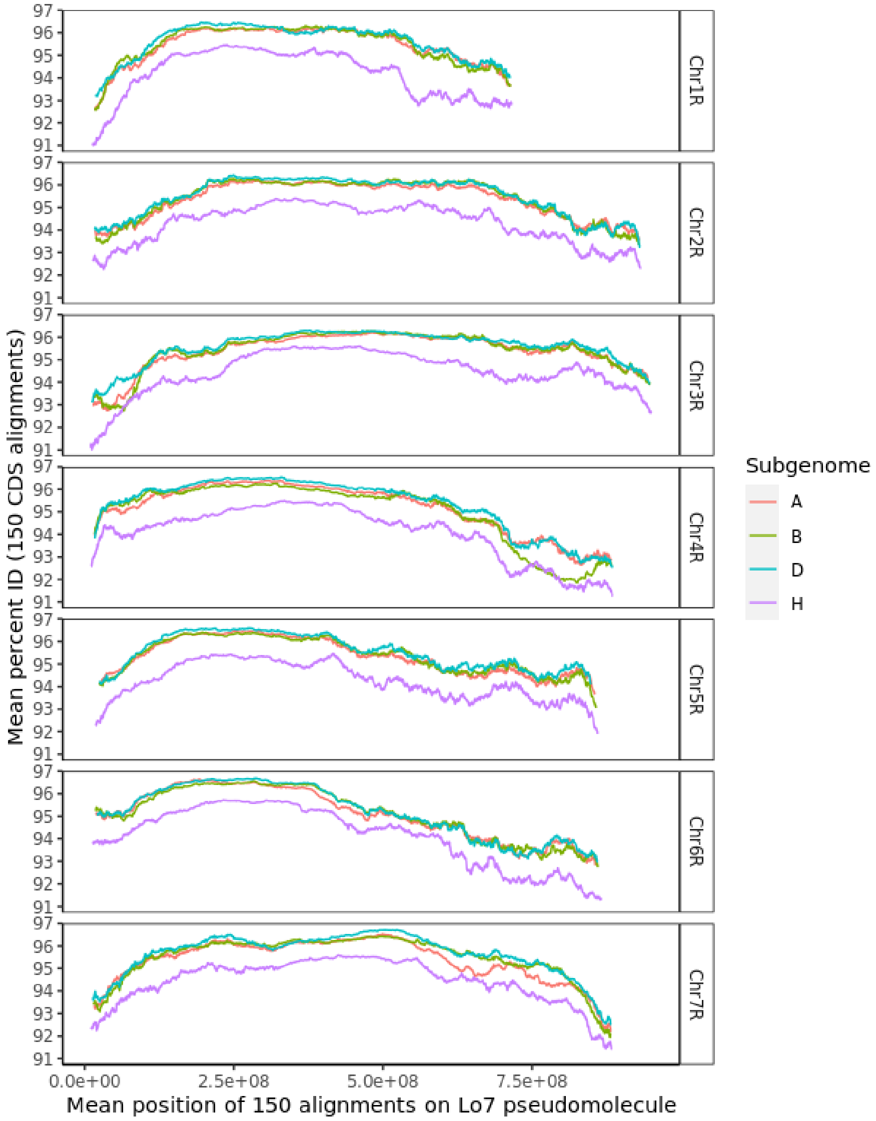

Supplementary Note Figure 7.3.

Pn/Ps and Fst statistics calculated in bins across the chromosomes 1R—7R. Figure features are identical those in Main Text Figure 2e/f (lower two subpanels), but not all chromosomes were included there owing to space limitations.

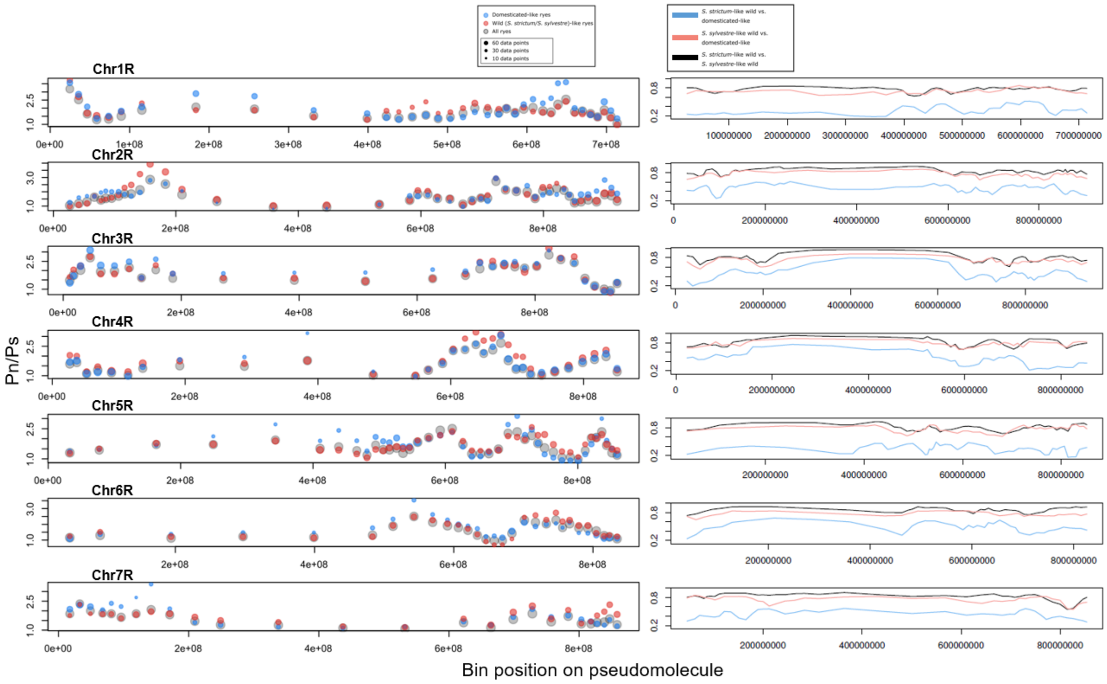

Supplementary Note Figure 7.4.

Figure features including colour-cluster assignments are identical those in main text figure 2e/f (upper subpanels), but not all chromosomes were included there owing to space limitations.

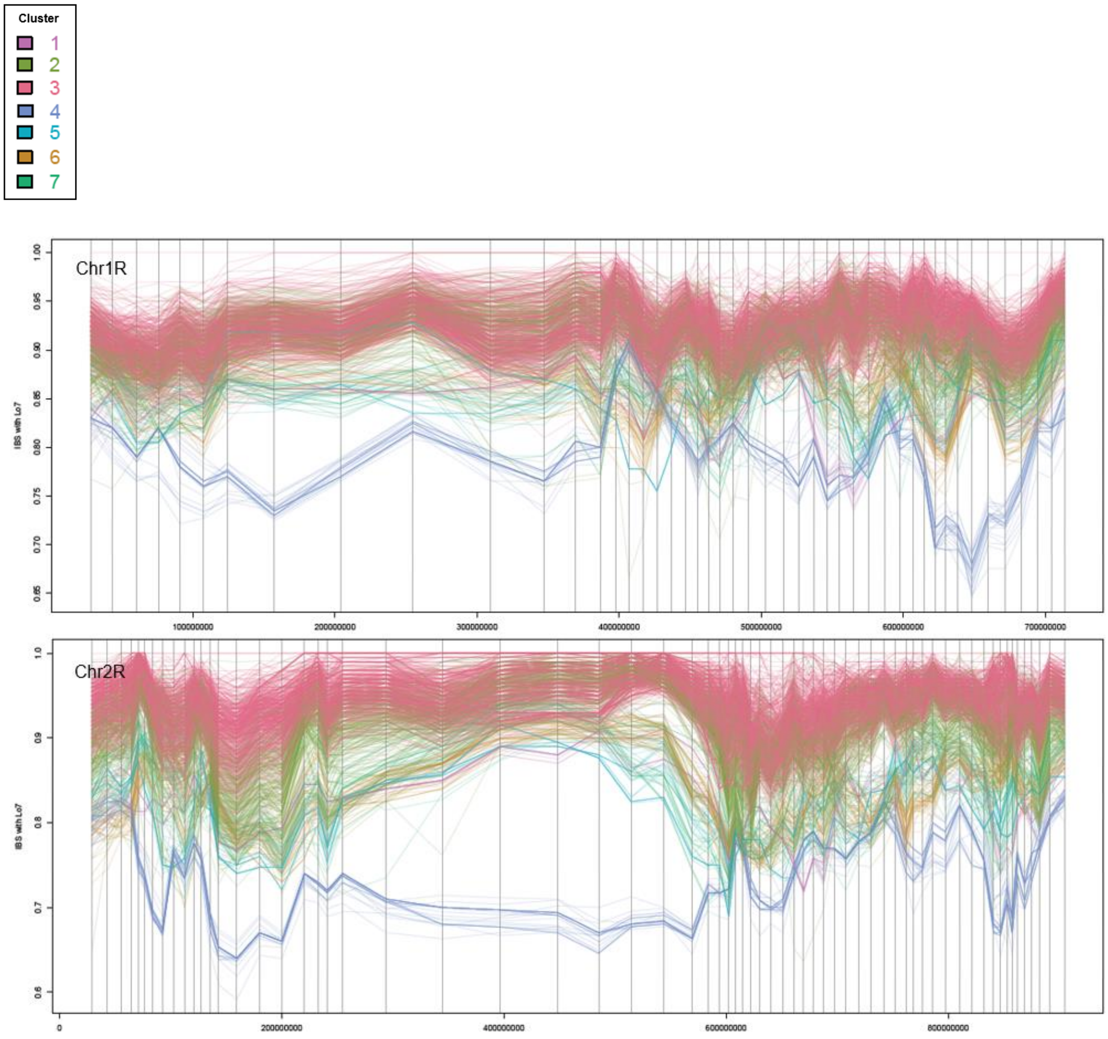

(continued over …)

(Supplementary Note Figure 7.4 continued)

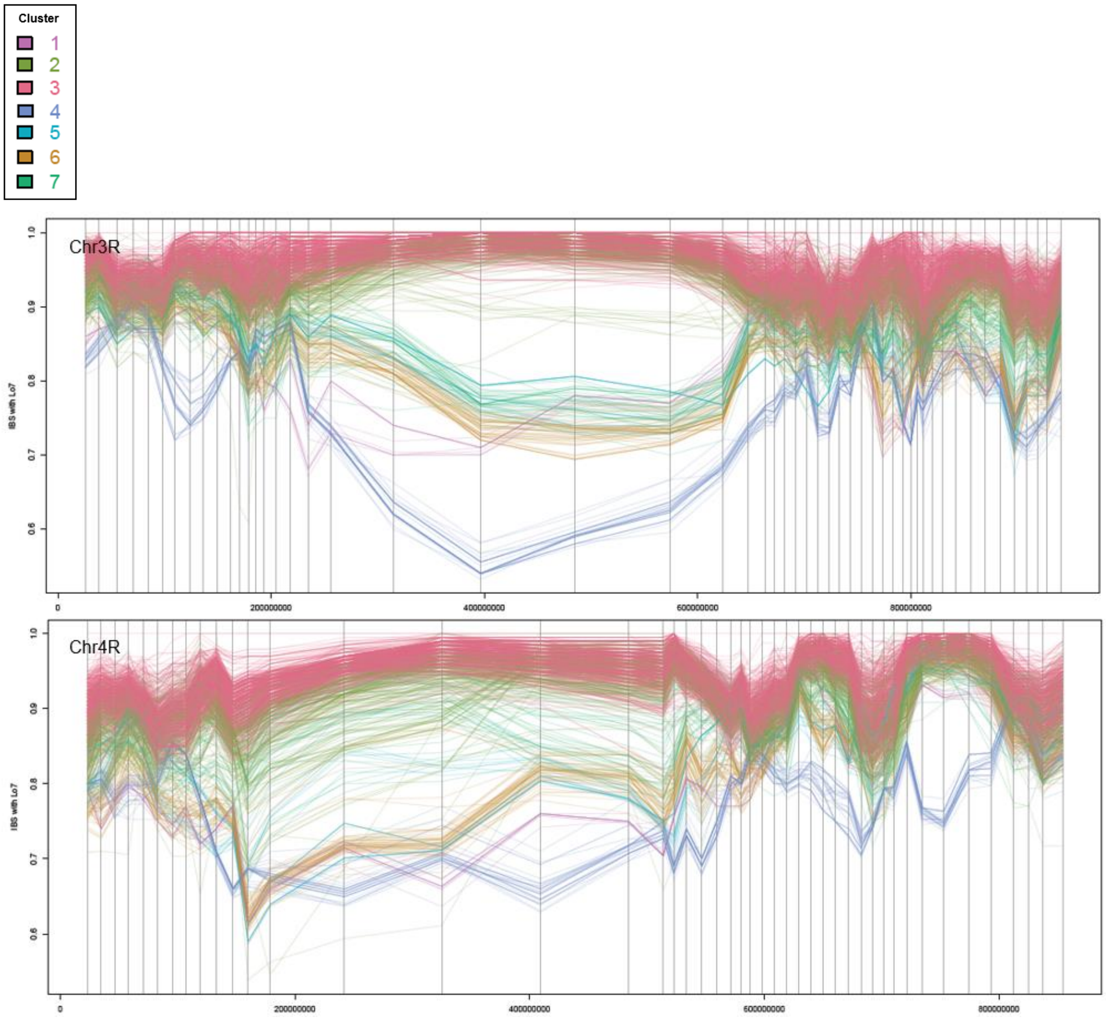

(continued over …)

(Supplementary Note Figure 7.4 continued)

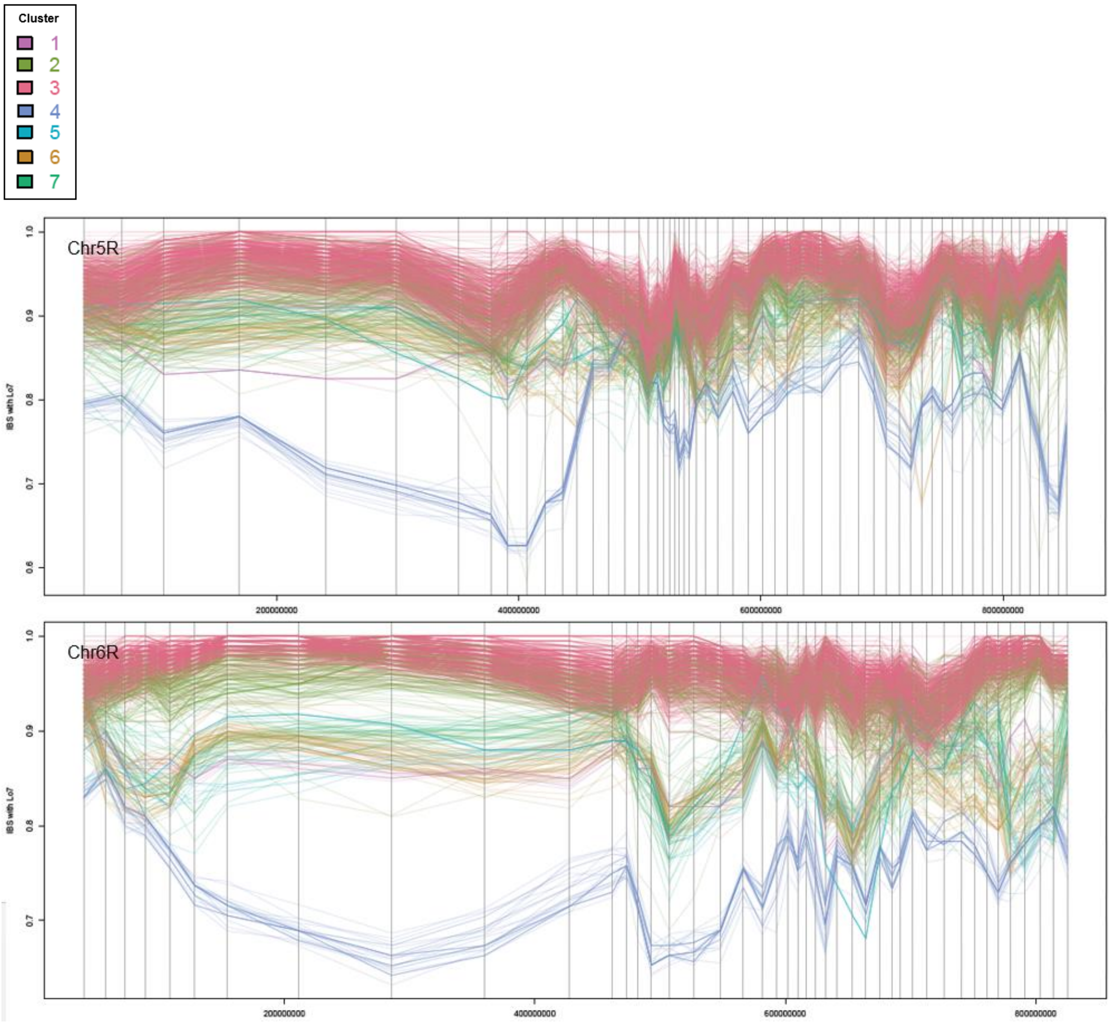

(continued over …)

(Supplementary Note Figure 7.4 continued)

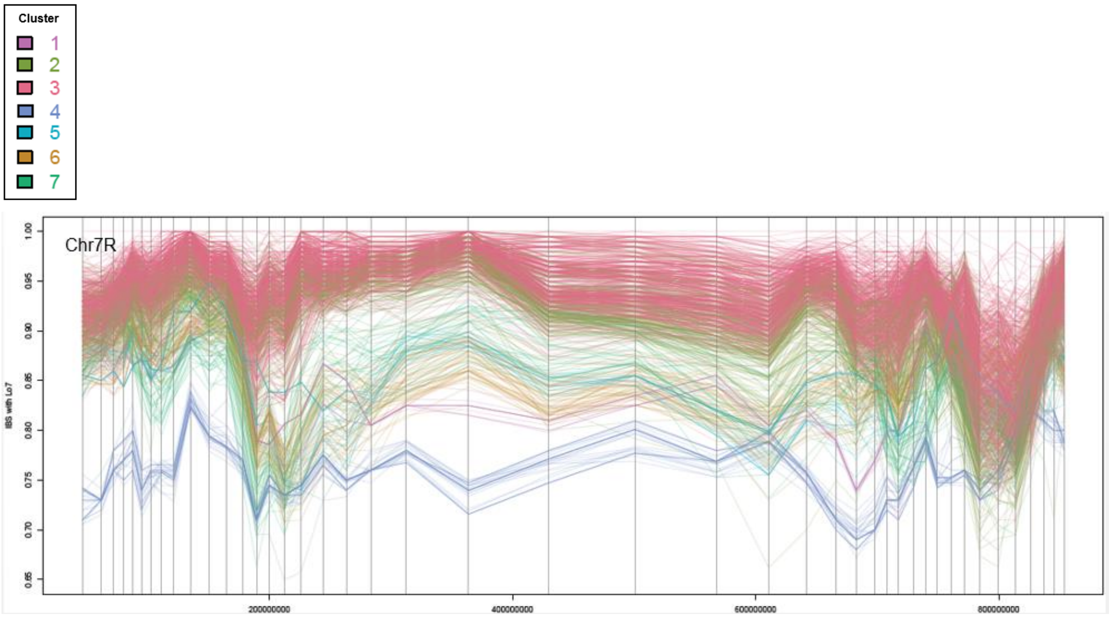

Supplementary Note: Rye-wheat introgression lines classified using high-throughput shotgun sequencing

High throughput sequence data can be effectively utilised to characterise rye introgressions into wheat based on comparative mapping depths (Online Methods). This is easiest to appreciate by appraising a large number of different introgression haplotypes visually, which we provide here in Supplementary Note Figure 8.1, to supplement the smaller number shown in Main Text Figure 3b. By calling variants in the data, we were able to test the supposed common origins of the respective 1AL.1RS and 1BL.1RS introgressions (Online Methods; Supplementary Figure 8.2).

Supplementary Note Figure 8.1.

A sample of genotypes from each of four bread wheat diversity panels displayed in groups according to the automated classification of rye chromatin introgressions. Row labels have the format [Classification]_[Panel]_[Sample ID]. Samples classified as “ambiguous” tend to have a moderate overabundance of 1RS reads, and underabundance of 1AL or 1BL, though far less pronounced than in the more confidently assigned samples. The causes of this ambiguity are currently unresolved. Sequencing depth does not appear to be a factor. Sample cross-contamination or barcode misassignment are possible but unlikely at this scale.

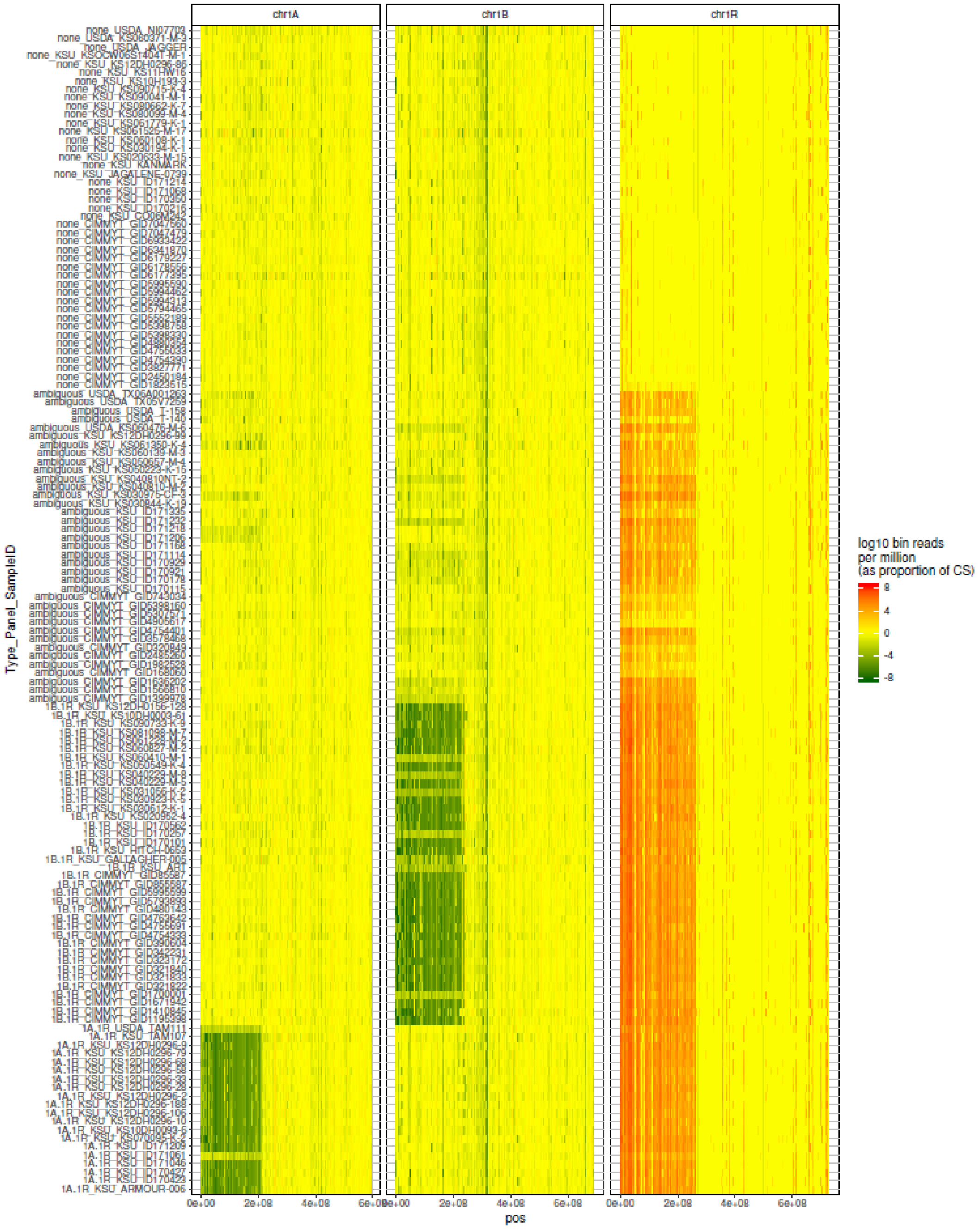

Supplementary Note Figure 8.2.

Sequence identity-based dissection of the commonality of 1RS translocation donors. Predicted 1RS carriers were selected to form a combined 1RS panel, in which SNPs were identified and pair-wise identity by state (IBS) percentages calculated. The square root values of percent different calls were used to derive a heatmap for all pair-wise comparisons. The heatmap dendrogram unambiguously shows two major clusters, consistent with common origins of each 1RS donor. The larger cluster corresponds to 1BL.1RS class, and the smaller cluster corresponds to 1AL.1RS class. Within 1BL.1RS class, sequencing technology appears to play a role, as exome sequenced lines show a subcluster slightly different from GBS lines.

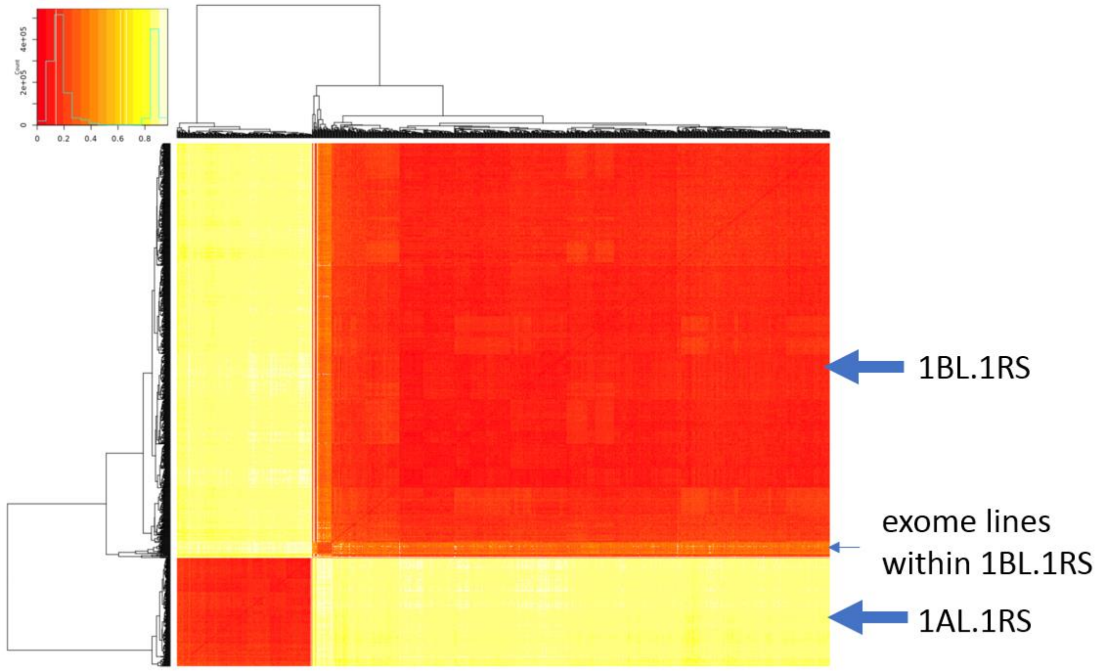

Supplementary Note: Evidence from large-scale studies relating to the yield benefits of wheat-rye translocations

Only one multi-site study has, to our knowledge, studied yield in 1RS-introgressed wheats with results relevant to the variable benefits of wheat-rye translocation lines under different climatic conditions on a large scale. This German federal agricultural trial is publicly reported only in German (https://www.bundessortenversuch.de/index.php?id=4), but not published in the peer-reviewed literature; relevant findings are therefore compiled and summarised here in English (Supplementary Note Figure 9.1).

The best overall yield was achieved by a 1RS.1AL introgression line, both with and without the application of fungicidal treatments and during a drought year, while a 1RS.1BL line in the same panel performed less well, similarly suggesting significant variability in the pathogen resistance and root morphology traits that 1RS can confer to improve yield.

Figure 9.1.

A publicly-available but unpublished multi-site study measuring yield in 1RS-introgressed wheats with results relevant to the variable benefits of wheat-rye translocation lines under different climatic conditions. The trial was conducted at 26 sites across Germany, during the exceptionally dry year 2018, and compared the performance of released wheat varieties in yield and quality metrics among wheat lines and, included two with known 1RS.1AL ('Asory') and 1RS.1BL ('Kamerad') translocations. Trials were carried out in two intensity levels, with (blue columns) and without (green columns) growth regulator and fungicide treatment. Results from trials without growth regulator and fungicide treatment (intensity level 1) are basis for the description of ripening date, plant height, stem characteristics and susceptibility to diseases. Results from the intensity level 2 with growth regulator and fungicide treatment form the basis for the description of the 4 quality classes, A – bread wheat, B – milling, C – biscuit & feed wheat, E – premium quality bread wheat. Grain yield of individual genotypes refers the mean grain yield of three reference varieties ('RGT Reform', 'Nordkap' and 'Elixer'), that is calculated for both intensities levels and set to 100%, indicated as a horizontal red line. Data source: https://www.bundessortenversuch.de/index.php?id=4.

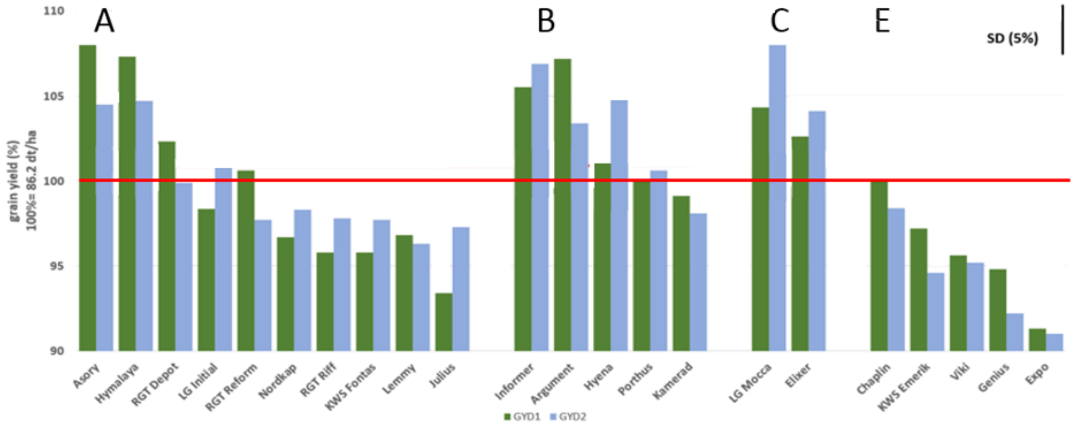

Supplementary Note 10: Comparative cluster arrangement and phylogenetics of genes with agricultural importance

Phylogenetic and homology search-based analyses (Online Methods) were applied to families of resistance genes, members of which are known to confer resistance to fungal pathogens in particular (Supplementary Note Figures 10.1—10.9), genes associated with fertility control (Supplementary Note Figure 10.10), and genes associated with low temperature tolerance (LTT; fig. 10.11). Results underlying the main arguments and conclusions of the main text are expounded in detail in the following note.

10.1 Phylogenetic and homology-based investigations of resistance gene orthologs

Nucleotide-binding leucine-rich repeats (NLR) are commonly associated with pest and pathogen resistance. We conducted a survey of NLR genes known to confer resistance to pathogens in various model systems and identified promising targets for future investigation based on their evolution within the Triticeae. For each NLR family of interest, we examined the phylogenetic relationships among members of the family within and between taxa, and used sequence homology to characterise structural changes in any notable loci containing these genes.

We identified homologs of *Pm2* and *Pm3* (two wheat powdery mildew resistance genes[62,63]), of *Mla* (an allelic series of more than 30 barley resistance genes[64]), and of wheat resistance genes *RGA2* and *Lr10*[65] in the rye, Barley[22], and Wheat[47] reference genomes.

The *Pm2* and *Pm3* genes are implicated in powdery mildew resistance in wheat[62,63], and the recognition by wheat *Pm2* of the rye powdery mildew protein *AvrPm2* suggests an active *Pm2* allele would be beneficial to rye[66]. Phylogenetic analysis revealed two clades of *Pm2*, one of which includes a resistance-associated allele known from wheat (Supplementary Note Figure 10.3). Resistant-clade *Pm2s* appear in a single copy on each group 5 wheat subgenome, but have been tandemly duplicated on rye 5R, and quadruplicated in barley 6H (Main Text Figure 4g—k; Supplementary Note Figure 10.3). The rye duplication was followed by a series of TE insertions, one of which is probably implicated in the fragmentation of one of these *Pm2* copies (Supplementary Note Figure 10.4).
Pm3 cluster on the translocation-friendly 1RS and fall into three clades (Supplementary Note Figure 10.5), the loss of one clade in rye and another in barley suggesting some inter-clade redundancy. Rye *Pm3* derives from the same clade as its orthologs on 1B, 1D, and 1H. Two further rye orthologs were identified as *Pm8* and *Pm17*, both known rye-to-wheat introgressions[67,68], the latter evidently a chimera derived from unequal crossing over between *Pm8* and an unknown *Pm*-family gene not present in ‘Lo7’ (Supplementary Note Figure 10.6).

Mla genes share close homology with wheat stem rust resistance genes *Sr33* and *Sr50*, both originally introgressions from rye 1RS, suggesting a gene family adapted to a potentially wide range of fungal interactions[69]. Three subclades can be identified (Supplementary Note Figure 10.7), and like wheat chromosome 1A, 'Lo7' contains no members of subclade 2 which contains the known resistance allele for barley; Rather, the 1R cluster is likely composed of an expanded number of subclade 1 and 3 *Mla* genes (Supplementary Note Figure 10.8).

In wheat, the leaf rust resistance locus *Lr10* occurs in two distinct haplotypes, H1 and H2, distinguished by the presence and arrangements of *Rga2* family genes[65,70]. Analysis of TE insertions suggests that haplotype H2 derives from TE-driven rearrangements of an H1-like ancestral haplotype, and the conservation of H1 and H2 among diverse wheats suggest long-term stabilising selection affecting both alleles. We identified in rye an ancient derivative of the H2 allele (Supplementary Note Figure 10.9), demonstrating the extreme length of time over which stabilisation has been occurring, and inviting further research into the reason for *Rga2-a*'s persistence across three species.

Supplementary Note Figure 10.1.

Phylogenetics and genome-wide distribution of genes in rye, broken down by subclade. A) Multidimensional scaling of the 997 NLR genes annotated in rye based on pairwise distances calculated from the phylogenetic tree shown in panel B. The colours identify three main groups of NLRs. The black dots indicate homologs of *Pm2*, *Pm3*, *Mla* and *RGA2*. B) Phylogenetic relationships between 792 complete, manually-annotated NLR genes (Supplementary Table 29). Colours correspond to the subclades in A and homologs of *Pm2*, *Pm3*, *Mla* and *RGA2* are marked.

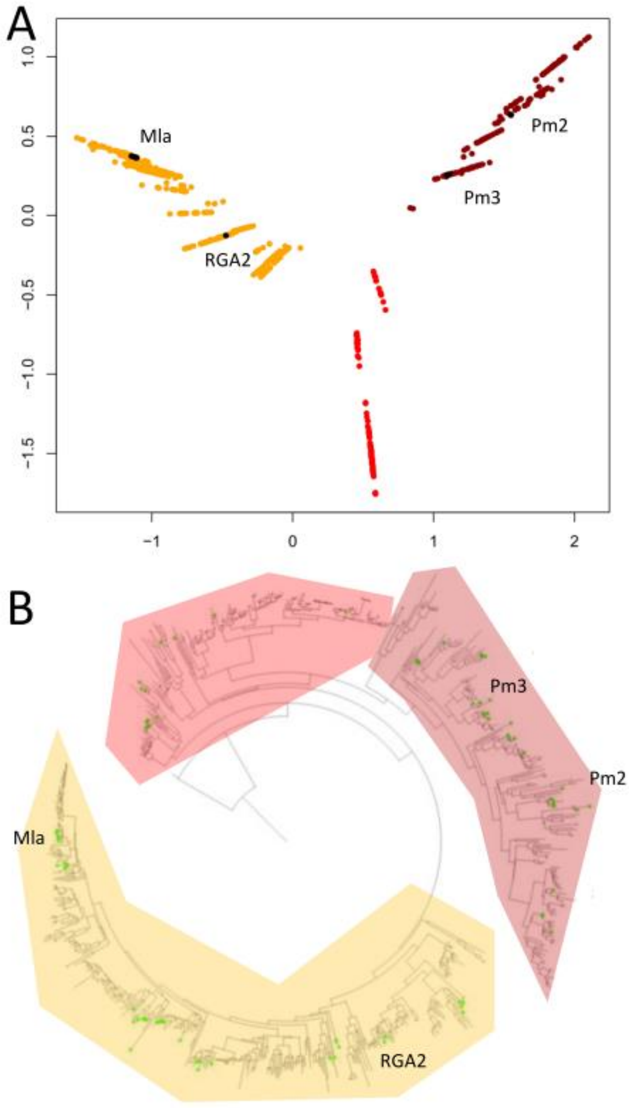

Supplementary Note Figure 10.2.

Phylogenetic relationships among full-length *Pm2* homologs in rye, wheat and barley.
The two main clades are shown in red and blue. Bootstrap support values are given at nodes.

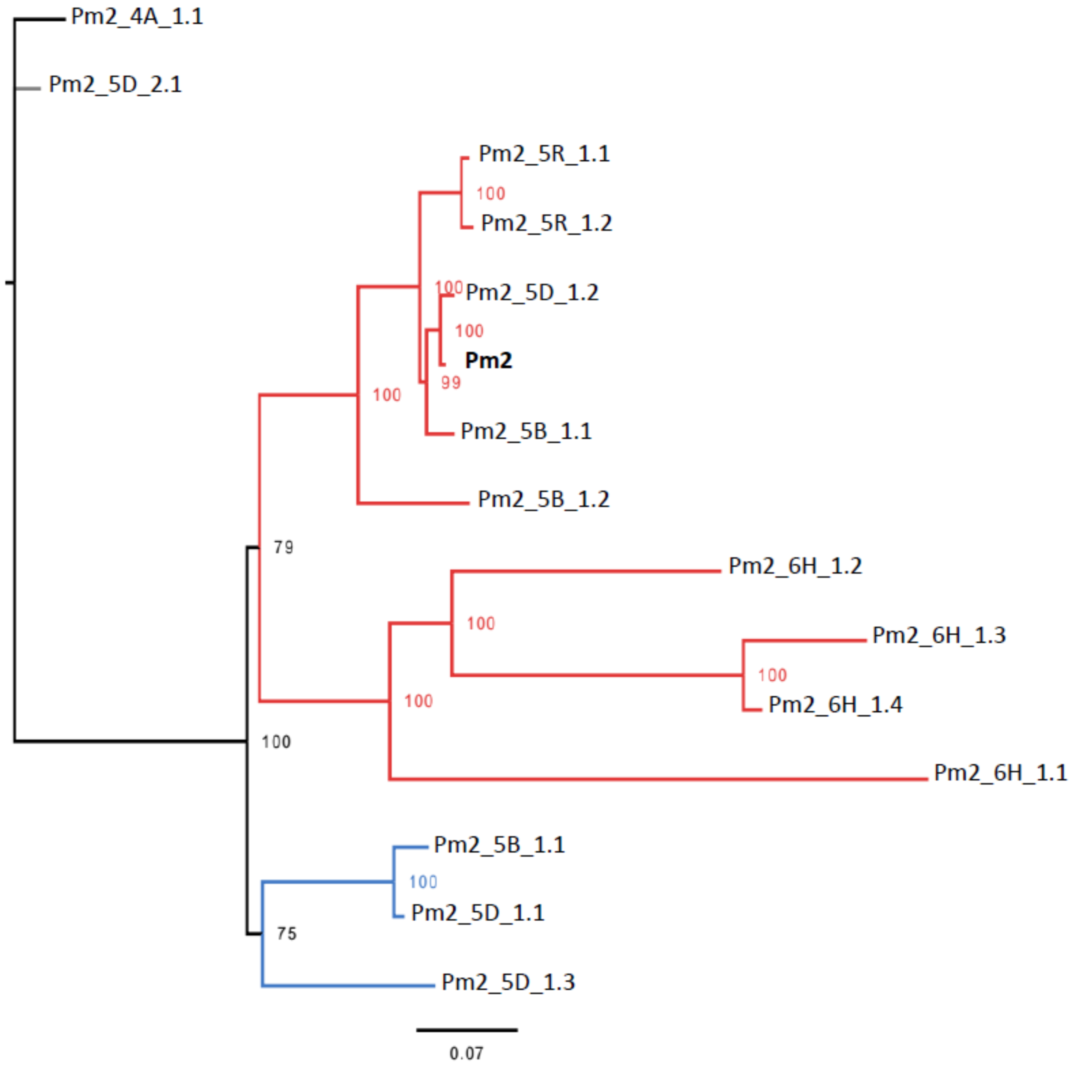

Supplementary Note Figure 10.3.

Physical organisation of *Pm2* cluster haplotypes in the (sub)genomes of cultivated Triticeae. Identifiers refer to Supplementary Note Figure 10.2 with colours separating phylogenetic clades. Pseudogenes (i.e. those which contain in-frame stop codons and/or frameshifts) are marked with a psi symbol (Ψ).

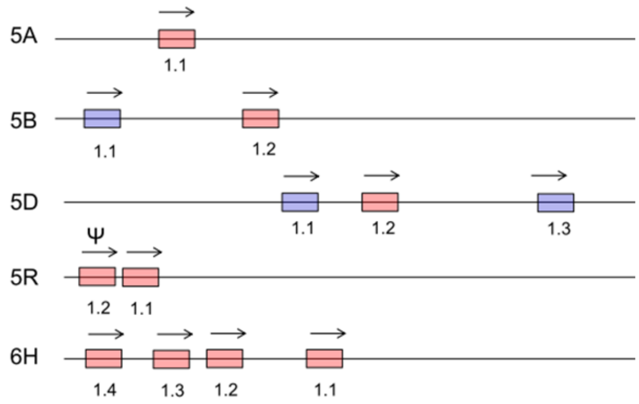

Supplementary Note Figure 10.4.

Suggested mechanism leading to the duplication and pseudogenisation of the *Pm2* locus in 'Lo7'. The two *Pm2* homologs are shown in light red, transposable elements in orange, yellow, green and purple. The two horizontal arrows indicate the duplication.

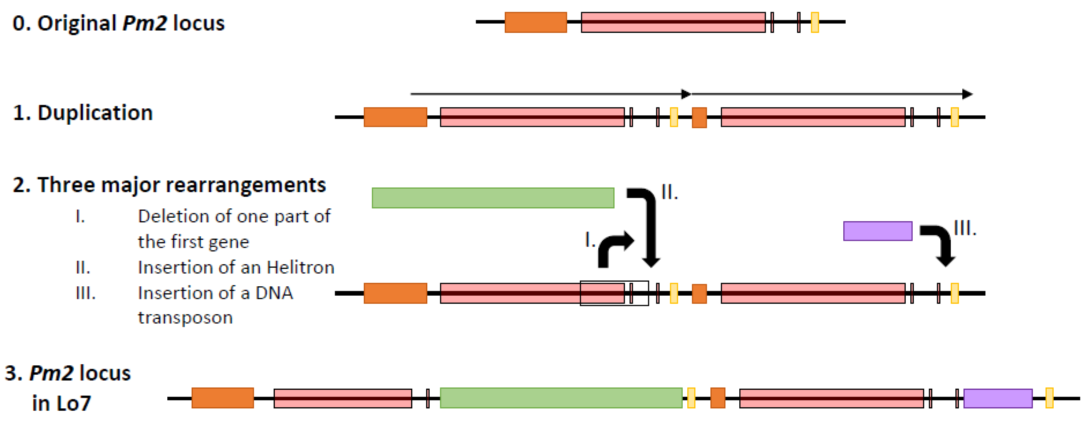

Supplementary Note Figure 10.5.

Phylogenetic relationships among full-length *Pm3* homologs in rye, wheat and barley. The three main clades are shown in red, yellow and blue. The homologs corresponding to Pm3 and Pm8 are indicated. Bootstrap support values are given at nodes. Scale bar gives substitutions per site.

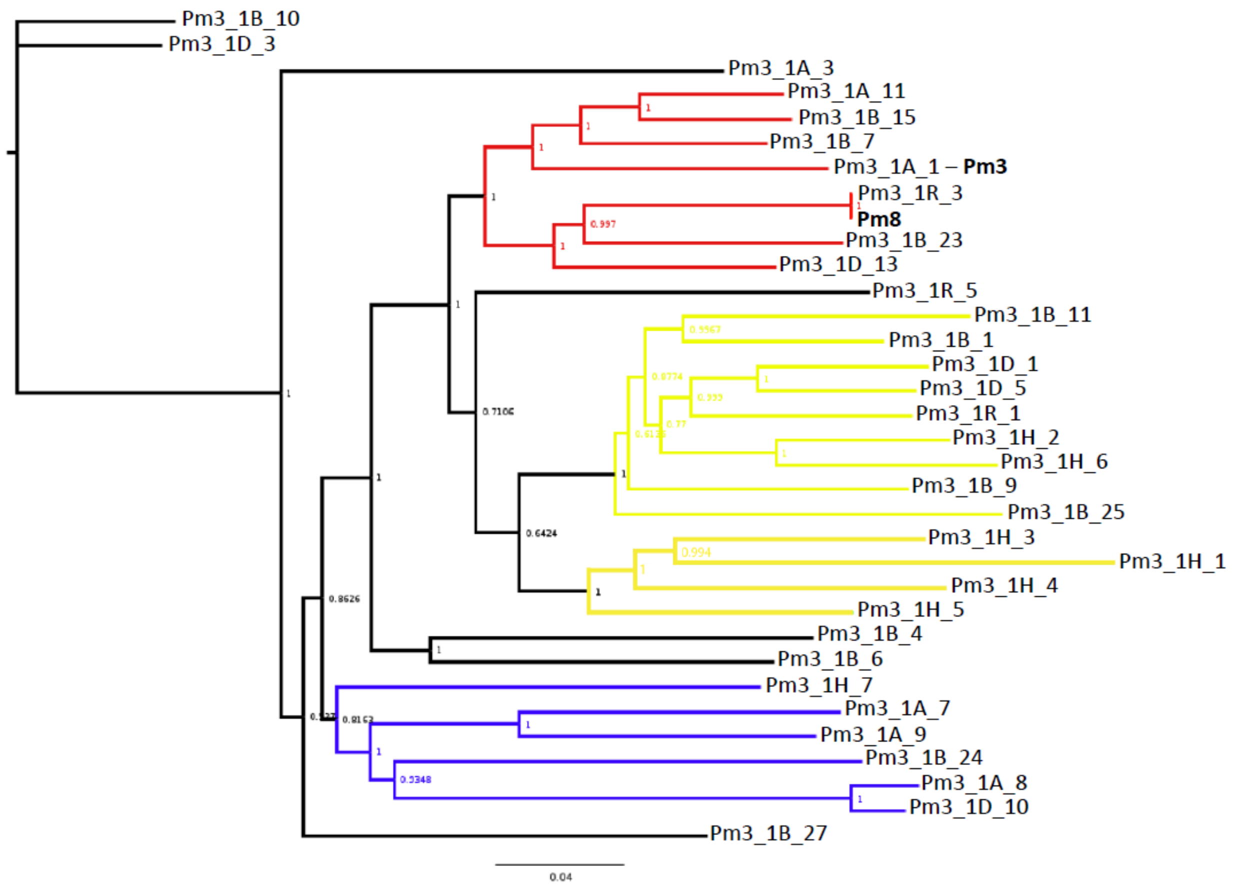

Supplementary Note Figure 10.6.

Physical organisation of the *Pm3* gene cluster haplotypes in the (sub)genomes of cultivated Triticeae. Identifiers refer to Supplementary Note Figure 10.5 with colours separating phylogenetic clades. Pseudogenes (i.e. those which contain in-frame stop codons and/or frameshifts) are marked with a psi symbol (Ψ). A novel recombinant allele, *Pm17*, which falls outside the cluster and shares homology in one portion with rye *Pm8* is also diagrammed (Supplementary Table 29).

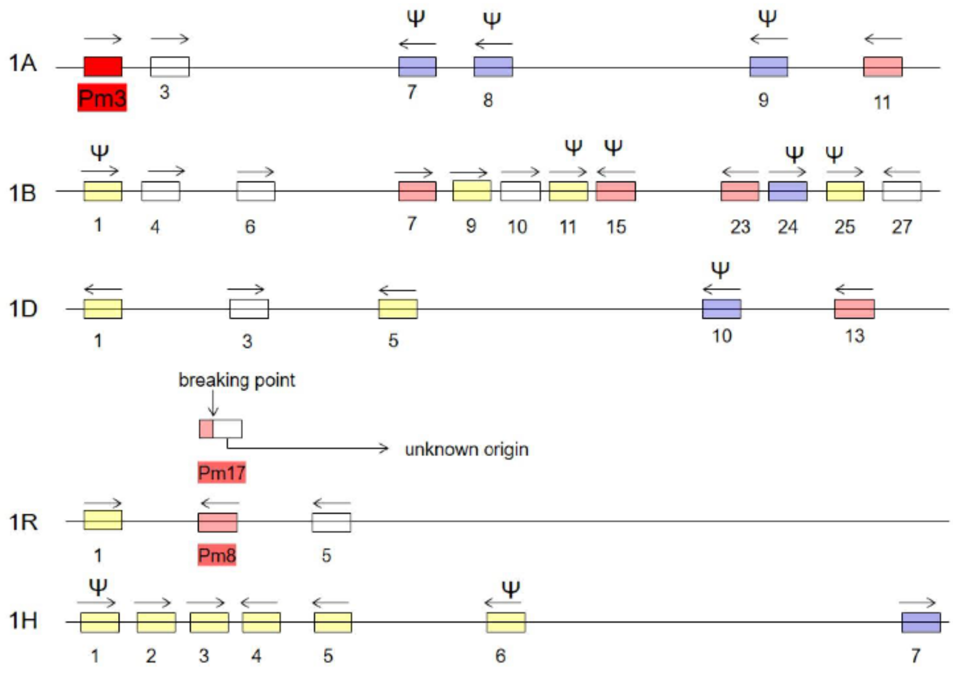

Supplementary Note Figure 10.7.

Phylogenetic relationships among full-length *Mla* homologs identified in rye, wheat and barley (including 32 known *Mla* alleles, *TmMla1*, *Sr30* and *Sr50*). The two main clades are shown in red and blue. Homologs are indicated with black names and known alleles/genes with blue or red names. Bootstrap support values are given at nodes. Scale bar gives substitutions per site.

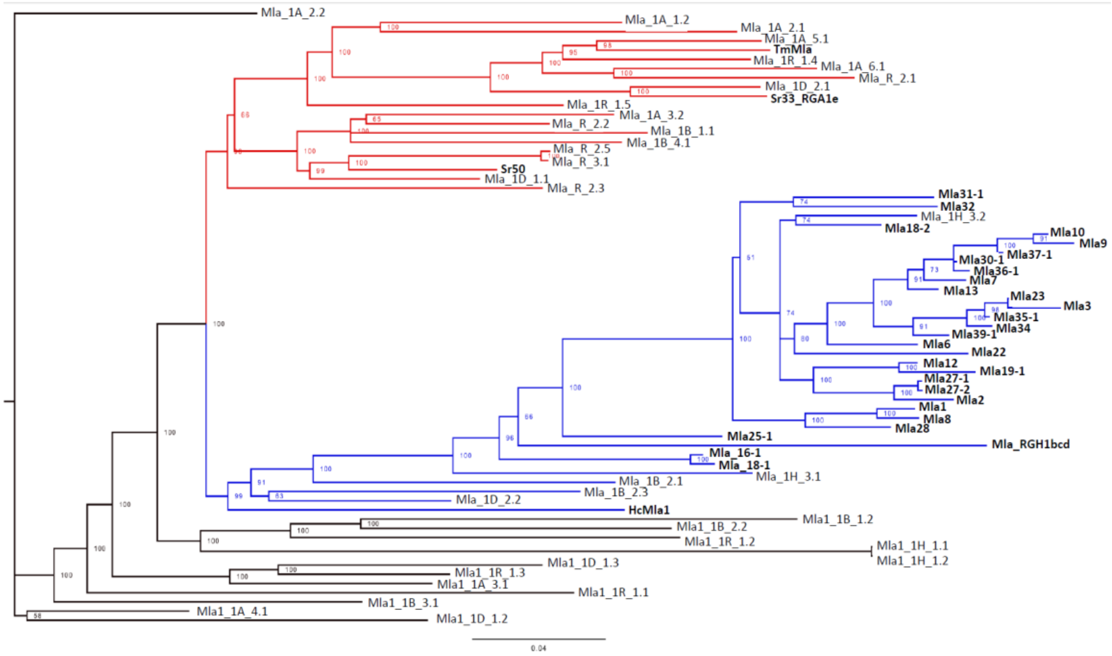

Supplementary Note Figure 10.8.

Physical organisation of *Mla* cluster haplotypes across the (sub)genomes of cultivated Triticeae. Identifiers refer to Supplementary Note Figure 10.7 and with colours separating phylogenetic clades.

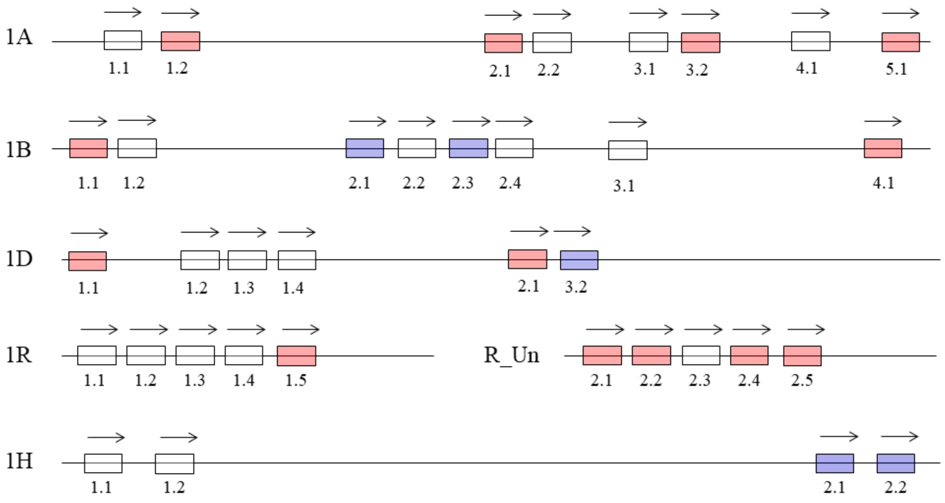

Supplementary Note Figure 10.9.

Physical organization of *Lr10* haplotypes in wheat and rye. The first two haplotypes correspond to the described *Lr10* haplotypes H1 and H2. The third represents the situation in Chinese Spring, where the H2 haplotype is present. The fourth represents the situation in 'Lo7', where some rearrangements are observed compared to the H2 haplotype.

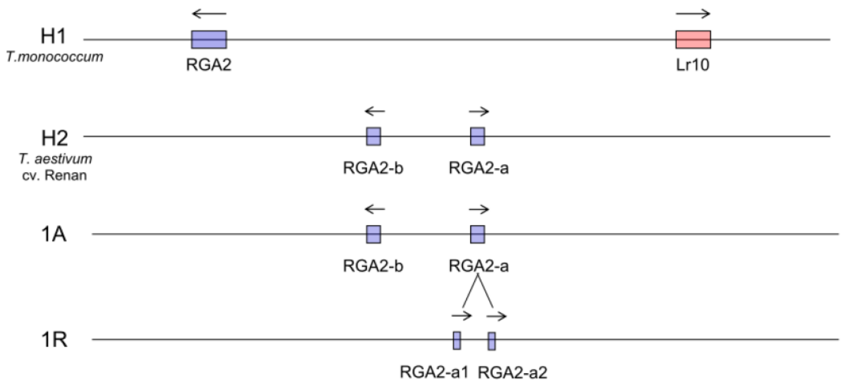

10.2 Detailed dissection of the Rf^{multi} locus in rye

Main Text Figure 4k compares the structures of the Rf^{multi} locus in rye and wheat, and we expand it here to include more detail including transcript identifiers.

Supplementary Note Figure 10.10.

Organisation of RFL genes on 1R (A) at the 'Lo7' Rf^{multi} locus (B) compared to its wheat (Chinese Spring) counterpart on 1B (C). Flanking markers are shown on either end of the rye sequence. Two full-length wheat RFLs and a putative rye ortholog are highlighted yellow. PPR genes are coloured red.

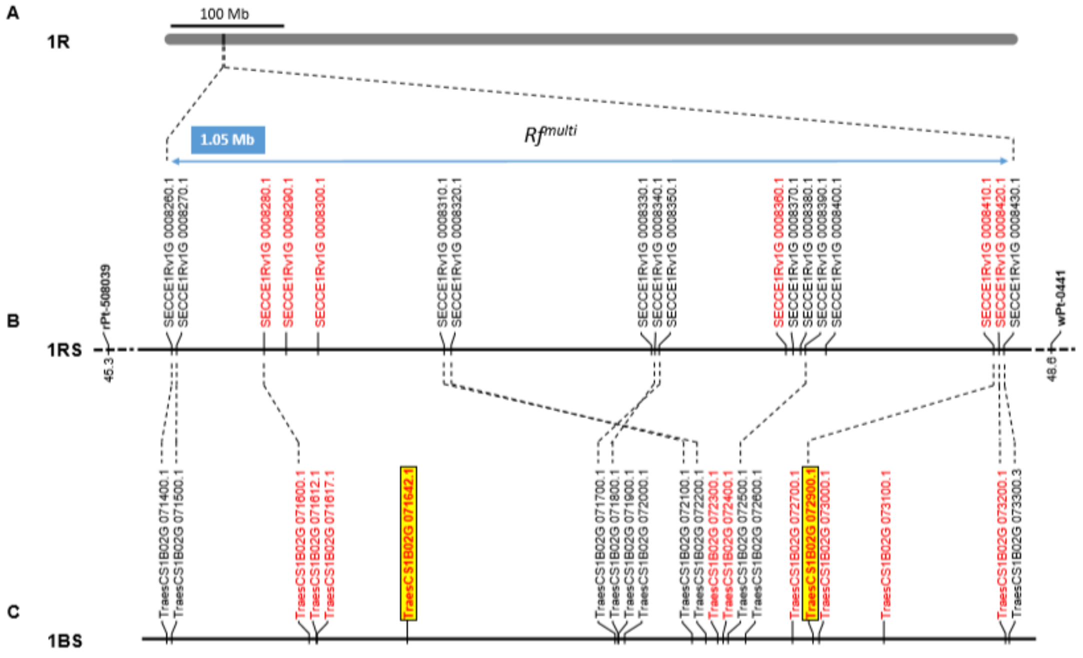

10.3 Phylogenetic and homology-based investigations of low temperature tolerance gene homologs

To investigate the possible causes of low temperature tolerance (LTT) in rye, we conducted homology searches for the LTT-associated *Cbf* family genes, a cluster of which characterise the LTT locus *Fr2* in wheat[71-74].

Supplementary Note Figure 10.11.

Homology analysis of rye protein data, showing that the *Fr2* region of 'Lo7' contains a cluster of 21 *Cbf* genes. Protein sequences for genes located between 614.3—616.5 Mbp on chromosome 5 were aligned by MUSCLE[37] and a maximum-likelihood tree was constructed by MEGAX[75]. Each *Cbf* gene is named according to its best BLASTn[27] match to previously named *Cbf* genes in 'Lo7'[76]. The position of Group IV member *Cbf-14* (similar to wheat *TaCbf-A14*), whose transcript levels predict cold tolerance, is marked[77]. Bold arrows mark *Cbf* genes for which copy number variation between 'Lo7' and 'Puma' was detected (red=strong evidence; blue=moderate evidence). Genes from the interval that are not *Cbf* genes form an outgroup. The scale bar indicates substitution rate.

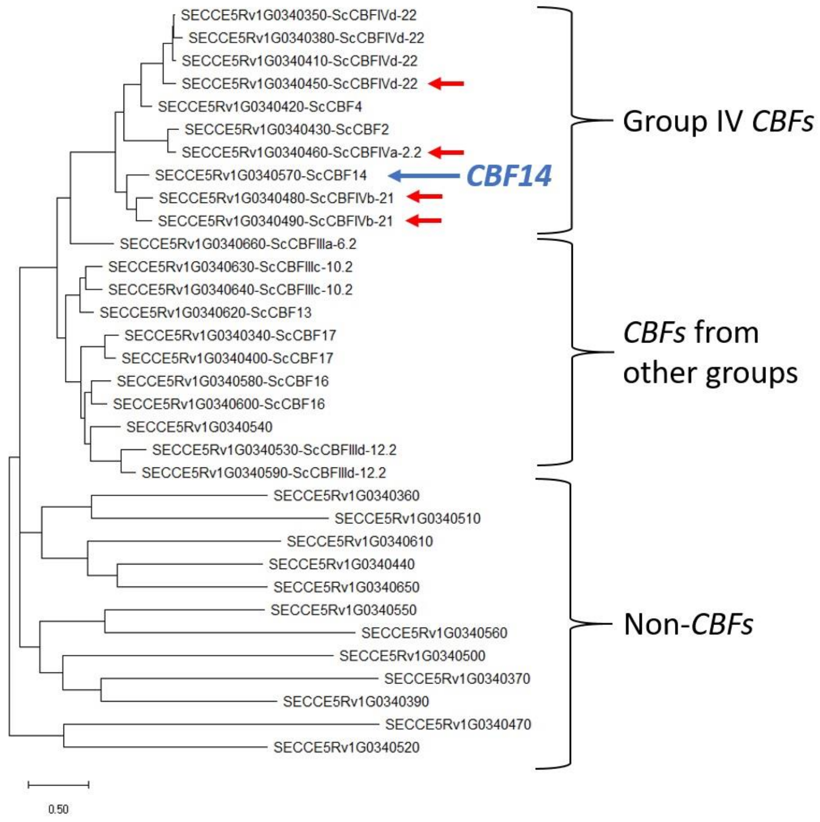

Supplementary Note References

1. Monat, C. *et al.* TRITEX: chromosome-scale sequence assembly of Triticeae genomes with open-source tools. *Genome Biology* **20**, 284 (2019).
2. Dvorak, J., McGuire, P.E. & Cassidy, B. Apparent sources of the A genomes of wheats inferred from polymorphism in abundance and restriction fragment length of repeated nucleotide sequences. *Genome* **30**, 680-689 (1988).
3. Himmelbach, A. *et al.* Discovery of multi-megabase polymorphic inversions by chromosome conformation capture sequencing in large-genome plant species. *The Plant Journal* **96**, 1309-1316 (2018).
4. Padmarasu, S., Himmelbach, A., Mascher, M. & Stein, N. *In Situ* Hi-C for Plants: An Improved Method to Detect Long-Range Chromatin Interactions. in *Plant Long Non-Coding RNAs* 441-472 (Springer, 2019).
5. Beier, S. *et al.* Construction of a map-based reference genome sequence for barley, *Hordeum vulgare* L. *Scientific data* **4**(2017).
6. Monat, C. *et al.* TRITEX: chromosome-scale sequence assembly of Triticeae genomes with open-source tools. *BioRxiv*, 631648 (2019).
7. Kubaláková, M. *et al.* Analysis and sorting of rye (*Secale cereale* L.) chromosomes using flow cytometry. *Genome* **46**, 893-905 (2003).
8. Martin, M. Cutadapt removes adapter sequences from high-throughput sequencing reads. *EMBnet. journal* **17**, 10-12 (2011).
9. Li, H. Minimap2: pairwise alignment for nucleotide sequences. *Bioinformatics* **34**, 3094-3100 (2018).
10. Simková, H. *et al.* Coupling amplified DNA from flow-sorted chromosomes to high-density SNP mapping in barley. *BMC Genomics* **9**, 294 (2008).
11. Fowler, D., N'Diaye, A., Laudencia-Chingcuanco, D. & Pozniak, C. Quantitative trait loci associated with phenological development, low-temperature tolerance, grain quality, and agronomic characters in wheat (*Triticum aestivum* L.). *PLoS One* **11**, e0152185 (2016).
12. Maccaferri, M. *et al.* Durum wheat genome highlights past domestication signatures and future improvement targets. *Nat. Genet.* **51**, 885 (2019).
13. Martinez-Viaud, K.A. *et al.* New *de novo* assembly of the Atlantic bottlenose dolphin (*Tursiops truncatus*) improves genome completeness and provides haplotype phasing. *GigaScience* **8**, giy168 (2019).
14. Guo, L. *et al.* The opium poppy genome and morphinan production. *Science* **362**, 343-347 (2018).
15. Springer, N.M. *et al.* The maize W22 genome provides a foundation for functional genomics and transposon biology. *Nat. Genet.* **50**, 1282 (2018).
16. Bauer, E. *et al.* Towards a whole-genome sequence for rye (*Secale cereale* L.). *The Plant Journal* **89**, 853--869 (2017).
17. Burton, J.N. *et al.* Chromosome-scale scaffolding of *de novo* genome assemblies based on chromatin interactions. *Nat. Biotechnol.* **31**, 1119 (2013).
18. Wu, Y., Bhat, P.R., Close, T.J. & Lonardi, S. Efficient and accurate construction of genetic linkage maps from the minimum spanning tree of a graph. *PLoS Genet.* **4**, e1000212 (2008).
19. Lieberman-Aiden, E. *et al.* Comprehensive mapping of long-range interactions reveals folding principles of the human genome. *Science* **326**, 289-293 (2009).
20. Hu, M. *et al.* HiCNorm: removing biases in Hi-C data via Poisson regression. *Bioinformatics* **28**, 3131-3133 (2012).

21. IWGSC. A chromosome-based draft sequence of the hexaploid bread wheat (*Triticum aestivum*) genome. *Science* **345**, 1251788 (2014).
22. Mascher, M. *et al.* A chromosome conformation capture ordered sequence of the barley genome. *Nature* **544**, 427--433 (2017).
23. Wu, T.D. & Watanabe, C.K. GMAP: a genomic mapping and alignment program for mRNA and EST sequences. *Bioinformatics* **21**, 1859-1875 (2005).
24. Kim, D., Langmead, B. & Salzberg, S.L. HISAT: a fast spliced aligner with low memory requirements. *Nat. Methods* **12**, 357 (2015).
25. Pertea, M. *et al.* StringTie enables improved reconstruction of a transcriptome from RNA-seq reads. *Nat. Biotechnol.* **33**, 290 (2015).
26. Ghosh, S. & Chan, C.-K.K. Analysis of RNA-Seq data using TopHat and Cufflinks. in *Plant Bioinformatics* 339-361 (Springer, 2016).
27. Altschul, S.F., Gish, W., Miller, W., Myers, E.W. & Lipman, D.J. Basic local alignment search tool. *J. Mol. Biol.* **215**, 403-410 (1990).
28. Potter, S.C. *et al.* HMMER web server: 2018 update. *Nucleic Acids Res.* **46**, W200-W204 (2018).
29. Stanke, M. *et al.* AUGUSTUS: ab initio prediction of alternative transcripts. *Nucleic Acids Res.* **34**, W435-W439 (2006).
30. Keilwagen, J., Hartung, F. & Grau, J. GeMoMa: Homology-Based Gene Prediction Utilizing Intron Position Conservation and RNA-seq Data. in *Gene Prediction* 161-177 (Springer, 2019).
31. Steinegger, M. & Söding, J. MMseqs2 enables sensitive protein sequence searching for the analysis of massive data sets. *Nat. Biotechnol.* **35**, 1026 (2017).
32. Haas, B.J. *et al.* Automated eukaryotic gene structure annotation using EVidenceModeler and the Program to Assemble Spliced Alignments. *Genome biology* **9**, R7 (2008).
33. Wicker, T. *et al.* DNA transposon activity is associated with increased mutation rates in genes of rice and other grasses. *Nature communications* **7**, 12790 (2016).
34. Rice, P., Longden, I. & Bleasby, A. EMBOSS: the European molecular biology open software suite. (Elsevier current trends, 2000).
35. Ma, J. & Bennetzen, J.L. Rapid recent growth and divergence of rice nuclear genomes. *Proceedings of the National Academy of Sciences* **101**, 12404-12410 (2004).
36. Benson, G. Tandem repeats finder: a program to analyze DNA sequences. *Nucleic Acids Res.* **27**, 573-580 (1999).
37. Edgar, R.C. MUSCLE: multiple sequence alignment with high accuracy and high throughput. *Nucleic Acids Res.* **32**, 1792-1797 (2004).
38. Kozomara, A. & Griffiths-Jones, S. miRBase: integrating microRNA annotation and deep-sequencing data. *Nucleic Acids Res.* **39**, D152-D157 (2010).
39. Akpinar, B.A., Kantar, M. & Budak, H. Root precursors of microRNAs in wild emmer and modern wheats show major differences in response to drought stress. *Functional & integrative genomics* **15**, 587-598 (2015).
40. Akpinar, B.A. *et al.* Chromosome-based survey sequencing reveals the genome organization of wild wheat progenitor *Triticum dicoccoides*. *Plant Biotechnol. J.* **16**, 2077-2087 (2018).
41. Markham, N.R. & Zuker, M. UNAFold. in *Bioinformatics* 3-31 (Springer, 2008).
42. Alptekin, B., Akpinar, B.A. & Budak, H. A comprehensive prescription for plant miRNA identification. *Frontiers in plant science* **7**, 2058 (2017).
43. Lucas, S.J. & Budak, H. Sorting the wheat from the chaff: identifying miRNAs in genomic survey sequences of *Triticum aestivum* chromosome 1AL. *PLoS One* **7**, e40859 (2012).
44. Dai, X., Zhuang, Z. & Zhao, P.X. psRNATarget: a plant small RNA target analysis server (2017 release). *Nucleic Acids Res.* **46**, W49-W54 (2018).

45. Dai, X. & Zhao, P.X. psRNATarget: a plant small RNA target analysis server. *Nucleic Acids Res.* **39**, W155-W159 (2011).
46. Conesa, A. *et al.* Blast2GO: a universal tool for annotation, visualization and analysis in functional genomics research. *Bioinformatics* **21**, 3674-3676 (2005).
47. IWGSC. Shifting the limits in wheat research and breeding using a fully annotated reference genome. *Science* **361**, eaar7191 (2018).
48. Sonnhammer, E.L. & Durbin, R. A dot-matrix program with dynamic threshold control suited for genomic DNA and protein sequence analysis. *Gene* **167**, GC1-GC10 (1995).
49. Thompson, J.D., Gibson, T.J. & Higgins, D.G. Multiple sequence alignment using ClustalW and ClustalX. *Current protocols in bioinformatics*, 2.3. 1-2.3. 22 (2003).
50. Ronquist, F. *et al.* MrBayes 3.2: efficient Bayesian phylogenetic inference and model choice across a large model space. *Syst. Biol.* **61**, 539-542 (2012).
51. Paradis, E. & Schliep, K. ape 5.0: an environment for modern phylogenetics and evolutionary analyses in R. *Bioinformatics* **35**, 526-528 (2018).
52. Finn, R.D., Clements, J. & Eddy, S.R. HMMER web server: interactive sequence similarity searching. *Nucleic Acids Res.* **39**, W29-W37 (2011).
53. Cheng, S. *et al.* Redefining the structural motifs that determine RNA binding and RNA editing by pentatricopeptide repeat proteins in land plants. *The Plant Journal* **85**, 532-547 (2016).
54. El-Gebali, S. *et al.* The Pfam protein families database in 2019. *Nucleic Acids Res.* **47**, D427-D432 (2018).
55. Hackauf, B., Rudd, S., Van der Voort, J., Miedaner, T. & Wehling, P. Comparative mapping of DNA sequences in rye (*Secale cereale* L.) in relation to the rice genome. *Theor. Appl. Genet.* **118**, 371-384 (2009).
56. Steuernagel, B. *et al.* The NLR-Annotator tool enables annotation of the intracellular immune receptor repertoire. **183**, 468-482 (2020).
57. Ou, S., Chen, J. & Jiang, N. Assessing genome assembly quality using the LTR Assembly Index (LAI). *Nucleic Acids Res.* **46**, e126-e126 (2018).
58. Schnable, P.S. *et al.* The B73 maize genome: complexity, diversity, and dynamics. *science* **326**, 1112-1115 (2009).
59. IBSC. A physical, genetic and functional sequence assembly of the barley genome. (2012).
60. Martis, M.M. *et al.* Reticulate evolution of the rye genome. *The Plant Cell* **25**, 3685-3698 (2013).
61. Avni, R. *et al.* Wild emmer genome architecture and diversity elucidate wheat evolution and domestication. *Science* **357**, 93--97 (2017).
62. Yahiaoui, N., Srichumpa, P., Dudler, R. & Keller, B. Genome analysis at different ploidy levels allows cloning of the powdery mildew resistance gene *Pm3b* from hexaploid wheat. *The Plant Journal* **37**, 528-538 (2004).
63. Sánchez-Martín, J. *et al.* Rapid gene isolation in barley and wheat by mutant chromosome sequencing. *Genome biology* **17**, 221 (2016).
64. Seeholzer, S. *et al.* Diversity at the *Mla* powdery mildew resistance locus from cultivated barley reveals sites of positive selection. *Mol. Plant-Microbe Interact.* **23**, 497-509 (2010).
65. Feuillet, C. *et al.* Map-based isolation of the leaf rust disease resistance gene *Lr10* from the hexaploid wheat (*Triticum aestivum* L.) genome. *Proceedings of the National Academy of Sciences* **100**, 15253-15258 (2003).
66. Praz, C.R. *et al.* *AvrPm2* encodes an RNase-like avirulence effector which is conserved in the two different specialized forms of wheat and rye powdery mildew fungus. *New Phytol.* **213**, 1301-1314 (2017).

67. Hurni, S. *et al.* Rye *Pm8* and wheat *Pm3* are orthologous genes and show evolutionary conservation of resistance function against powdery mildew. *The Plant Journal* **76**, 957-969 (2013).
68. Singh, S.P. *et al.* Evolutionary divergence of the rye *Pm17* and *Pm8* resistance genes reveals ancient diversity. *Plant Mol. Biol.* **98**, 249-260 (2018).
69. Mago, R. *et al.* The wheat *Sr50* gene reveals rich diversity at a cereal disease resistance locus. *Nature plants* **1**, 15186 (2015).
70. Isidore, E., Scherrer, B., Chalhoub, B., Feuillet, C. & Keller, B. Ancient haplotypes resulting from extensive molecular rearrangements in the wheat A genome have been maintained in species of three different ploidy levels. *Genome Res.* **15**, 526-536 (2005).
71. Babben, S. *et al.* Association genetics studies on frost tolerance in wheat (*Triticum aestivum* L.) reveal new highly conserved amino acid substitutions in *CBF-A3*, *CBF-A15*, *VRN3* and *PPD1* genes. *BMC Genomics* **19**, 409 (2018).
72. Campoli, C., Matus-Cádiz, M.A., Pozniak, C.J., Cattivelli, L. & Fowler, D.B. Comparative expression of *Cbf* genes in the Triticeae under different acclimation induction temperatures. *Mol. Genet. Genomics* **282**, 141-152 (2009).
73. Vágújfalvi, A., Galiba, G., Cattivelli, L. & Dubcovsky, J. The cold-regulated transcriptional activator *Cbf3* is linked to the frost-tolerance locus *Fr-A2* on wheat chromosome 5A. *Mol. Genet. Genomics* **269**, 60-67 (2003).
74. Würschum, T., Longin, C.F.H., Hahn, V., Tucker, M.R. & Leiser, W.L. Copy number variations of *CBF* genes at the *Fr-A2* locus are essential components of winter hardiness in wheat. *The Plant Journal* **89**, 764-773 (2017).
75. Kumar, S., Stecher, G., Li, M., Knyaz, C. & Tamura, K. MEGA X: molecular evolutionary genetics analysis across computing platforms. *Mol. Biol. Evol.* **35**, 1547-1549 (2018).
76. Jung, W.J. & Seo, Y.W. Identification of novel C-repeat binding factor (*CBF*) genes in rye (*Secale cereale* L.) and expression studies. *Gene* **684**, 82-94 (2019).
77. Zhu, J. *et al.* Copy number and haplotype variation at the *VRN-A1* and central *FR-A2* loci are associated with frost tolerance in hexaploid wheat. *Theor. Appl. Genet.* **127**, 1183-1197 (2014).
