# Supplementary material for: Chromosome-scale genome assembly provides insights into rye biology, evolution and agronomic potential
Source: Nat Genet. 2021 Mar 18;53(4):564–73. doi: 10.1038/s41588-021-00807-0 (PMC8035072; doi:10.1038/s41588-021-00807-0)
Supplement: Supplementary file 2 — Reporting Summary [file 41588_2021_807_MOESM2_ESM.pdf]

nature research

Corresponding author(s): Nils Stein

Last updated by author(s): Dec 4, 2020

Reporting Summary

Nature Research wishes to improve the reproducibility of the work that we publish. This form provides structure for consistency and transparency in reporting. For further information on Nature Research policies, see our Editorial Policies and the Editorial Policy Checklist.

Statistics

For all statistical analyses, confirm that the following items are present in the figure legend, table legend, main text, or Methods section.

n/a	Confirmed	
☐	☒	The exact sample size (*n*) for each experimental group/condition, given as a discrete number and unit of measurement
☐	☒	A statement on whether measurements were taken from distinct samples or whether the same sample was measured repeatedly
☐	☒	The statistical test(s) used AND whether they are one- or two-sided<br>*Only common tests should be described solely by name; describe more complex techniques in the Methods section.*
☐	☒	A description of all covariates tested
☐	☒	A description of any assumptions or corrections, such as tests of normality and adjustment for multiple comparisons
☐	☒	A full description of the statistical parameters including central tendency (e.g. means) or other basic estimates (e.g. regression coefficient) AND variation (e.g. standard deviation) or associated estimates of uncertainty (e.g. confidence intervals)
☐	☒	For null hypothesis testing, the test statistic (e.g. *F*, *t*, *r*) with confidence intervals, effect sizes, degrees of freedom and *P* value noted<br>*Give P values as exact values whenever suitable.*
☒	☐	For Bayesian analysis, information on the choice of priors and Markov chain Monte Carlo settings
☐	☒	For hierarchical and complex designs, identification of the appropriate level for tests and full reporting of outcomes
☐	☒	Estimates of effect sizes (e.g. Cohen's *d*, Pearson's *r*), indicating how they were calculated

Our web collection on statistics for biologists contains articles on many of the points above.

Software and code

Policy information about availability of computer code

Data collection	The majority of software used in the study was unambiguously used in data 'analysis', but some software could be said to overlap with data 'collection' (e.g. adapter trimming with cutadapt, or demultiplexing with splitgbs). For simplicity, we have listed all software used in the "Data Analysis" field.
Data analysis	Published software used are listed below. Specific usage details are given in the methods.<br><br>10X Loupe browser 2.1.1<br>10X LongRanger v2.2.0<br>AHRD v1.6<br>Augustus v3.3.2<br>bbduk v37.28<br>bcftools v1.9<br>Bionano Solve v3.1<br>Blast2GO software v5.2<br>bwa v0.7<br>cn.mops 1.12.0<br>CNVnator 0.4<br>Cuffcompare v2.2.1<br>cutadapt v1.9.1<br>DeNovoMagic3.0 assembly pipeline (proprietary, NRGene Israel)<br>DESeq2 v3.11<br>dotter v4.22<br>EIGENSOFT v6.0.1<br>EMBOSS package v6.6 (Incl. MUSCLE, WATER, and ClustalW, GetORF)

EvidenceModeller v1.1.1
GeMoMa v1.5.3
GenomeThreader v1.7.1
GMAP v2017-01-14
Hisat2 v2.1.0
Hisat2 v2.1.0
HMMER v3.2.1
hmmscan v3.2.1
htseq v11.1
minimap2 v2.1
mmseqs2 Release 8-fac81
ncbi-blast-2.3.0+/2.3.1+/2.8.1+/2.9.0+
NCSS 97
NLR-Annotator Pipeline, last pulled September 2018
PacBio SMRTlink v5.1.0
RepeatMasker v4.0
samtools v1.9
SnpEff v4
Stringtie v1.3.6
TandemRepeatsFinder v4.09
Transdecoder v3.0.0
TRITEX Pipeline version corresponding to commit ID 2898e74, with minor function modifications and nontrivial plotting tasks available in a sourceable, annotated R function collection available at https://github.com/mtrw/Sc_genome_assembly
vmatch dbcluster v2.3.0

R v3.4.2
R packages (dependencies not listed; all updated including dependencies to their latest available CRAN/Bioconductor versions on 1 February 2018):
parallel
mhsmm
ASMap
SNPRelate
plyr
dplyr
magrittr
data.table
ggplot2
colorspace
zoo
stringi
igraph
ape
kernlab
e1071
lmerTest

In-house scripts:

get_alleles_at_position.c (https://github.com/mtrw/tim_bioinfo_tools/blob/master/blast_get_alleles_at_position.c)
blast_to_snps.c (https://github.com/mtrw/tim_bioinfo_tools/blob/master/blast_to_snps.c)
splitgbs.c (github.com/umngao/splitgbs)
SUmirFind.pl, SUmirFold.pl, SUmirScreen_v2.py, and SUmirLocate_v2.py, all available at https://github.com/hikmetbudak/miRNA-annotation

For manuscripts utilizing custom algorithms or software that are central to the research but not yet described in published literature, software must be made available to editors and reviewers. We strongly encourage code deposition in a community repository (e.g. GitHub). See the Nature Research guidelines for submitting code & software for further information.

Data

Policy information about availability of data

All manuscripts must include a data availability statement. This statement should provide the following information, where applicable:

- Accession codes, unique identifiers, or web links for publicly available datasets
- A list of figures that have associated raw data
- A description of any restrictions on data availability

The 'Lo7' assembly and gene feature annotation data are available via e!DAL with DOIs 10.5447/ipk/2020/33 and 10.5447/ipk/2020/29. The visual suite of resources for assembly assessment are stored at 10.5447/ipk/2020/32. Raw sequence data generated in the course of the study are available at ENA with accession numbers PRJEB32636 (PE and MP data for assembly), PRJEB32574 and PRJEB34626 (Hi-C), PRJEB34439 (10X), PRJEB32587 (CSS), PRJEB35392 (GBS data), and PRJEB35461 (RNAseq and IsoSeq for annotation of 'Lo7'). Chromium 10X and RNAseq data for 'Puma' and 'Norstar' are available at PRJNA564622. The SNP matrix used for rye population genetic analyses is available via e!DAL with DOI 10.5447/ipk/2020/31. GBS and sequence data generated for the USDA and CIMMYT wheat diversity panels are available at ENA with accession numbers PRJNA566410, PRJNA566408, and PRJNA566409. Optical map data and alignments are available at via e!DAL with DOI 10.5447/ipk/2020/30. High-stringency transposable element annotations (used for evolutionary analyses) are given in Supplementary Table 10, while the larger, low-stringency annotations (used for assembly quality comparisons) are available via e!DAL with DOI 10.5447/ipk/2020/34.

Field-specific reporting

Please select the one below that is the best fit for your research. If you are not sure, read the appropriate sections before making your selection.

☒ Life sciences ☐ Behavioural & social sciences ☐ Ecological, evolutionary & environmental sciences

For a reference copy of the document with all sections, see nature.com/documents/nr-reporting-summary-flat.pdf

Life sciences study design

All studies must disclose on these points even when the disclosure is negative.

Sample size	Various, for the different studies described in the manuscript. The effects of introgression of yield analysis involves 19,702 observations of 2,164 genotypes over 78 sites and 26 years. The flow cytometry was performed on five instances of each sample, each measured three times on different days. Gene expression profiling sequencing and cold hardiness of Norstar wheat and Puma rye lines were each measured along 12 time stages, the RNA being sampled from two plants at each. LT50 was measured in five individuals in each of three experimental replicate groups for each of for each of five pre-selected test temperatures for each line at each time point. Extended details are given in the manuscript materials.<br><br>An all studies reported in this paper, sample sizes were maximised within the constrains of practicability, e.g. availability of germplasms/ genotypes, cost of sequencing, availability of field and greenhouse space etc.
Data exclusions	No data were excluded from the analyses reported.
Replication	No whole-experiment replication (of the kind that is standard in classical clinical trial design) was conducted in the agricultural, cytogenetic, and in silico studies described in this paper.
Randomization	Of the studies reported, randomisation is relevant only to the study on cold acclimation in rye and wheat plants. Norstar and Puma plants sampled for the expression and cold acclimation studies were grown in a randomised complete block design, with the three replicates separated in time and space between blocks.
Blinding	No blinding (of the kind that is standard in classical clinical trial design) was conducted in the agricultural, cytogenetic, and in silico studies described in this paper.

Reporting for specific materials, systems and methods

We require information from authors about some types of materials, experimental systems and methods used in many studies. Here, indicate whether each material, system or method listed is relevant to your study. If you are not sure if a list item applies to your research, read the appropriate section before selecting a response.

Materials & experimental systems

n/a	Involved in the study
☒	☐ Antibodies
☒	☐ Eukaryotic cell lines
☒	☐ Palaeontology and archaeology
☒	☐ Animals and other organisms
☒	☐ Human research participants
☒	☐ Clinical data
☒	☐ Dual use research of concern

Methods

n/a	Involved in the study
☒	☐ ChIP-seq
☐	☒ Flow cytometry
☒	☐ MRI-based neuroimaging

Flow Cytometry

Plots

Confirm that:

☐ The axis labels state the marker and fluorochrome used (e.g. CD4-FITC).

☐ The axis scales are clearly visible. Include numbers along axes only for bottom left plot of group (a 'group' is an analysis of identical markers).

☐ All plots are contour plots with outliers or pseudocolor plots.

☐ A numerical value for number of cells or percentage (with statistics) is provided.

Methodology

Sample preparation	Grains from fifteen diverse rye accessions were provided by nine providers listed in the supplementary tables. Plants of pea served as an internal reference standard in flow cytometric estimation of nuclear DNA content in all accessions, except of the tetraploid accession ACE-1, for which S. cereale line 'Lo7' was used as a reference. Seeds of pea (Pisum sativum cv. Ctirad) were obtained from Semo (Smržice, Czech Republic) breeding station. Plants were raised in garden compost in pots and maintained in a greenhouse until they reached a height of 10–20 cm. Ten mg of fresh leaf tissue of each of the rye accessions and the reference standard were chopped together in a 1 mL volume of LB01 solution2 using a razor blade. The resulting homogenate was filtered through a 50 µm nylon mesh. The filtrate was made up to 50 µg/mL propidium iodide and 50 µg/mL RNase.
Instrument	CyFlow Space flow cytometer (Sysmex Partec GmbH, Görlitz, Germany) equipped with a 532 nm green laser.
Software	The NCSS 97 statistical software package (Statistical Solutions Ltd.)
Cell population abundance	N/A. The gain of the instrument was adjusted so that the peak representing G1 nuclei of the genome size standard was positioned approximately on channel 100 on a histogram of relative fluorescence intensity when using a 512-channel scale.
Gating strategy	N/A, since the aim of the experiment was not to separate cell types. No plots or figures are included in association with the genome size estimation by flow cytometry. Data are available in the supplementary tables S1.

☐ Tick this box to confirm that a figure exemplifying the gating strategy is provided in the Supplementary Information.
